# Regioselective Synthesis of 5- and 3-Hydroxy-*N*-Aryl-1*H*-Pyrazole-4-Carboxylates and Their Evaluation as Inhibitors of *Plasmodium falciparum* Dihydroorotate Dehydrogenase

**DOI:** 10.3390/molecules27154764

**Published:** 2022-07-25

**Authors:** Luka Vah, Tadej Medved, Uroš Grošelj, Marina Klemenčič, Črtomir Podlipnik, Bogdan Štefane, Jernej Wagger, Marko Novinec, Jurij Svete

**Affiliations:** Faculty of Chemistry and Chemical Technology, University of Ljubljana, Večna pot 113, 1000 Ljubljana, Slovenia; luka.vah@novartis.com (L.V.); tm6300@student.uni-lj.si (T.M.); uros.groselj@fkkt.uni-lj.si (U.G.); marina.klemencic@fkkt.uni-lj.si (M.K.); crtomir.podlipnik@fkkt.uni-lj.si (Č.P.); bogdan.stefane@fkkt.uni-lj.si (B.Š.); jernej.wagger@novartis.com (J.W.)

**Keywords:** malaria, pyrazolones, hydrazines, dihydroorotate dehydrogenase, enzyme inhibition

## Abstract

In silico evaluation of various regioisomeric 5- and 3-hydroxy-substituted alkyl 1-aryl-1*H*-pyrazole-4-carboxylates and their acyclic precursors yielded promising results with respect to their binding in the active site of dihydroorotate dehydrogenase of *Plasmodium falciparum* (*Pf*DHODH). Consequently, four ethyl 1-aryl-5-hydroxy-1*H*-pyrazole-4-carboxylates and their 3-hydroxy regioisomers were prepared by two-step syntheses via enaminone-type reagents or key intermediates. The synthesis of 5-hydroxy-1*H*-pyrazoles was carried out using the literature protocol comprising acid-catalyzed transamination of diethyl [(dimethylamino)methylene]malonate with arylhydrazines followed by base-catalyzed cyclization of the intermediate hydrazones. For the synthesis of isomeric methyl 1-aryl-3-hydroxy-1*H*-pyrazole-4-carboxylates, a novel two-step synthesis was developed. It comprises acylation of hydrazines with methyl malonyl chloride followed by cyclization of the hydrazines with *tert*-butoxy-bis(dimethylamino)methane. Testing the pyrazole derivatives for the inhibition of *Pf*DHODH showed that 1-(naphthalene-2-yl)-5-hydroxy-1*H*-pyrazole-4-carboxylate and 1-(naphthalene-2-yl)-, 1-(2,4,6-trichlorophenyl)-, and 1-[4-(trifluoromethyl)phenyl]-3-hydroxy-1*H*-pyrazole-4-carboxylates (~30% inhibition) were slightly more potent than a known inhibitor, diethyl α-{[(1*H*-indazol-5-yl)amino]methylidene}malonate (19% inhibition).

## 1. Introduction

Malaria is a serious infectious disease that is endemic in many countries, especially in tropical regions. It threatens a significant portion of the world’s population, with over three billion people at risk of infection in 2013. It is also an important public health problem, with over 200 million clinical cases and 500,000 deaths per year [1]. The emergence of drug-resistant strains of *Plasmodium falciparum* (*P. falciparum*) has intensified the research and development of new drugs and therapies for malaria treatment [1,2,3,4,5,6]. Antimalarials with artemisinin-based combination therapies (ACTs) have been successful in the majority of malaria cases for some time, but artemisinin efficacy has been shown to decline in Southeast Asia, jeopardizing recent successes [7]. In some areas, *P. falciparum* has become resistant to all clinically approved antimalarials. As current trends clearly indicate that malarial disease will continue to impact global health, the discovery and development of new, effective antimalarial drugs is urgently needed.

**Figure 1:**
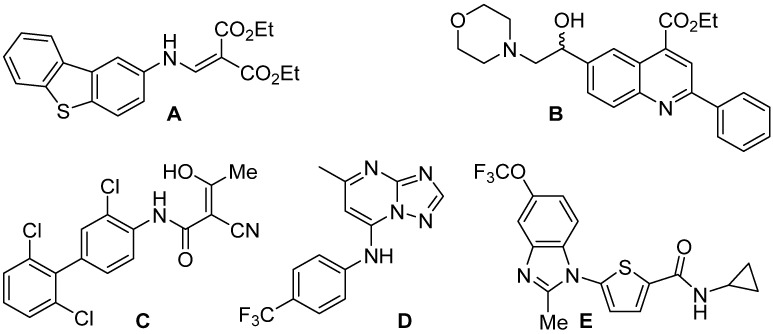
Compounds **A**–**E** as examples of known inhibitors of *Pf*DHODH.

Targeting the mitochondrial functions of *P. falciparum* has been successful for enzymes within the mitochondrial electron transport chain. *Plasmodium falciparum* dihydroorotate dehydrogenase (*Pf*DHODH) is the central enzyme in the de novo synthesis of pyrimidine nucleotides and thus key to parasite survival. This makes *Pf*DHODH an attractive target for drug development to treat malaria [8,9,10,11]. Several types of selective inhibitors of *Pf*DHODH are known, which are much less active compared to human DHODH. These include malonate (**A**) [12], an analogue of brequinar (**B**) [13], an analogue of leflunomide (**C**) [14], 5-methyl-7-{[4-(trifluoromethyl)phenyl]amino}[1,2,4]triazolo[1,5-*a*]pyrimidine (**D**) [15], 3-(benzimidazolyl)thiophene derivative **E** [16], and others [8] (Figure 1).

**Figure 2:**
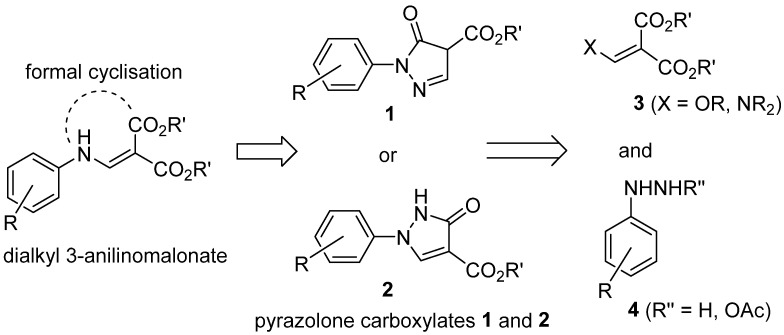
Dialkyl 3-anilinomalonate and its conformationally constrained pyrazolone analogues **1** and **2**.

Heterocyclic building blocks are useful scaffolds for applications in medicinal chemistry, catalysis, and materials science [17,18,19,20]. In medicinal chemistry, heterocyclic systems are commonly used to restrict the conformational flexibility of small molecules in order to increase their biological activity through enhanced interactions with the active site of the target macromolecule. Using malonate **A**, a known selective inhibitor of *Pf*DHODH, as the lead compound [12] (cf. Figure 1), we reasoned that its conformationally constrained pyrazole analogues might also be selective inhibitors of *Pf*DHODH, also because of the known examples of pyrazole derivatives with antimalarial activity [21,22,23,24,25]. As shown in Figure 2, formal cyclization of dialkyl 3-anilinomalonate leads to two isomeric alkyl pyrazolone carboxylates **1** and **2**, which in turn are accessible by cyclocondensation methods with monosubstituted hydrazines **4** and α-alkoxymethylidene- or α-[(dimethylamino)methylidene]malonates **3** [26].

**Scheme 1:**
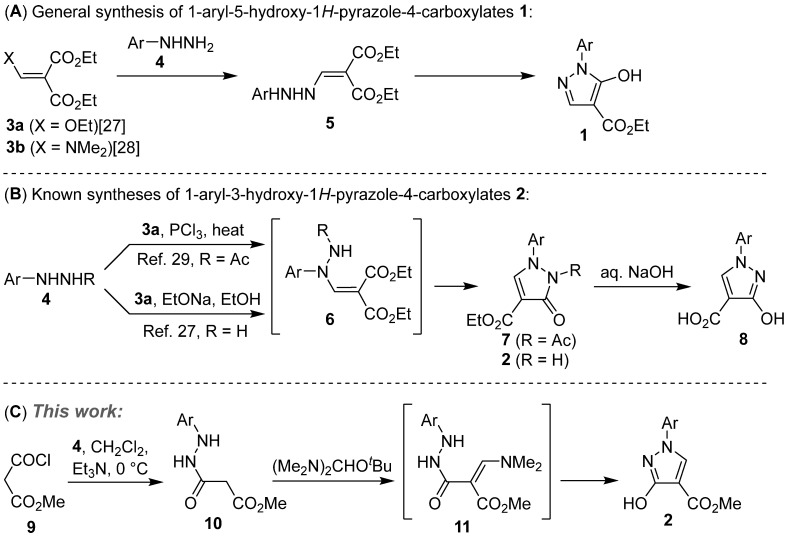
(**A**,**B**): Known syntheses of alkyl 1-aryl-1*H*-pyrazole-4-carboxylates **1** [27,28] and **2** [27,29]. (**C**): A novel synthesis of **2** reported in this paper.

As shown in Scheme 1, 1-aryl-5-hydroxy-1*H*-pyrazole-4-carboxylates **1** are readily available by condensation of arylhydrazines **4** with diethyl α-(ethoxymethylidene)malonate (**3a**) [27] or diethyl α-[(dimethylamino)methylidene]malonate (**3b**) [28] via enhydrazine intermediates **5** (Scheme 1A). In contrast, the synthesis of isomeric 1-aryl-3-hydroxy-1*H*-pyrazole-4-carboxylates **2** from methylidene malonates **3** is difficult because the less nucleophilic amino group of **4** must first react with the electrophile **3**. So far, two general methods for the synthesis of **2** have been reported. The first method is a three-step synthesis in which *N*-acetyl-*N′*-arylhydrazine **4** is treated with **3a** in PCl_3_ to give *N*-acetyl-*N*′-arylpyrazolone **7**, followed by hydrolysis to give the carboxylic acid **8** [29] (Scheme 1B). The second approach is one-step condensation of **4** with **3a** in the presence of a strong base, which deprotonates the more acidic amino group of **4** and makes it more reactive. Thus, the regioisomeric compounds **2** are obtained. However, despite the simplicity of the latter approach, the scope and the yields are usually only moderate [27] (Scheme 1B). Since the known methods for the synthesis of **2** are not optimal, there is a need for the development of improved syntheses of **2**. We concluded that perhaps a 4 + 1 approach via cyclization of a C–C–N–N-type 1,4-dinucleophile **10** with a C_1_-electrophile could provide an elegant route to obtaining compounds **2**. The arylhydrazines **4** are first acylated with methyl malonyl chloride (**9**) to give hydrazides **10**. Subsequent treatment of **10** with *tert*-butoxy-bis(dimethylamino)methane (TBDMAM, Bredereck’s reagent) leads to the corresponding enaminone intermediates **11**, which cyclize to the desired 3-pyrazolone-4-carboxylates **2** (Scheme 1C).

Continuing our studies on the development of synthetic methods for the preparation of pyrazole derivatives and their evaluation as potential inhibitors of *Pf*DHODH, we focused on the synthesis of pyrazolone esters **1** and **2** as potential lead structures. In the following, we report the results of this study, including the synthesis of 1-aryl-5-hydroxy-1*H*-pyrazole-4-carboxylates **1a**–**d**, the development of a new synthetic method, the preparation of isomeric 1-aryl-3-hydroxy-1*H*-pyrazole-4-carboxylates **2a**–**d**, and the evaluation of products **1a**–**d** and **2a**–**d** for the inhibition of *Pf*DHODH.

## 2. Results and Discussion

### 2.1. In Silico Evaluation of Compounds ***3a***, ***1a***–***d***, ***2a***–***d***, ***5a***–***d***, and ***10a***–***d***

**Figure 3:**
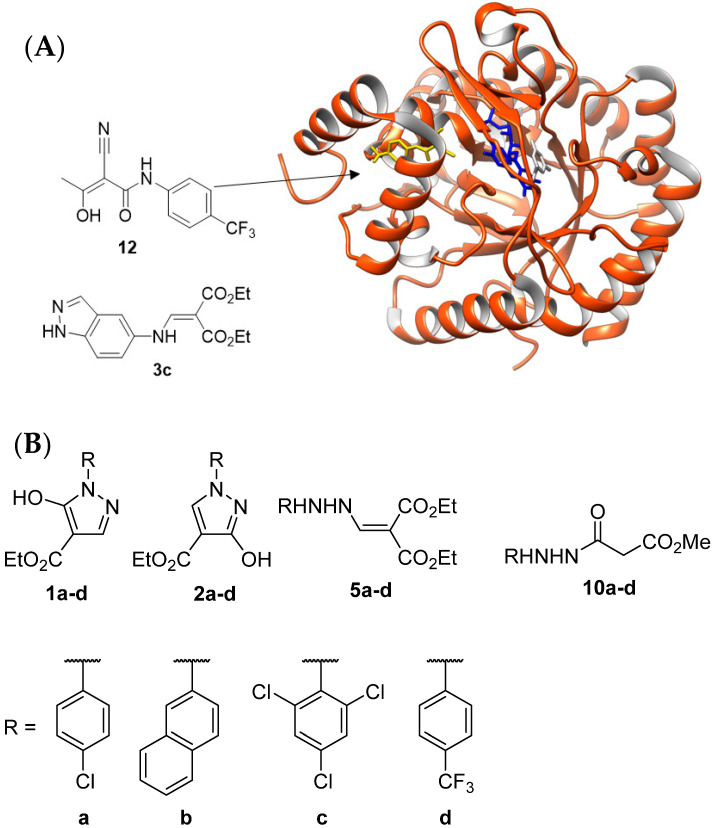
(**A**): Three-dimensional structure of *Pf*DHODH (PDB accession code 1TV5) highlighting positions of the binding sites in the active site. The protein is shown as ribbons, the prosthetic group FMN is shown as blue sticks, orotate is shown as gray sticks, and the known inhibitor Teriflunomide (**12**), bound to the quinone binding site, is shown as yellow sticks. (**B**): The structures of the in silico investigated compounds **1a**–**d**, **2a**–**d**, **5a**–**d**, and **10a**–**d**.

We began this study with the in silico evaluation of compounds with general structures **1** and **2** (cf. Figure 2) as possible inhibitors of *Pf*DHODH. These compounds were subjected to molecular docking into the active site of *Pf*DHODH [30,31] (Figure 3A). The active center contains the FMN prosthetic group and binding sites for dihydroorotate and the ubiquinone cofactor. The latter is shaped as a hydrophobic tunnel. It is much larger than the dihydroorotate binding site and exhibits comparatively higher interspecies variability, allowing for the higher selectivity of inhibitors for *Pf*DHODH over *Hs*DHODH. Therefore, this is the usual target site of choice for inhibitor development, for example, 1TV5 [31]. The dihydroorotate binding site has a suitable size for compounds such as the known inhibitors (2*Z*)-2-cyano-3-hydroxy-*N*-[4-(trifluoromethyl)phenyl]but-2-enamide (Teriflunomide) (**12**) [30,31] and diethyl 2-{([1*H*-indazol-5-yl)amino]methylidene}malonate (**3c**) [12]. Accordingly, this binding site could also harbor the conformationally constrained pyrazolone analogues **1** and **2** and their precursors **5** and **10** (Figure 3B, cf. Figure 2), which also follow Lipinski’s rule of five [32,33,34]. Four characteristic aryl residues, 4-chlorophenyl (**a**), 2-naphthyl (**b**), 2,4,6-trichlorophenyl (**c**), and 4-trifluoromethylphenyl (**d**), were selected for the design of the target compounds **1a**–**d** and **2a**–**d** and their precursors (intermediates) **5a**–**d** and **10a**–**d** (Figure 3B). The selection of substituents **a**–**d** was based on the assumption that the *N*-aryl residues must be *para*-substituted to ensure lipophilic interactions with the hydrophobic tunnel. In addition, we chose the 2-naphthyl residue **b** because it represents a larger aromatic system and the 2,4,6-trichlorophenyl residue **c** due to its larger steric bulk, halogen bonding, and orthogonal bonding potential [35,36,37]. Both structural differences could affect hydrogen, halogen, and orthogonal bonding potential and/or hydrophobic and π–π interactions. For comparison with compounds **1**, **2**, **5**, and **10**, we decided to also investigate the known inhibitors **3c** [12], **12** [31] (Figure 3B), **A**, **C**, and **D** [12,14,15] (cf. Figure 1) in this part of the study.

The binding affinities of compounds **1a**–**d**, **2a**–**d**, **5a**–**d**, and **10a**–**d** and known inhibitors **3c**, **12** (cf. Figure 3), **A**, **C**, and **D** (cf. Figure 1) to *Pf*DHODH were evaluated using Glide molecular docking software. The results are presented in the Supplementary Materials (Table S1), whereas the selected results for **1a**–**d**, **2a**–**d**, and known inhibitors **3a** and **12** are shown in Table 1. In general, the pyrazole derivatives **1** and **2** had better docking scores than their open-chain hydrazine precursors **5** and **10** (Table S1). As expected, the known inhibitor **12** had the best overall score of about –10.5, whereas the score of the known inhibitor **3c** was much lower at about –6.5 (Table 1, entries 9 and 10). Encouragingly, all synthesized compounds **1a**–**d** (Dscores –5.52 to –7.80) and **2a**–**d** (Dscores –6.85 to –8.39) performed better than the known inhibitor **3** (Dscores around –6.5) (Table 1, entries 1–9), with 3-hydroxypyrazole **2c** consistently achieving a Dscore of around –8 with both docking methods (Table 1, entry 7).

### 2.2. Synthesis of Compounds ***1a***–***d***, ***2a***–***d***, ***3c***, ***5a***–***d***, and ***10a***–***d***

**Scheme 2:**
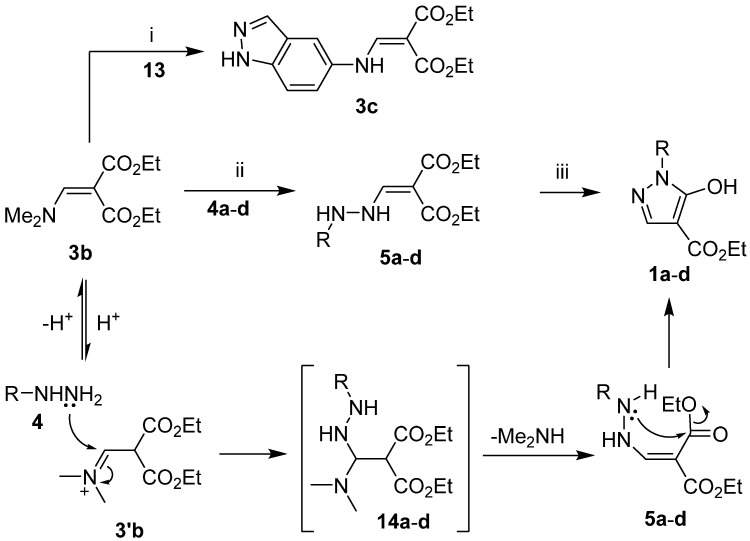
Synthesis of compounds **1a**–**d**, **3c**, and **5a**–**d**. Reaction conditions: (i) 1*H*-indazol-5-amine (**13**) (1 equiv.), 37% aq. HCl (1 equiv.), EtOH, r.t., 24 h; (ii) hydrazine **4a**–**d** hydrochloride (1 equiv.), EtOH, r.t., 24 h; (iii) H_2_O–EtOH–Et_3_N (3:3:1), reflux, 1.5 h.

Encouraged by the molecular docking results, we proceeded with the synthesis of target compounds **1**, **2**, and **3c**. First, the reference inhibitor **3c** and enhydrazines **5a**–**d** were prepared in 70–94% yields by acid-catalyzed transamination of diethyl 2-[(dimethylamino)methylene]malonate (**3b**) [38] with 5-amino-1*H*-indazole (**13**) and arylhydrazines **4a**–**d**, respectively. Subsequently, the enhydrazines **5a**–**d** were cyclized in a 3:3:1 mixture of water, methanol, and triethylamine according to the literature procedure for the synthesis of closely related alkyl 1-aryl-5-hydroxy-1*H*-pyrazole-4-carboxylates [28] to afford the desired ethyl 1-aryl-5-hydroxy-1*H*-pyrazole-4-carboxylates **1a**–**d** in 60–86% yields. The reaction pathway is explainable by the initial 1,4-addition of hydrazine **4** to the protonated enaminone **3′b**, giving adduct **14**, followed by the elimination of dimethylamine to give enhydrazine **5**. The base-catalyzed intramolecular cyclocondensation of **5** then yields the 5-hydroxypyrazole derivative **1** (Scheme 2, Table 2).

**Scheme 3:**
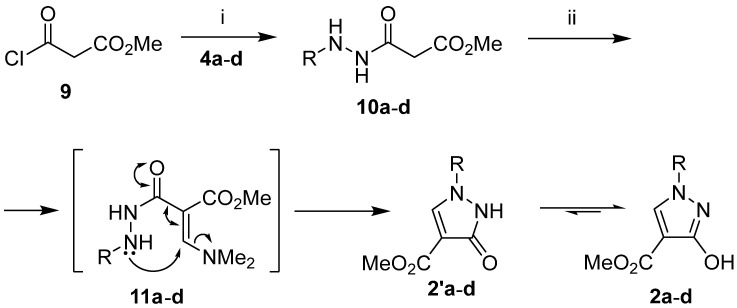
Synthesis of compounds **2a**–**d** and **10a**–**d**. Reaction conditions: (i) arylhydrazine **4a**–**d** (1 equiv.), CH_2_Cl_2_, Et_3_N (2 equiv.), 0 °C, 20 min., then r.t., 2 h; (ii) TBDMAM (1 equiv.), toluene, 40 °C, 30 min., then reflux, 2 h.

We then proceeded with the synthesis of the isomeric methyl 1-aryl-3-hydroxy-1*H*-pyrazole-4-carboxylates **2a**–**d** according to the proposed two-step synthetic method (cf. Scheme 1). The acylation of arylhydrazines **4a**–**d** with methyl 3-chloro-3-oxopropanoate (**9**) was carried out in anhydrous dichloromethane in the presence of one equivalent of triethylamine and gave the corresponding hydrazides **10a**–**d** in 47–53% yields. The anhydrous conditions were essential for the successful performance of the first step. Subsequent treatment of hydrazides **10a**–**d** with an equivalent amount of TBDMAM in anh. toluene at 40–110 °C gave the desired pyrazole derivatives **2a**–**d** in 48–74% yields. It is noteworthy that mixtures of products were formed, and the yield of **2** decreased significantly unless heating was carried out first at 40 °C for half an hour and then at 110 °C. This observation is consistent with the proposed cyclization reaction pathway, in which the first selective formation of the enaminone intermediate **11** occurs at a slightly elevated temperature. After increasing the temperature to 110 °C, cyclization of the anilino group to the enaminone residue occurs via a 1,4-addition–elimination process to form the pyrazolone **2′**, which tautomerizes to the desired 3-hydroxypyrazole derivative **2** (Scheme 3, Table 3).

### 2.3. Structure Determination

**Figure 4:**
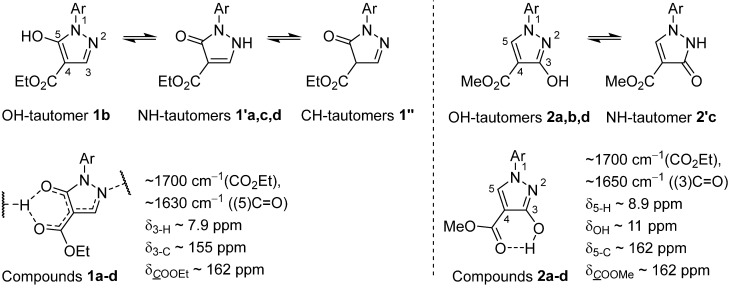
The structure and tautomerism of pyrazoles **1a**–**d** and **2a**–**d**.

The structures of compounds **1a**–**d**, **2a**–**d**, **3c**, **5a**–**d**, and **10a**–**d** were determined by spectroscopic methods (IR, ^1^H and ^13^C NMR, and MS-HRMS) and by elemental analyses for C, H, and N. The NMR data for compounds **1a**–**d**, **2a**–**d**, **3c**, **5a**–**d**, and **10a**–**d** are in agreement with the literature data for related compounds [12,28,38,39,40,41]. Tautomerism of compounds **1** and **2** in the solid state was determined by IR spectroscopy (Figure 4). The IR spectra of compounds **1** and **2** exhibited typical C=O absorption bands at about 1700 cm^−1^, corresponding to the α,β-unsaturated ester carbonyl group, while compounds **1a**,**c**,**d** and **2c** also exhibited less intense vibrations at about 1640 cm^−1^, corresponding to the lactam carbonyl group of NH-tautomers **1′a,c,d** and **2′c**, respectively. Typical aliphatic ester vibrations around 1740 cm^−1^ characteristic of the CH-tautomeric forms **1′′** were not observed. Accordingly, compounds **1b** and **2a,b,d** exist in the solid state as OH-tautomers, while compounds **1a,c,d** and **2c** exist in the solid state as the NH-tautomers **1′a,c,d** and **2′c**, respectively. Due to possible intra- and intermolecular hydrogen bonding, the **1a**–**d** and **2a**–**d** tautomers in the solid state may also be a hybrid of OH- and NH-tautomers, which has already been observed in related pyrazolone derivatives (Figure 4) [42,43,44]. In the ^1^H NMR spectra of compounds **2a**–**d** in DMSO-*d*_6_, the signal for the OH/NH proton appeared at about 11 ppm, while it was absent (exchanged) in the ^1^H NMR spectra of compounds **1a**–**d**. This observation is consistent with a faster proton exchange in compounds **1a**–**d**, which can be explained by stronger hydrogen bonding. The CH-tautomers **1′′** were not present in the ^1^H NMR spectra of compounds **1a**–**d**, as evidenced by the absence of signals for the methine proton H–C(4). In the ^1^H and ^13^C NMR spectra of 5-hydroxypyrazoles **1a**–**d**, the chemical shifts of the 3–H and 3–C nuclei were around 7.9 ppm and 155 ppm, respectively, whereas in the spectra of 3-hydroxypyrazoles **2a**–**d**, the chemical shifts of the 5–H and 5–C nuclei were around 8.9 ppm and 162 ppm, respectively (Figure 4). These spectral data are in agreement with the typical spectral data of the related isomeric pyrazoles **1** and **2** [26,42,45].

## 3. Inhibition of *Pf*DHODH

**Figure 5:**
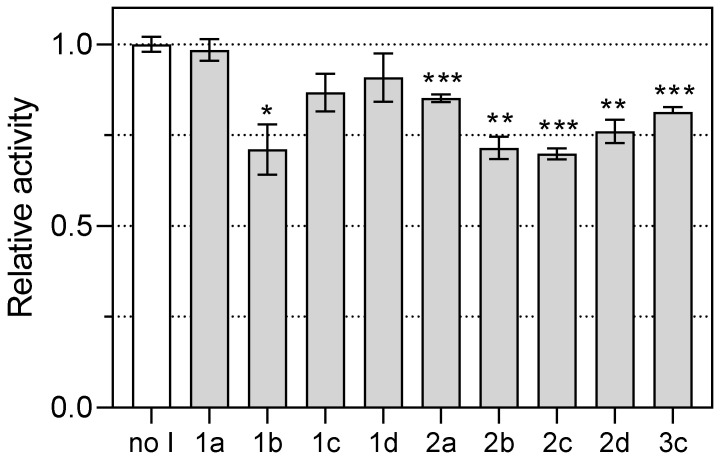
Effect of compounds **1a–d**, **2a–d**, and **3c** on *Pf*DHODH activity. Asterisks denote statistically significant pairwise differences between the samples and uninhibited control (* *p* < 0.05, ** *p* < 0.01, *** *p* < 0.001) calculated with Welch’s *t*-test. The plot was drawn with GraphPad Prism 9.4 Software (GraphPad Inc., San Diego, CA, USA), which was also used for statistical comparison of data.

The synthesized pyrazole derivatives **1a**–**d** and **2a**–**d** and the known inhibitor **3c** were tested for their inhibition of *Pf*DHODH. A colorimetric assay measuring the reduction of the redox dye 2,6-dichlorophenolindophenol (DCIP) coupled with the re-oxidation of the coenzyme was used. The results are shown in Figure 5 and Table 4. Compounds **1b** and **2a**–**d** showed weak inhibitory activity at a concentration of 50 μM. Their relative potencies were calculated as a percentage of inhibition compared to the uninhibited control (Table 4). All compounds studied were at best weak inhibitors of *Pf*DHODH. The strongest potency was found for compounds **1b** and **2b**–**d** with approx. 30% inhibition of *Pf*DHODH activity (Table 4, entries 2, 6–8). Compound **2a** showed about 15% inhibition (Table 4, entry 5), while compounds **1a**, **1c**, and **1d** did not show statistically significant inhibition of PfDHODH activity (Table 4, entries 1, 3, and 4). However, our assays also showed only weak activity of the known inhibitor **3c** (19% inhibition; Table 4, entry 9). Thus, most of the synthesized compounds were equally active (**2a**) or even more active (**1b** and **2b**–**d**) than the known inhibitor **3c** (Table 4).

**Figure 6:**
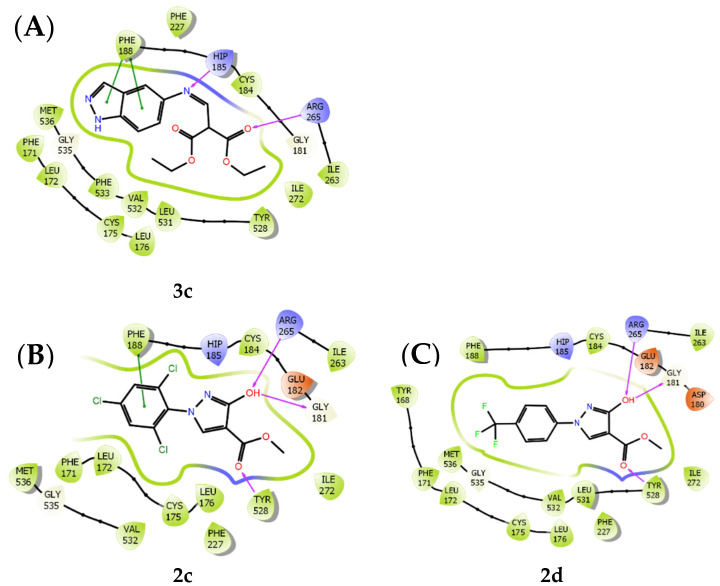
Molecular docking of compounds **3c** (**A**), **2c** (**B**), and **2d** (**C**) into the coenzyme-binding site of *Pf*DHODH.

Since the docking results showed quite solid complementarity between the binding site and the designed ligands (cf. Table 1, Section 2.1.), we expected encouraging results in the in vitro assays, but unfortunately, all ligands proved to be weakly bound to the receptor position. Ligand **2c** had the best affinity for *Pf*DHODH, with 30% inhibition at a concentration of 50 μM of the inhibitor (cf. Table 4, entry 7). The proposed docking of the selected compounds **3c**, **2c**, and **2d** to the quinone binding site of the active site of *Pf*DHODH is shown in Figure 6. The known inhibitor **3c** shows hydrogen bonding interactions between the imine nitrogen and the histidine residue HIP185 and between the ester carbonyl oxygen and the arginine residue ARG265, as well as a π–π interaction between the indazole residue and the phenyl ring of the phenylalanine residue PHE188 (Figure 5A). In addition to the π–π interaction and hydrogen bonding of the ester group to the tyrosine residue TYR528, compounds **2c** and **2d** also exhibited hydrogen bonding of the 3-hydroxy group with two amino acid residues of *Pf*DHODH, ARG265 and GLY181 (Figure 5B,C).

## 4. Experimental Section

### 4.1. General Methods

All solvents and reagents were used as received. Melting points were determined using the SRS OptiMelt MPA100—Automated Melting Point System (Stanford Research Systems, Sunnyvale, CA, USA). The ^1^H NMR and ^13^C NMR spectra were recorded in CDCl_3_ and DMSO-*d*_6_ as solvents using Me_4_Si as the internal standard on a Bruker Avance DPX 300 and Bruker Avance III UltraShield 500 plus instrument (Bruker, Billerica, MA, USA) at 300 and 500 MHz for ^1^ H and at 75.5 and 126 MHz for the ^13^ C nucleus, respectively. IR spectra were recorded on a Bruker FTIR Alpha Platinum spectrophotometer (Bruker, Billerica, MA, USA). Microanalyses were performed by combustion analysis on a Perkin-Elmer CHN Analyzer 2400 II (PerkinElmer, Waltham, MA, USA). Mass spectra were recorded on an Agilent 6224 Accurate Mass TOF LC/MS (Agilent Technologies, Santa Clara, CA, USA). Column chromatography was performed on silica gel (Silica gel 60, particle size: 0.035–0.070 mm, Sigma-Aldrich, St. Louis, MO, USA).

1*H*-Indazol-5-amine (**13**), hydrazines **4a**–**d**, methyl 3-chloro-3-oxopropanoate (**9**), and *tert*-butoxy-bis(dimethylamino)methane (TBDMAM, *Bredereck’s* reagent) are commercially available (Sigma-Aldrich). Compound **3b** was prepared following the literature procedure [38].

Molecular docking was performed with Glide, a part of the Schrödinger suite software [46]. The crystal structure of PfDHODH retrieved from the Protein Data Bank under accession code 1TV5 was used as the receptor molecule [31]. The docking protocol consisted of four phases. In the first phase, Schrödinger’s Protein Preparation Wizard (PPW) [47] was used to prepare the protein. The preparation of the protein using PPW is a semi-automatic process including several steps. In the first step, the protein structure (PDB-ID: 1TV5) was downloaded from the Protein Data Bank (PDB) and placed into the workspace of the Maestro GUI. Then, PPW was used to correct bond orders of the ligand and cofactor. Then, hydrogens were applied to all atoms of the model. This step was followed by the neutralization of side chains, which are not close to the binding site and do not participate in salt bridges, and finally, by the restraint minimization of the protein structure. In the next phase, the Receptor Grid Generator generated the interaction grid. Finally, the ligand library was prepared using the original SDF representation of the ligands with LigPrep. LigPrep is a Schrödinger’s utility that combines tools for generating 3D structures from linear SMILES code or 2D SDF. In our case, Schrödinger’s Glide in XP (extra precision) mode was used to perform molecular docking [48].

### 4.2. Biochemistry

#### 4.2.1. Expression and Purification of Recombinant DHODH

The expression plasmid pRSETb was a kind gift from Prof. Jon Clardy (Harvard Medical School, Boston, MA, USA). The plasmid contains a codon-optimized sequence for an N-truncated variant of *Pf*DHODH (amino acids 159–565). The sequence was further optimized using overlap extension PCR to remove a segment encoding amino acids 384–413, which form a disordered loop, and the product was subcloned into a second expression plasmid, pET28, in-frame with a C-terminal His_6_-tag. This construct was transformed into *E. coli* strain BL21 (DE3), and the cells were then grown at 37 °C to an OD_600_ value of approx. 0.8. Expression of the recombinant protein was induced via autoinduction, and cells were grown overnight at 16 °C. Cells were then collected by centrifugation, resuspended in Ni-affinity chromatography binding buffer (20 mM HEPES, pH 7.5, 500 mM NaCl, and 20 mM imidazole), and lysed by sonication. The resulting homogenate was cleared by centrifugation, and the supernatant was filtered and applied to a HisTrap FF 1 mL column (Cytiva). The column was washed with 10 column volumes of binding buffer, and bound proteins were then eluted with elution buffer (20 mM HEPES, pH 7.5, 500 mM NaCl, and 500 mM imidazole). Fractions containing eluted proteins (approx. sample volumes 500 μL) were pooled, diluted in cation exchange binding buffer (50 mM phosphate, pH 6.7), and applied to a HiTrap SP HP 1 mL column (Cytiva), which was then washed with 10 column volumes of binding buffer. The remaining proteins were eluted in a 20 min linear gradient of 0–100% with elution buffer (50 mM phosphate, pH 6.7, and 1 M NaCl). Fractions containing the desired protein were then pooled, concentrated to a volume of 500 μL, and injected onto a Superdex 75 10/300 GL column to be purified by size exclusion chromatography using the appropriate buffer (20 mM HEPES, pH 7.4, and 500 mM NaCl). The resulting fractions were concentrated and stored at −80 °C until use.

#### 4.2.2. Enzyme Assays

*Pf*DHODH activity was determined with a colorimetric assay measuring the reduction of the redox dye 2,6-dichlorophenolindophenol (DCIP) at 600 nm coupled with the re-oxidation of the coenzyme decylubiquinone [49]. Assays were performed in 200 μL reaction mixtures in 96-well microtiter plates containing 200 μM L-dihydroorotic acid, 20 μM decylubiquinone, 120 μM DCIP, ~50 nM enzyme, and each of the tested compounds at 50 μM. All compounds were dissolved in DMSO. The total concentration of DMSO in the reaction mixtures did not exceed 5% (*v*/*v*). Reaction mixtures were incubated for 25 min. at 25 ± 1 °C, and the absorption of the samples was then read at 600 nm in a Tecan infinite 200 Pro spectrophotometer. Appropriate controls without enzyme were performed in parallel to account for background reduction of DCIP, along with positive controls containing the enzyme but with none of the tested compounds. The percentage of inhibition was determined as:

$$\%\;\mathrm{inhibition}\;=\frac{A_{\mathrm{blank}}\;-{\;A}_{i}}{A_{\mathrm{blank}\;}-{\;A}_{z}}$$

where A_i_ and A_z_ are absorption values of reactions in the presence and absence of an inhibitor, and A_blank_ is the absorption value of the appropriate negative control without the enzyme. All experiments were performed in three parallels.

### 4.3. General Procedure for the Synthesis of Enamine ***3c*** and Enhydrazines ***5a***–***d***

A mixture of enaminone **3b** (1 mmol), amine **13** or hydrazine **4** (1 mmol), ethanol (2 mL), and 37% aq. HCl (0.1 mL ~3 drops, ~1 mmol) was stirred at room temperature for 24 h. The addition of 37% aq. HCl was omitted when the hydrochloride salt of amine **13** or hydrazine **4** was used. Water (3 mL) was added, and stirring at room temperature was continued for 30 min. The precipitate was collected by filtration, washed with water (3 mL), and dried in vacuo over NaOH pellets to give **3c** or **5**. The following compounds were prepared in this manner:

#### 4.3.1. Diethyl 2-{[(1*H*-Indazol-5-yl)amino]methylene}malonate (**3c**)

Prepared from **1** (215 mg, 1 mmol) and 5-amino-1*H*-indazole (**13**) (133 mg, 1 mmol). Yield: 250 mg (83%) of light pink solid; mp 164–167 °C, [12] mp 167–169 °C (found: C, 58.99; H, 5.42; N, 14.11. C_15_H_17_N_3_O_4_ requires C, 59.40; H, 5.65; N, 13.85%); δ_H_ (300 MHz, DMSO-*d*_6_) 1.24 and 1.27 (6H, 2t, 1:1, *J* = 7.2 Hz), 4.13 (2H, q, *J* = 7.1 Hz), 4.21 (q, *J* = 7.1 Hz, 2H), 7.40 (dd, *J* = 8.9, 2.2 Hz, 1H), 7.57 (d, *J* = 8.9 Hz, 1H), 7.73 (d, *J* = 2.1 Hz, 1H), 8.06 (s, 1H), 8.43 (d, *J* = 14.0 Hz, 1H), 10.83 (d, *J* = 14.0 Hz, 1H), and 13.11 (s, 1H); HRMS (ESI): MH^+^, found 304.1298. [C_15_H_18_N_3_O_4_]^+^ requires 304.1292. Spectral data are in agreement with the literature data [12].

#### 4.3.2. Diethyl 2-{[2-(4-Chlorophenyl)hydrazinyl]methylene}malonate (**5a**)

Prepared from **3b** (215 mg, 1 mmol) and 4-chlorophenylhydrazine hydrochloride (**4a**) (176 mg, 1 mmol). Yield: 220 mg (70%) of yellow solid; mp 115–116 °C, [39] mp 116 °C (found: C, 54.07; H, 5.25; N, 8.68. C_14_H_17_ClN_2_O_4_ requires C, 53.77; H, 5.48; N, 8.96%); ν_max_ (ATR) 3264, 2977, 1674, 1645, 1610, 1492, 1427, 1273, 1246, 1222, 1075, 821, and 688 cm^−1^; δ_H_ (500 MHz, DMSO-*d*_6_) 1.17 (t, *J* = 7.1 Hz, 3H), 1.22 (t, *J* = 7.1 Hz, 3H), 4.04 (q, *J* = 7.1 Hz, 2H), 4.15 (q, *J* = 7.0 Hz, 2H), 6.71 (d, *J* = 8.8 Hz, 2H), 7.25 (s, 2H), 7.92 (d, *J* = 11.9 Hz, 1H), 8.66 (s, 1H), and 10.07 (d, *J* = 12.0 Hz, 1H); δ_C_ (126 MHz, DMSO-*d*_6_) 14.27, 14.33, 59.08, 59.18, 89.04, 114.25, 123.46, 128.84, 147.56, 160.51, 164.62, and 166.61; HRMS (ESI): MH^+^, found 313.0946. [C_14_H_18_^35^ClN_2_O_4_]^+^ requires 313.095.

#### 4.3.3. Diethyl 2-{[2-(Naphthalene-2-yl)hydrazinyl]methylene}malonate (**5b**)

Prepared from **3b** (215 mg, 1 mmol) and 2-naphthylhydrazine hydrochloride (**4b**) (195 mg, 1 mmol). Yield: 282 mg (86%) of orange solid; mp 86–87 °C (found: C, 65.67; H, 6.06; N, 8.46. C_18_H_20_N_2_O_4_ requires C, 65.84; H, 6.14; N, 8.53%); ν_max_ (ATR) 3284, 2975, 1678, 1635, 1600, 1425, 1279, 1226, 1213, 1186, 1077, 1038, 840, 791, 736, and 694 cm^−1^; δ_H_ (500 MHz, DMSO-*d*_6_) 1.19 (t, *J* = 7.1 Hz, 3H), 1.26 (t, *J* = 7.0 Hz, 3H), 4.06 (q, *J* = 7.1 Hz, 2H), 4.19 (q, *J* = 7.1 Hz, 2H), 6.98 (d, *J* = 2.1 Hz, 1H), 7.05 (dd, *J* = 8.9, 2.3 Hz, 1H), 7.27 (t, *J* = 8.0, 6.9, 1.2 Hz, 1H), 7.40 (t, *J* = 8.1, 6.8, 1.3 Hz, 1H), 7.70–7.82 (m, 3H), 8.03 (d, *J* = 12.0 Hz, 1H), 8.79 (s, 1H), and 10.19 (d, *J* = 12.0 Hz, 1H); δ_C_ (126 MHz, DMSO-*d*_6_) 14.30, 14.36, 59.08, 59.21, 89.01, 105.83, 115.95, 123.06, 126.29, 126.50, 127.55, 128.43, 129.00, 134.03, 146.35, 160.64, 164.67, and 166.77; HRMS (ESI): MH^+^, found 329.1496. [C_18_H_21_N_2_O_4_]^+^ requires 329.1496.

#### 4.3.4. Diethyl 2-{[2-(2,4,6-Trichlorophenyl)hydrazinyl]methylene}malonate (**5c**)

Prepared from **3b** (215 mg, 1 mmol) and 2,4,6-trichlorophenylhydrazine hydrochloride (**4c**) (211 mg, 1 mmol). Yield: 300 mg (79%) of white solid; mp 90–92 °C, [40] mp 92 °C (found: C, 44.07; H, 3.85; N, 7.20. C_14_H_25_Cl_3_N_2_O_4_ requires C, 44.06; H, 3.96; N, 7.34%); ν_max_ (ATR) 3300, 2973, 1688, 1633, 1419, 1379, 1262, 1226, 1059, 1032, 882, 793, 689, and 640 cm^−1^; δ_H_ (500 MHz, DMSO-*d*_6_) 1.20 (6H, t, *J* = 7.1 Hz), 4.06 (2H, q, *J* =, 7.1 Hz), 4.11 (2H, q, *J* =, 7.1 Hz), 7.61 (2H, s), 8.12 (1H, d, *J* = 11.9 Hz), 8.25 (1H, s), and 10.11 (1H, d, *J* = 11.9 Hz); δ_C_ (126 MHz, DMSO-*d*_6_) 14.28, 59.07, 59.11, 88.51, 126.61, 127.37, 128.74, 139.08, 160.42, 164.75, and 166.58; HRMS (ESI): MH^+^, found 381.017. [C_14_H_16_^35^Cl_3_N_2_O_4_]^+^ requires 381.017.

#### 4.3.5. Diethyl 2-{[2-(4-Trifluoromethylphenyl)hydrazinyl]methylene}malonate (**5d**)

Prepared from **1** (215 mg, 1 mmol) and 4-trifluoromethylphenylhydrazine hydrochloride (**4d**) (176 mg, 1 mmol). Yield: 324 mg (94%) of yellowish solid; mp 126–127 °C (found: C, 51.88; H, 4.81; N, 8.01. C_15_H_17_F_3_N_2_O_4_ requires C, 52.02; H, 4.95; N, 8.09%); ν_max_ (ATR) 3260, 2985, 1674, 1635, 1616, 1424, 1326, 1275, 1253, 1222, 1157, 1098, 1064, 1033, 1010, 834, 792, and 694 cm^−1^; δ_H_ (500 MHz, DMSO-*d*_6_) 1.18 (t, *J* = 7.1 Hz, 3H), 1.23 (t, *J* = 7.1 Hz, 3H), 4.04 (q, *J* = 7.1 Hz, 2H), 4.16 (q, *J* = 7.1 Hz, 2H), 6.82 (d, *J* = 8.4 Hz, 2H), 7.55 (d, *J* = 8.5 Hz, 2H), 7.91 (d, *J* = 12.0 Hz, 1H), 9.09 (s, 1H), and 10.14 (d, *J* = 12.0 Hz, 1H); δ_C_ (126 MHz, DMSO-*d*_6_) 14.75, 14.81, 59.62, 59.72, 90.07, 112.62, 120.18 (q), 125.29 (q), 126.93, 152.29, 160.86, 165.06, and 166.94; HRMS (ESI): MH^+^, found 347.1214. [C_15_H_18_F_3_N_2_O_4_]^+^ requires 347.1213.

### 4.4. General Procedure for the Synthesis of 1-Aryl-5-hydroxy-1H-pyrazole-4-carboxylates ***1a***–***d***

Compounds **1a**–**d** were obtained following the literature procedure for the preparation of closely related compounds [28]. A mixture of enhydrazine **5** (0.5 mmol) and H_2_O–MeOH–Et_3_N (3:3:1, 5 mL) was stirred under reflux for 1.5 h. Volatile components were evaporated in vacuo, and the residue was triturated with 10% aq. HCl (4 mL). The precipitate was collected by filtration, washed with water (2 mL), and triturated again with a mixture of ethanol and water (1:1, 2 mL). The precipitate was collected by filtration and dried in vacuo over NaOH pellets to give **1**. The following compounds were prepared in this manner:

#### 4.4.1. Ethyl 1-(4-Chlorophenyl)-5-hydroxy-1*H*-pyrazole-4-carboxylate (**1a**)

Prepared from **5a** (156 mg, 0.5 mmol). Yield: 80 mg (60%) of white solid; mp 147–149 °C, [39] mp 149 °C (found: C, 54.19; H, 4.00; N, 10.09. C_12_H_11_ClN_2_O_3_ requires C, 54.05; H, 4.16; N, 10.50%); ν_max_ (ATR) 2987, 1711, 1524, 1495, 1402, 1343, 1241, 1198, 1077, 818, 774, and 732; δ_H_ (500 MHz, DMSO-*d*_6_) 1.27 (t, *J* = 7.1 Hz, 3H), 4.22 (q, *J* = 7.1 Hz, 2H), 7.56 (d, 2H), 7.74 (d, 2H), and 7.83 (s, 1H), OH exchanged; δ_C_ (126 MHz, DMSO-*d*_6_) 14.40, 59.23, 96.40, 123.53, 129.03, 130.96, 136.57, 140.57, 154.66, and 162.28; HRMS (ESI): MH^+^, found 267.0527. [C_12_H_12_^35^ClN_2_O_3_]^+^ requires 267.0531.

#### 4.4.2. Ethyl 1-(Naphthalen-2-yl)-5-hydroxy-1*H*-pyrazole-4-carboxylate (**1b**)

Prepared from **5b** (164 mg, 0.5 mmol). Yield: 110 mg (78%) of white solid; mp 149–151 °C (found: C, 67.90; H, 4.88; N, 9.48. C_16_H_14_N_2_O_3_ requires C, 68.08; H, 5.00; N, 9.92%); ν_max_ (ATR) 3275, 2980, 1681, 1570, 1535, 1416, 1313, 1277, 1134, 1101, 975, 937, 894, 850, 818, 780, 745, and 636; δ_H_ (500 MHz, DMSO-*d*_6_) 1.29 (t, *J* = 7.1 Hz, 3H), 4.25 (q, *J* = 7.1 Hz, 2H), 7.517.63 (m, 2H), 7.86–7.89 (m, 2H), 7.96–8.02 (m, 2H), 8.05 (d, *J* = 8.9 Hz, 1H), and 8.23 (d, *J* = 2.1 Hz, 1H), OH exchanged; δ_C_ (126 MHz, DMSO-*d*_6_) 14.40, 59.22, 96.41, 119.91, 121.06, 126.36, 126.89, 127.61, 128.03, 128.77, 131.36, 132.69, 135.15, 140.42, 154.62, and 162.36; HRMS (ESI): MH^+^, found 283.1075. [C_16_H_15_N_2_O_3_]^+^ requires 283.1077.

#### 4.4.3. Ethyl 1-(2,4,6-Trichlorophenyl)-5-hydroxy-1*H*-pyrazole-4-carboxylate (**1c**)

Prepared from **5c** (191 mg, 0.5 mmol). Yield: 145 mg (86%) of white solid; mp 223–225 °C, [40] mp 228 °C (found: C, 42.66; H, 2.28; N, 8.31. C_12_H_9_Cl_3_N_2_O_3_ requires C, 42.95; H, 2.70; N, 8.35%); ν_max_ (ATR) 3067, 1691, 1638, 1548, 1461, 1400, 1375, 1341, 1262, 1177, 1148, 1042, 924, 863, 804, 788, 753, and 657; δ_H_ (500 MHz, DMSO-*d*_6_) 1.27 (t, *J* = 7.1 Hz, 3H), 4.21 (q, *J* = 7.1 Hz, 2H), 7.88 (s, 1H), and 7.96 (s, 2H), OH exchanged; δ_C_ (126 MHz, DMSO-*d*_6_) 14.90, 59.70, 95.43, 129.33, 132.19, 135.62, 136.38, 142.27, 156.18, and 162.60; HRMS (ESI): MH^+^, found 334.9746. [C_12_H_10_^35^Cl_3_N_2_O_3_]^+^ requires 334.9752.

#### 4.4.4. Ethyl 1-(4-Trifluoromethylphenyl)-5-hydroxy-1*H*-pyrazole-4-carboxylate (**1d**)

Prepared from **5d** (173 mg, 0.5 mmol). Yield: 120 mg (80%) of white solid; mp 174–177 °C, [41] mp not given (found: C, 51.86; H, 3.47; N, 9.28. C_13_H_11_F_3_N_2_O_3_ requires C, 52.01; H, 3.69; N, 9.33%); ν_max_ (ATR) 3164, 2903, 2752, 1708, 1628, 1560, 1333, 1191, 1168, 1119, 1099, 1059, 927, 828, 786, 752, and 704; δ_H_ (500 MHz, DMSO-*d*_6_) 1.28 (t, *J* = 7.1 Hz, 3H), 4.23 (q, *J* = 7.1 Hz, 2H), 7.88 (d, *J* = 9.3 Hz, 3H), and 7.99 (d, *J* = 8.4 Hz, 2H), OH exchanged; δ_C_ (126 MHz, DMSO-*d*_6_) 14.37, 59.29, 96.68, 121.76, 124.03 (q), 126.31 (d), 126.72 (q), 140.92, 141.14, 155.26, and 162.21; HRMS (ESI): MH^+^, found 301.0797. [C_13_H_12_F_3_N_2_O_3_]^+^ requires 301.0795.

### 4.5. General Procedure for the Synthesis of Hydrazides ***10a***–***d***

Under argon, a solution of methyl 3-chloro-3-oxopropanoate (**9**) (325 μL, 3.0 mmol, 1.15 equiv.) in anh. CH_2_Cl_2_ (12 mL) was added slowly at 0 °C (ice-bath) to a stirred mixture of hydrazine **4** hydrochloride (2.6 mmol), anh. CH_2_Cl_2_ (12 mL), and Et_3_N (1.1 mL, 7.8 mmol, 3 equiv.). Only 2 equiv. of Et_3_N (730 μL, 5.2 mmol) were added when free hydrazine **4** was used. Upon addition of **7**, the mixture was stirred at 0 °C for 20 min. and then at room temperature for 2 h. The reaction mixture was transferred into a separatory funnel and washed with water (2 × 10 mL) and brine (2 × 10 mL). The organic phase was dried over anh. Na_2_SO_4_ and filtered, the filtrate was evaporated in vacuo, and the residue was purified by CC (EtOAc). Fractions containing the product were combined and evaporated in vacuo to give **10**. The following compounds were prepared in this manner:

#### 4.5.1. Methyl 3-[2-(4-Chlorophenyl)hydrazinyl]-3-oxopropanoate (**10a**)

Prepared from **9** (370 μL, 3.34 mmol), hydrazine **4a** hydrochloride (528 mg, 2.9 mmol), and Et_3_N (1.25 mL, 8.7 mmol). Yield: 330 mg (47%) of white solid; mp 143–145 °C (found: C, 49.53; H, 4.59; N, 11.62. C_10_H_11_ClN_2_O_3_ requires C, 49.50; H, 4.57; N, 11.54%); ν_max_ (ATR) 3320, 2952, 1729, 1645, 1597, 1494, 1432, 1352, 1203, 1173, 1088, 1007, 971, 823, and 693; δ_H_ (300 MHz, DMSO-*d*_6_) 3.34 (s, 2H), 3.65 (s, 3H), 6.73 (d, *J* = 8.9 Hz, 1H), 7.17 (d, *J* = 8.9 Hz, 1H), 8.00 (d, *J* = 2.5 Hz, 1H), and 9.88 (d, *J* = 2.5 Hz, 1H); δ_C_ (126 MHz, DMSO-*d*_6_) 40.6, 52.0, 113.6, 121.8, 128.5, 147.9, 165.1, and 168.1; HRMS (ESI): MH^+^, found 243.0536. [C_10_H_12_^35^ClN_2_O_3_]^+^ requires 243.0531.

#### 4.5.2. Methyl 3-[2-(Naphthalene-2-yl)hydrazinyl]-3-oxopropanoate (**10b**)

Prepared from **9** (325 μL, 3 mmol), hydrazine **4b** hydrochloride (500 mg, 2.6 mmol), and Et_3_N (1.12 mL, 7.8 mmol). Yield: 320 mg (48%) of pinkish solid; mp 142–146 °C (found: C, 64.51; H, 5.34; N, 10.66. C_14_H_14_N_2_O_3_ requires C, 65.11; H, 5.46; N, 10.85%); ν_max_ (ATR) 3289, 3203, 2953, 1735, 1694, 1659, 1633, 1508, 1413, 1342, 1202, 1153, 1014, 820, 743, and 675; δ_H_ (500 MHz, DMSO-*d*_6_) 3.42 (s, 2H), 3.69 (s, 3H), 7.00 (d, *J* = 2.0 Hz, 1H), 7.08 (dd, *J* = 8.8, 2.3 Hz, 1H), 7.19–7.23 (m, 1H), 7.33–7.40 (m, 1H), 7.61 (d, *J* = 8.2 Hz, 1H), 7.69–7.74 (m, 2H), 8.15 (d, *J* = 2.5 Hz, 1H), and 10.00 (d, *J* = 2.5 Hz, 1H); δ_C_ (126 MHz, DMSO-*d*_6_) 52.0, 104.8, 116.0, 122.2, 125.8, 126.2, 127.5, 127.8, 128.4, 134.3, 146.7, 165.1, 168.1, and 170.8; HRMS (ESI): MH^+^, found 259.1078. [C_14_H_15_N_2_O_3_]^+^ requires 259.1077.

#### 4.5.3. Methyl 3-[2-(2,4,6-Trichlorophenyl)hydrazinyl]-3-oxopropanoate (**10c**)

Prepared from **9** (165 μL, 1.47 mmol), hydrazine **4c** (270 mg, 1.28 mmol), and Et_3_N (360 μL, 2.56 mmol). Yield: 210 mg (53%) of white solid; mp 172–174 °C (found: C, 38.36; H, 2.62; N, 8.86. C_10_H_9_Cl_3_N_2_O_3_ requires C, 38.80; H, 2.28; N, 9.05%); ν_max_ (ATR) 3307, 3201, 3016, 1741, 1663, 1552, 1434, 1288, 1150, 1006, 977, 927, 852, 721, and 610; δ_H_ (300 MHz, DMSO-*d*_6_) 3.25 (s, 2H), 3.59 (s, 3H), 7.35 (s, 1H), 7.49 (s, 2H), and 10.19 (s, 1H); δ_C_ (126 MHz, DMSO-*d*_6_) 52.3, 125.0, 125.7, 128.9, 129.4, 141.1, 165.1, and 168.0; HRMS (ESI): MH^+^, found 310.9755. [C_10_H_10_^35^Cl_3_N_2_O_3_]^+^ requires 310.9752.

#### 4.5.4. Methyl 3-[2-(4-Trifluoromethylphenyl)hydrazinyl]-3-oxopropanoate (**10d**)

Prepared from **9** (165 μL, 1.47 mmol), hydrazine **4d** (225 mg, 2.84 mmol), and Et_3_N (360 μL, 2.56 mmol). Yield: 370 mg (47%) of white solid; mp 136–137 °C (found: C, 47.61; H, 3.87; N, 10.11. C_11_H_11_F_3_N_2_O_3_ requires C, 47.49; H, 4.01; N, 10.07%); ν_max_ (ATR) 3342, 1731, 1650, 1615, 1320, 1157, 1094, 1063, 833, and 700; δ_H_ (500 MHz, DMSO-*d*_6_) 3.37 (s, 2H), 3.66 (s, 3H), 6.84 (d, *J* = 8.4 Hz, 2H), 7.47 (d, *J* = 8.5 Hz, 2H), 8.50 (d, *J* = 1.6 Hz, 1H), 10.04 (d, *J* = 1.7 Hz, 1H); δ_C_ (126 MHz, DMSO-*d*_6_) 52.0, 111.4, 118.3 (q, *J* = 31.7 Hz), 125.1 (q, *J* = 269.2 Hz), 126.2 (d, *J* = 4.2 Hz), 152.1, 165.2, 168.0, and 170.8; HRMS (ESI): MH^+^, found 277.0751. [C_11_H_12_F_3_N_2_O_3_]^+^ requires 277.0755.

### 4.6. General Procedure for the Synthesis of Methyl 1-Aryl-3-hydroxy-1H-pyrazole-4-carboxylates ***2a***–***d***

Under argon, TBDMAM (232 μL, 1.125 mmol, 1.5 equiv.) was added to a mixture of hydrazide **10** (0.75 mmol) and anh. toluene (5 mL), and the mixture was stirred at 40 °C for 30 min. and then under reflux for 2 h. Volatile components were evaporated in vacuo. The residue was triturated with methanol (2 mL) for 10 min., and the precipitate was collected by filtration and washed with water (2 mL) to give **2**. The following compounds were prepared in this manner:

#### 4.6.1. Methyl 1-(4-Chlorophenyl)-3-hydroxy-1*H*-pyrazole-4-carboxylate (**2a**)

Prepared from **10a** (182 mg, 0.75 mmol), TBDMAM (232 μL, 1.125 mmol), and anh. toluene (5 mL). Yield: 140 mg (74%) of pinkish solid; mp 206–208 °C (found: C, 52.41; H, 3.72; N, 10.99. C_11_H_9_ClN_2_O_3_ requires C, 52.29; H, 3.59; N, 11.09%); ν_max_ (ATR) 3119, 2948, 2722, 2626, 1691, 1606, 1587, 1542, 1504, 1452, 1431, 1320, 1294, 1252, 1229, 1188, 1124, 1096, 1057, 1011, 952, 891, 823, and 761; δ_H_ (300 MHz, DMSO-*d*_6_) 3.74 (s, 3H), 7.53 (ddd, *J* = 9.0, 3.1, 2.2 Hz, 2H), 7.83 (ddd, *J* = 9.0, 3.1, 2.2 Hz, 2H), 8.84 (s, 1H), and 10.97 (s, 1H); δ_C_ (126 MHz, DMSO-*d*_6_) 51.4, 101.8, 120.0, 129.9, 130.8, 132.7, 138.1, 162.0, and 162.7; HRMS (ESI): MH^+^, found 253.0372. [C_11_H_10_^35^ClN_2_O_3_]^+^ requires 253.0374.

#### 4.6.2. Methyl 1-(Naphthalen-2-yl)-3-hydroxy-1*H*-pyrazole-4-carboxylate (**2b**)

Prepared from **10b** (220 mg, 0.85 mmol), TBDMAM (263 μL, 1.28 mmol), and anh. toluene (5 mL). Yield: 130 mg (57%) of yellowish solid; mp 168–172 °C (found: C, 67.58; H, 4.52; N, 10.14. C_15_H_12_N_2_O_3_ requires C, 67.16; H, 4.51; N, 10.44%); ν_max_ (ATR) 3099, 2945, 2631, 1696, 1601, 1516, 1292, 1218, 1192, 1119, 892, 857, 813, 770, 745, and 731; δ_H_ (500 MHz, DMSO-*d*_6_) 3.76 (s, 3H), 7.51 (ddd, *J* = 8.1, 6.8, 1.3 Hz, 1H), 7.57 (ddd, *J* = 8.1, 6.8, 1.3 Hz, 1H), 7.95 (dt, *J* = 8.1, 1.4, 1.4 Hz, 2H), 8.03 (d, *J* = 1.4 Hz, 2H), 8.32 (s, 1H), 8.96 (s, 1H), 11.03 (s, 1H); δ_C_ (126 MHz, DMSO-*d*_6_) 50.9, 101.2, 115.0, 117.3, 125.9, 127.1, 127.7, 127.8, 129.4, 131.2, 132.1, 133.0, 136.3, 161.6, and 162.3; HRMS (ESI): MH^+^, found 269.0916. [C_15_H_13_N_2_O_3_]^+^ requires 269.0921.

#### 4.6.3. Methyl 1-(2,4,6-Trichlorophenyl)-3-hydroxy-1*H*-pyrazole-4-carboxylate (**2c**)

Prepared from **10c** (420 mg, 1.35 mmol), TBDMAM (420 μL, 2 mmol), and anh. toluene (5 mL). Yield: 210 mg (48%) of white solid; mp 163–166 °C (found: C, 41.18; H, 2.46; N, 8.50. C_11_H_7_Cl_3_N_2_O_3_ requires C, 41.09; H, 2.19; N, 8.71%); ν_max_ (ATR) 3290, 3039, 2946, 1699, 1650, 1600, 1557, 1483, 1275, 1209, 1172, 1121, 1090, 1024, 874, 784, and 662; δ_H_ (500 MHz, DMSO-*d*_6_) 3.72 (s, 3H), 7.96 (s, 2H), 8.39 (s, 1H), 10.93 (s, 1H); δ_C_ (126 MHz, DMSO-*d*_6_) 51.4, 100.9, 129.3, 134.9, 134.9, 135.9, 138.5, 162.0, and 162.7; HRMS (ESI): MH^+^, found 320.9596. [C_11_H_8_^35^Cl_3_N_2_O_3_]^+^ requires 320.9595.

#### 4.6.4. Methyl 1-(4-Trifluoromethylphenyl)-3-hydroxy-1*H*-pyrazole-4-carboxylate (**2d**)

Prepared from **10d** (130 μL, 0.47 mmol), TBDMAM (145 μL, 0.7 mmol), and anh. toluene (5 mL). Yield: 75 mg (48%) of white solid; mp 198–200 °C (found: C, 50.73; H, 2.99; N, 9.66. C_12_H_9_F_3_N_2_O_3_ requires C, 50.36; H, 3.17; N, 9.78%); ν_max_ (ATR) 3112, 2951, 1694, 1608, 1542, 1339, 1296, 1229, 1151, 1108, 1076, 951, 839, 768, and 654; δ_H_ (500 MHz, DMSO-*d*_6_) 33.75 (s, 3H), 7.84 (d, *J* = 8.5 Hz, 2H), 8.03 (d, *J* = 8.5 Hz, 2H), 9.00 (s, 1H), and 11.17 (s, 1H); δ_C_ (126 MHz, DMSO-*d*_6_) 51.1, 102.2, 118.2, 124.1 (q, *J* = 271.8 Hz), 126.2 (q, *J* = 32.7 Hz), 126.8 (d, *J* = 4.3 Hz), 132.9, 141.6, 161.8, and 162.2; HRMS (ESI): MH^+^, found 287.0644. [C_12_H_10_F_3_N_2_O_3_]^+^ requires 287.0638.

## 5. Conclusions

Ethyl 1-aryl 5-hydroxypyrazole-4-carboxylates **1** are readily obtainable by cyclization of arylhydrazines **4** with diethyl α-[(dimethylamino)methylene]malonate (**3b**). On the other hand, the preparation of isomeric methyl 3-hydroxypyrazole-4-carboxylates **2** is difficult. Therefore, a novel two-step synthesis of **2** from arylhydrazines **4** was developed. The synthetic method involves acylation of hydrazines **4** with methyl 3-chloro-3-oxopropanoate (**9**) to afford the corresponding hydrazides **10**, followed by cyclization of **10** with Bredereck’s reagent to afford 3-hydroxypyrazoles **2**. Testing compounds **1a**–**d** and **2a**–**d** for the inhibition of *Pf*DHODH revealed only weak activities (15–30% inhibition) for compounds **1b** and **2a**–**d**, while compounds **1a**, **1c**, and **1d** showed no significant inhibition of *Pf*DHODH. In general, 3-hydroxy isomers **2** were more effective than 5-hydroxy isomers **1**. Encouragingly, compounds **2b**–**d** were more effective (~30% inhibition) than the known malonate-type inhibitor **3c** (19% inhibition). In summary, isomeric 5- (**1**) and 3-hydroxy-1*H*-pyrazole-4-carboxylates **2** can be prepared in two steps from commercial precursors **3**, **4**, and **9**. Since compounds **1** and **2** showed similar or better inhibition of *Pf*DHODH than the known inhibitor **3c**, this could serve as a starting point for further development of new pyrazolone-based inhibitors of *Pf*DHODH.

## Figures and Tables

**Table 1:** Scores for docking of compounds **1a**–**d**, **2a**–**d**, **3c**, and **12** to *Pf*DHODH.

Entry	Compound	Dscore ^1,2^	Glide ^2^	Penalty ^2^
1	**1a**	−7.80/−6.20	−7.89/−6.29	0.09/0.09
2	**1b**	−6.87/−6.01	−7.07/−6.75	0.20/0.74
3	**1c**	−6.13/−5.52	−7.52/−6.90	1.39/1.39
4	**1d**	−7.68/−6.99	−7.93/−7.24	0.24/0.24
5	**2a**	−8.39/−6.65	−8.82/−7.07	0.43/0.42
6	**2b**	−7.68/−6.36	−8.17/−6.85	0.49/0.49
7	**2c**	−7.97/−7.88	−8.40/−8.28	0.44/0.40
8	**2d**	−8.28/−6.90	−8.90/−7.27	0.61/0.38
9	**3c**	−6.44/−6.40	−6.44/−6.40	0.00/0.00
10	**12**	−9.96/−11.38	−9.97/−11.38	0.01/0.00

^1^ DScore = Glide + Penalty. ^2^ Values obtained by Glide SP/Glide XP.

**Table 2:** Experimental data of compounds **1a**–**d**, **3c**, and **5a**–**d**.

Entry	Compound	R	Yield (%) ^1^
1	**3c**	1*H*-Indazol-5-yl	83 [12]
2	**5a**	4-Chlorophenyl	70 [39]
3	**5b**	Naphthalen-2-yl	86
4	**5c**	2,4,6-Trichlorophenyl	79 [40]
5	**5d**	4-Trifluoromethylphenyl	94
6	**1a**	4-Chlorophenyl	60 [39]
7	**1b**	Naphthalen-2-yl	78
8	**1c**	2,4,6-Trichlorophenyl	86 [40]
9	**1d**	4-Trifluoromethylphenyl	80 [41]

^1^ Isolated yield.

**Table 3:** Experimental data of compounds **8a**–**d** and **10a**–**d**.

Entry	Compound	R	Yield (%) ^1^
1	**10a**	4-Chlorophenyl	47
2	**10b**	Naphthalen-2-yl	48
3	**10c**	2,4,6-Trichlorophenyl	53
4	**10d**	4-Trifluoromethylphenyl	47
5	**2a**	4-Chlorophenyl	74
6	**2b**	Naphthalen-2-yl	57
7	**2c**	2,4,6-Trichlorophenyl	48
8	**2d**	4-Trifluoromethylphenyl	48

^1^ Isolated yield.

**Table 4:** *Pf*DHODH enzyme inhibition data for compounds **1a–d**, **2a–d**, and **3c**.

Entry	Compound	Inhibition (%) ^1^
1	**1a**	*n*.i. ^2^
2	**1b**	29 ± 7
3	**1c**	*n*.i.^2^
4	**1d**	*n*.i.^2^
5	**2a**	15 ± 2
6	**2b**	29 ± 3
7	**2c**	30 ± 2
8	**2d**	24 ± 3
9	**3c**	19 ± 1

^1^ Determined by comparing the rate of DCIP reduction at 25 min with the uninhibited control. ^2^ *n*.i. designates no statistically significant inhibitory effect.

## Data Availability

Data presented in this study are available in the Supplementary Materials.

## Supplementary Materials

The following supporting information can be downloaded at: https://www.mdpi.com/article/10.3390/molecules27154764/s1. Copies of ^1^H and ^13^C NMR spectra of new compounds **3c**, **1a**–**d**, **2a**–**d**, **5a**–**d**, and **10a–d**, copies of IR spectra of compounds **3c**, **1a**–**d**, **2a**–**d**, **5a**–**d**, and **10a–d**; Table S1: Scores for evaluation of binding affinities compounds **3c**, **1a**–**d**, **2a**–**d**, **5a**–**d**, **10a–d**, **A**–**E**, and **12** by molecular docking.

## Sample Availability

Samples of the compounds **1a**–**d**, **3c**, **2a**–**d**, **5a**–**d**, and **10a**–**d** are available from the authors.
