# Supplementary material for: Regioselective Synthesis of 5- and 3-Hydroxy-N-Aryl-1H-Pyrazole-4-Carboxylates and Their Evaluation as Inhibitors of Plasmodium falciparum Dihydroorotate Dehydrogenase
Source: Molecules. 2022 Jul 25;27(15):4764. doi: 10.3390/molecules27154764 (PMC9332393; doi:10.3390/molecules27154764)
Supplement: Supplementary file 1 [file molecules-27-04764-s001.zip › molecules-1813085-supplementary.pdf]

molecules

Supporting Information for the manuscript:

Regioselective Synthesis of 5- and 3-Hydroxy-*N*-Aryl-1*H*-Pyrazole-4-Carboxylates and Their Evaluation as Inhibitors of *Plasmodium Falciparum* Dihydroorotate Dehydrogenase

Luka Vah †, Tadej Medved, Uroš Grošelj, Marina Klemenčič, Črtomir Podlipnik, Bogdan Štefane, Jernej Wagger †, Marko Novinec * and Jurij Svete *

Faculty of Chemistry and Chemical Technology, University of Ljubljana, Večna pot 113, 1000 Ljubljana, Slovenia; luka.vah@novartis.com (L.V.); tm6300@student.uni-lj.si (T.M.); uros.groselj@fkkt.uni-lj.si (U.G.); marina.klemencic@fkkt.uni-lj.si (M.K.); crtomir.podlipnik@fkkt.uni-lj.si (Č.P.); bogdan.stefane@fkkt.uni-lj.si (B.Š.); jernej.wagger@novartis.com (J.W.)

* Correspondence: marko.novinec@fkkt.uni-lj.si (M.N.) and jurij.svete@fkkt.uni-lj.si (J.S.)

† Current address: Lek d.d., Novartis, Chemical Operations, Kolodvorska Cesta 27, 1234 Mengeš, Slovenia.

Table of contents

1.	Copies of NMR spectra of compounds **3c**, **1**, **2**, **5** and **10**.	2–18
2.	Scores for evaluation of binding affinities compounds **3c**, **1a–d**, **2a–d**, **5a–d**, **10a–d**, **A**, **C**, **D**, and **12** by quantum chemical methods.	19–19
3.	Copies of IR spectra of compounds **1a–d**, **2a–d**, **5a–d**, and **10a–d**.	20-24

1. Copies of NMR spectra of compounds 3c, 1a–d, 2a–d, 5a–d and 10a–d.

kt55667.1.fid — LV37

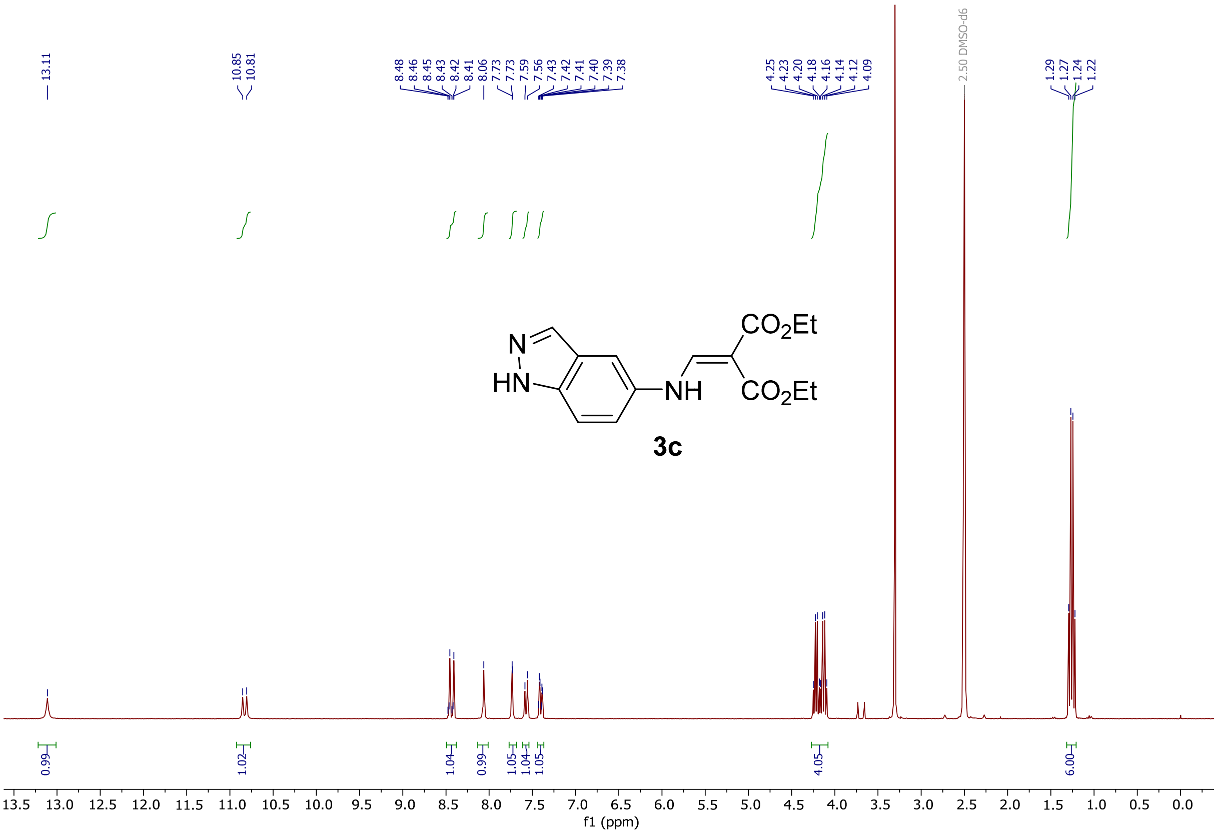

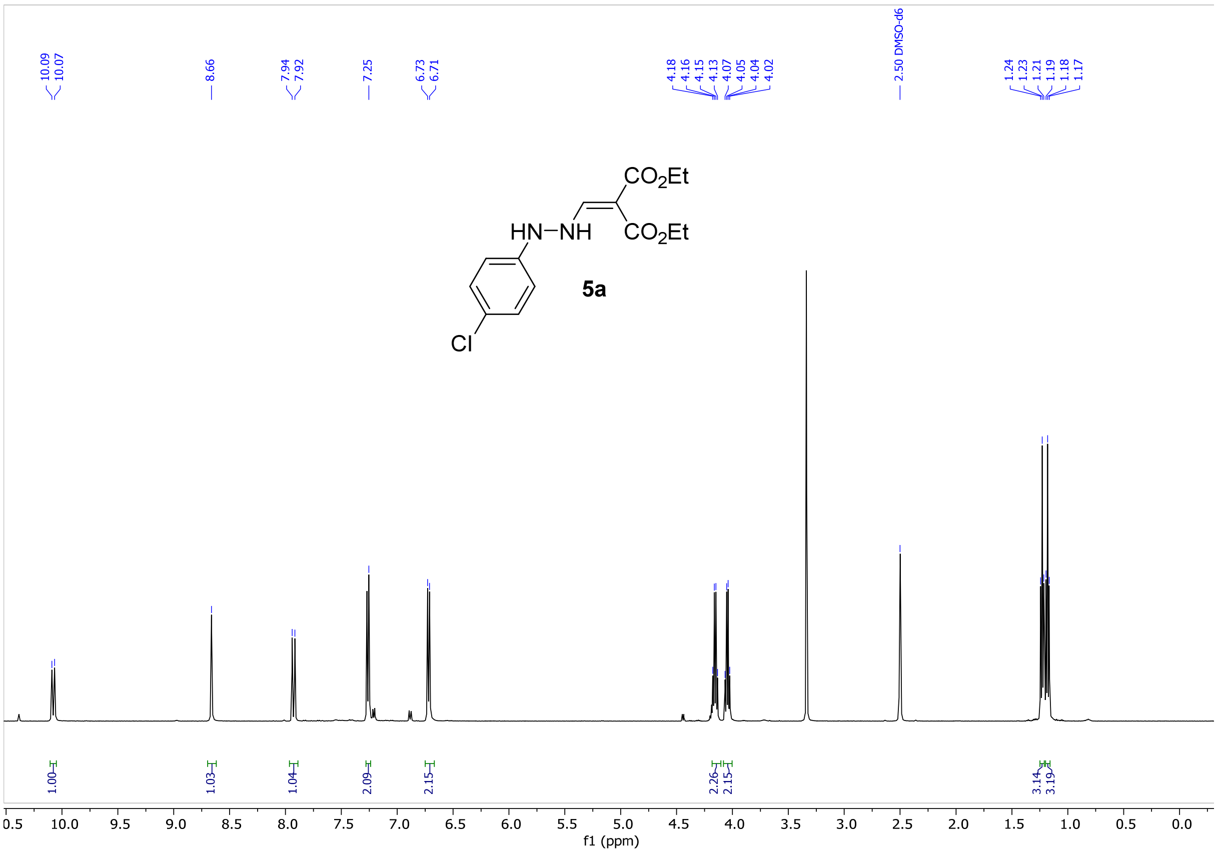
10.09
10.07
8.66
7.94
7.92
7.25
6.73
6.71
4.18
4.16
4.15
4.13
4.07
4.05
4.04
4.02
2.50 DMSO-d6
1.24
1.23
1.21
1.19
1.18
1.17
CO2Et
HN−NH
CO2Et
5a
Cl
1.00
1.03
1.04
2.09
2.15
2.26
2.15
3.14
3.19
f1 (ppm)

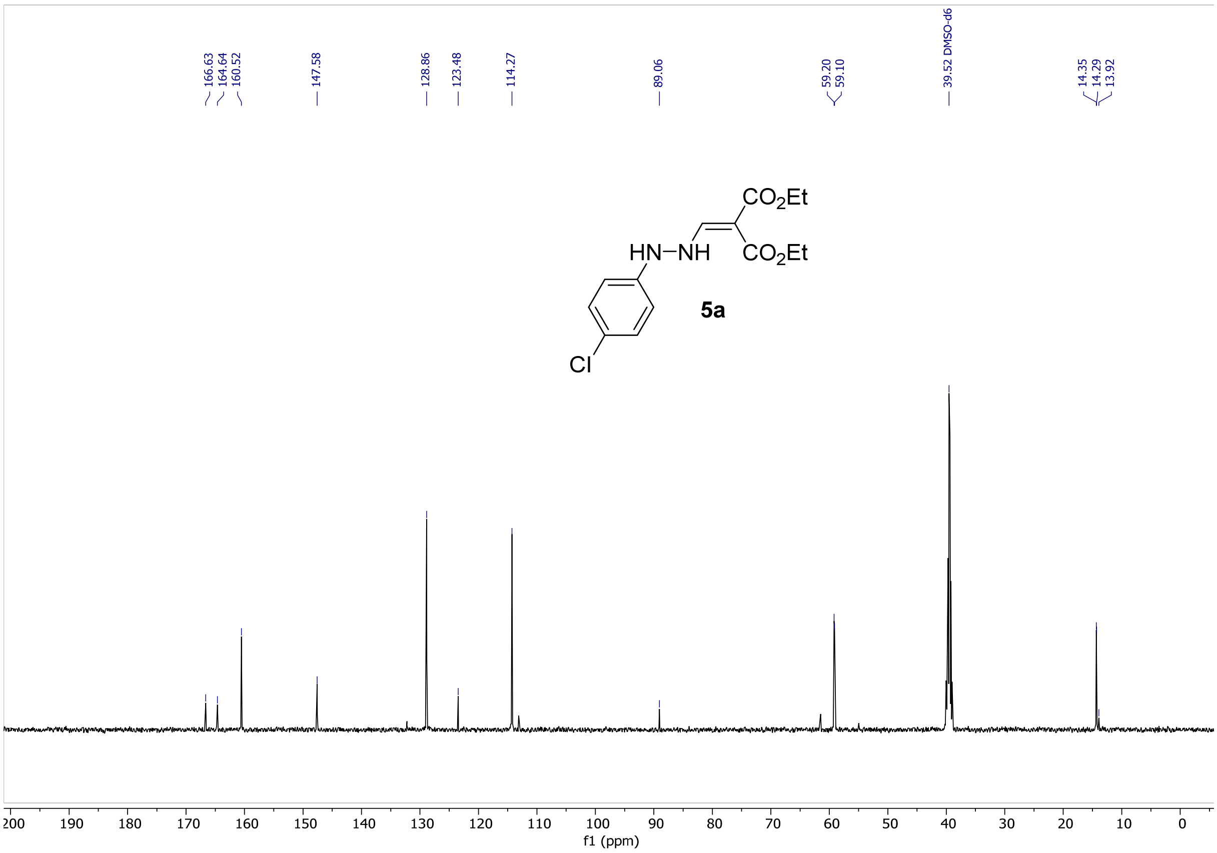
166.63
164.64
160.52
147.58
128.86
123.48
114.27
89.06
59.20
59.10
39.52 DMSO-d6
14.35
14.29
13.92
CO2Et
HN−NH
CO2Et
5a
Cl
f1 (ppm)

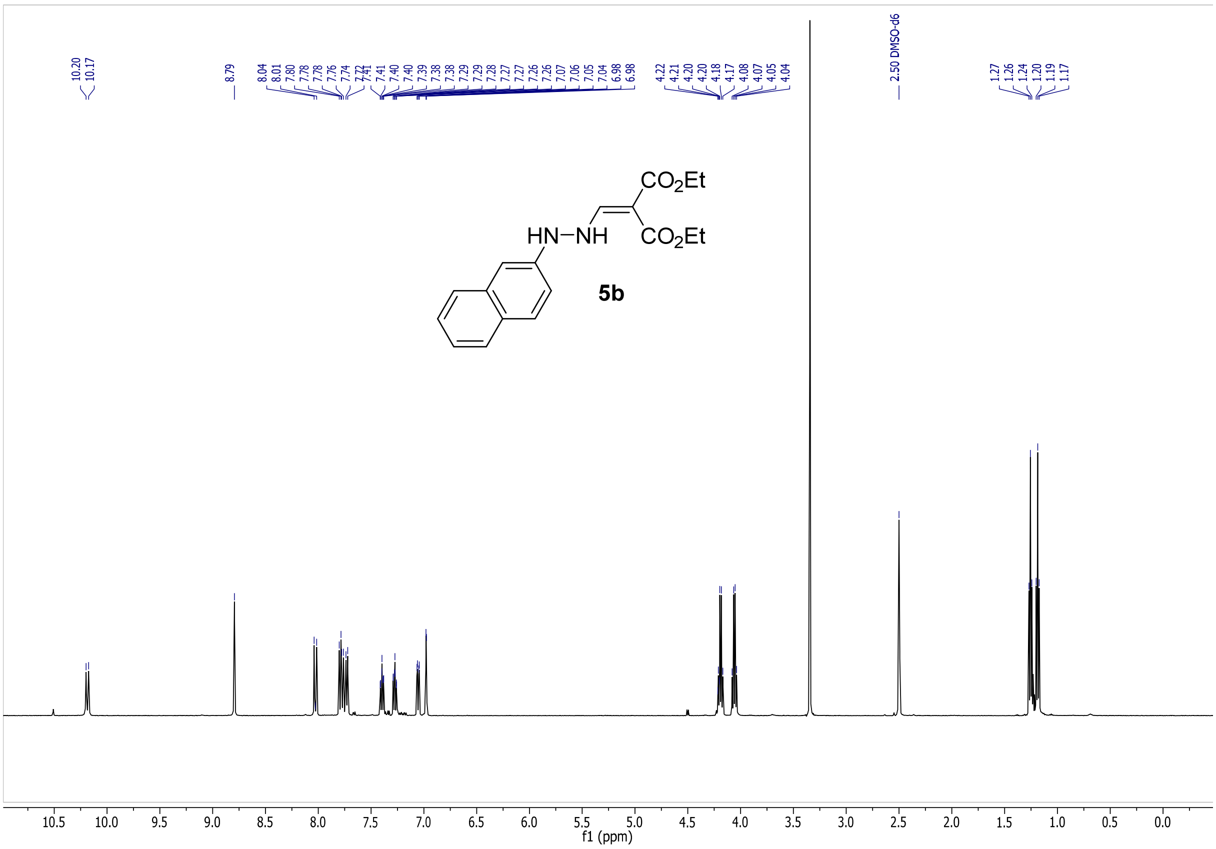
10.20
10.17
8.79
8.04
8.01
7.80
7.78
7.78
7.76
7.74
7.72
7.41
7.41
7.40
7.39
7.38
7.38
7.29
7.29
7.29
7.28
7.27
7.27
7.26
7.26
7.07
7.06
7.05
7.04
6.98
6.98
4.22
4.21
4.20
4.20
4.18
4.17
4.08
4.07
4.05
4.04
2.50 DMSO-d6
1.27
1.26
1.24
1.20
1.19
1.17
CO2Et
HN−NH
CO2Et
5b
f1 (ppm)

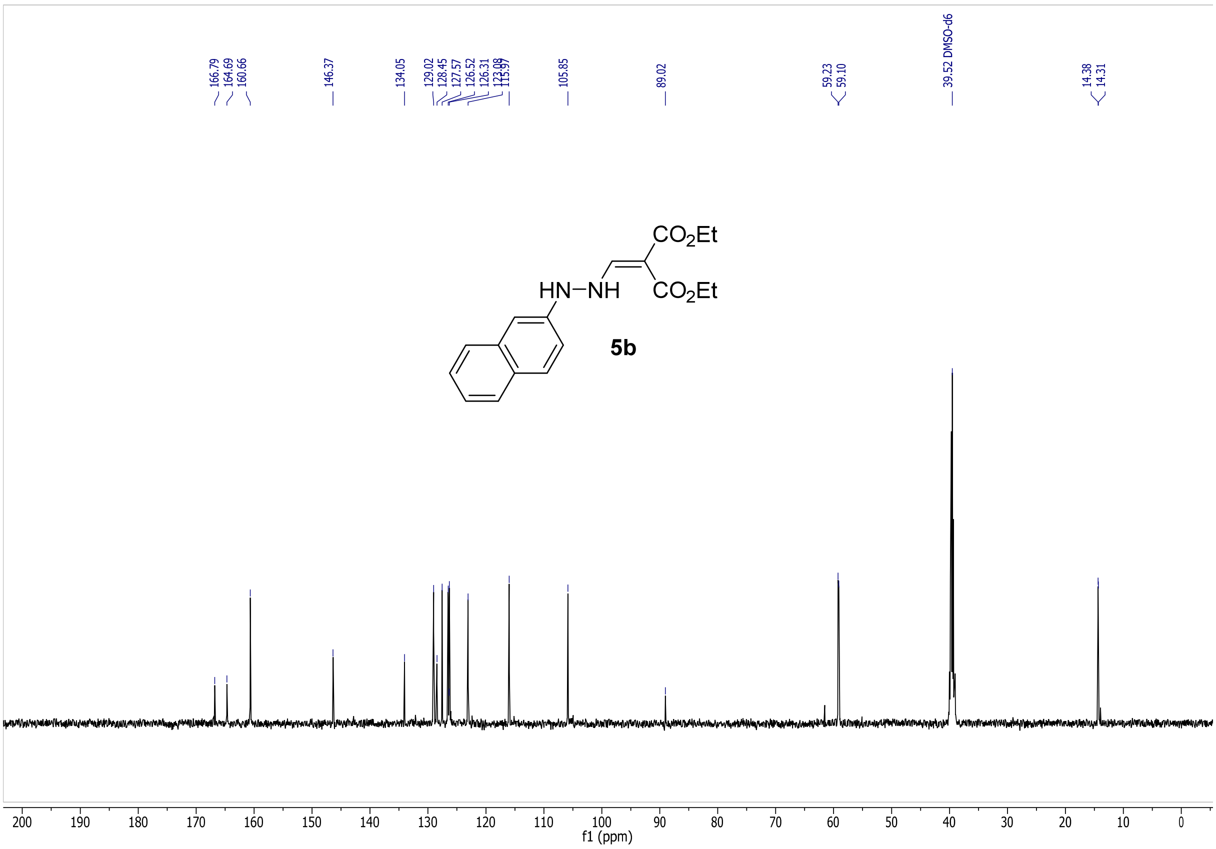
166.79
164.69
160.66
146.37
134.05
129.02
128.45
127.57
126.52
126.31
123.08
115.97
105.85
89.02
59.23
59.10
39.52 DMSO-d6
14.38
14.31
CO2Et
HN−NH
CO2Et
5b
f1 (ppm)

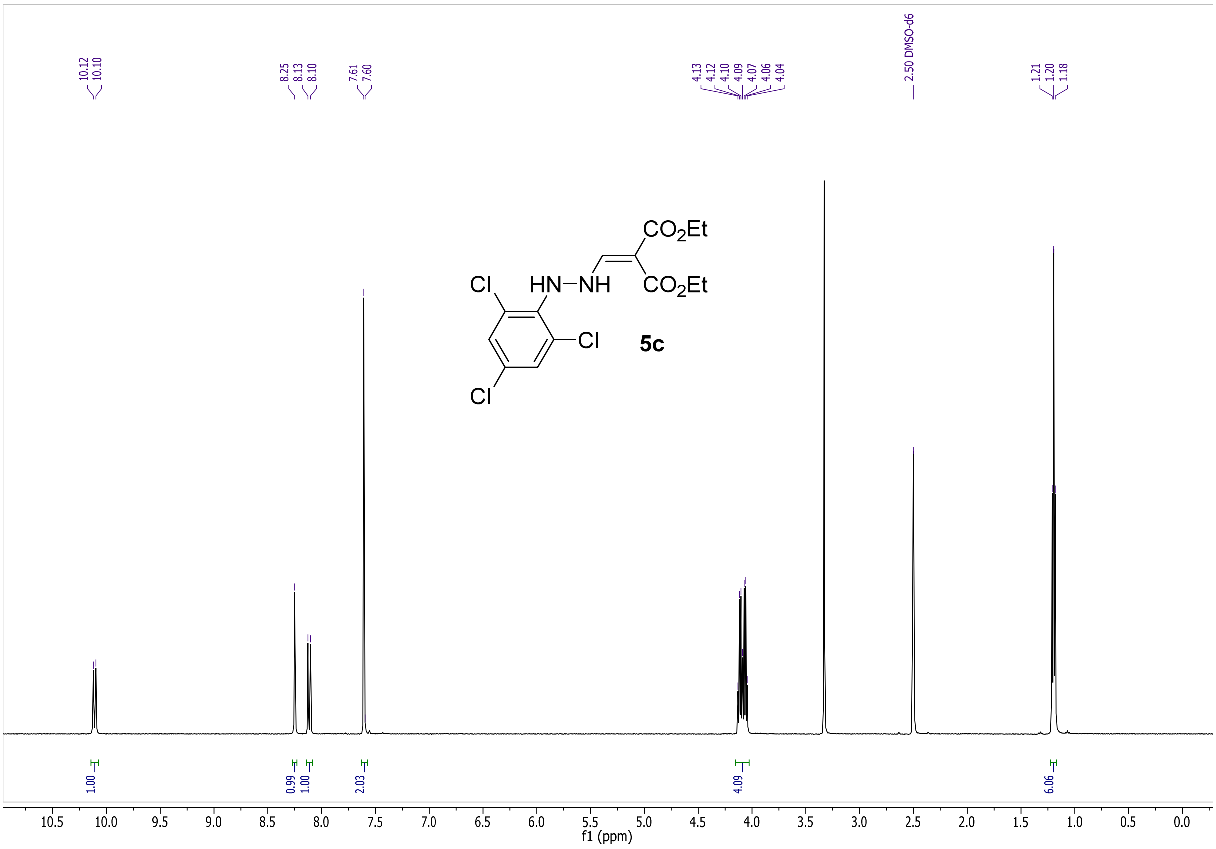
10.12
10.10
8.25
8.13
8.10
7.61
7.60
4.13
4.12
4.10
4.09
4.07
4.06
4.04
2.50 DMSO-d6
1.21
1.20
1.18
CO2Et
Cl
HN–NH
CO2Et
Cl
5c
Cl
1.00
0.99
1.00
2.03
4.09
6.06
f1 (ppm)

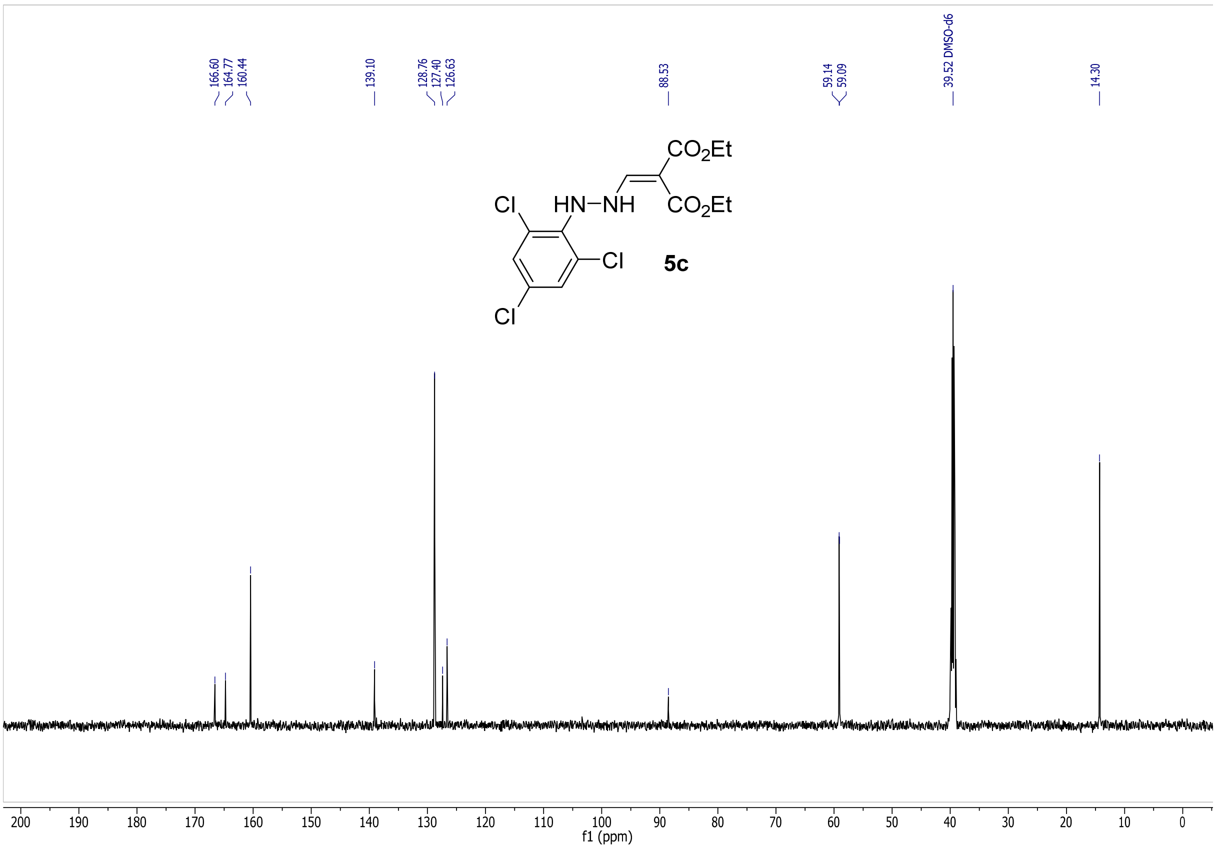
166.60
164.77
160.44
139.10
128.76
127.40
126.63
88.53
59.14
59.09
39.52 DMSO-d6
14.30
CO2Et
Cl
HN–NH
CO2Et
Cl
5c
Cl
f1 (ppm)

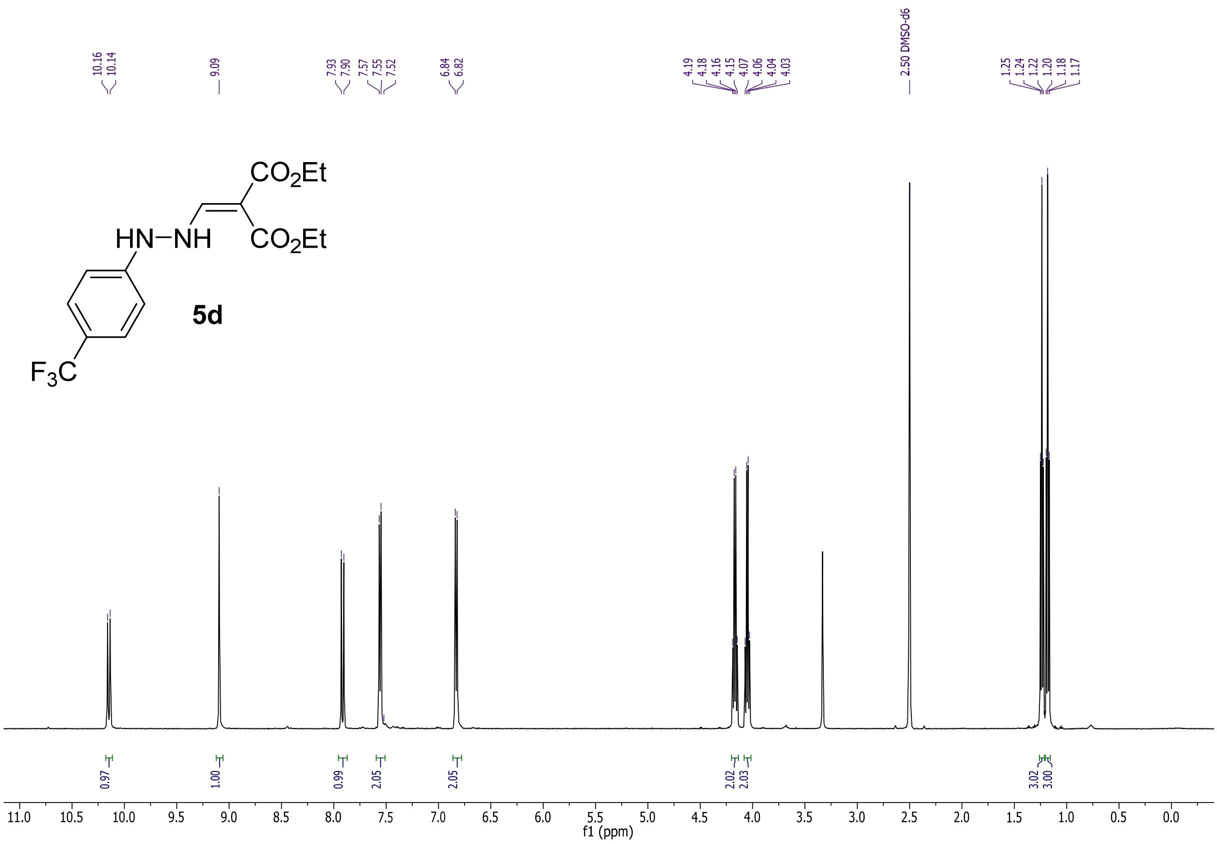
10.16
10.14
9.09
7.93
7.90
7.57
7.55
7.52
6.84
6.82
4.19
4.18
4.16
4.15
4.07
4.06
4.04
4.03
2.50 DMSO-d6
1.25
1.24
1.22
1.20
1.18
1.17
CO2Et
HN–NH
CO2Et
F3C
5d
0.97
1.00
0.99
2.05
2.05
2.02
2.03
3.02
3.00
f1 (ppm)

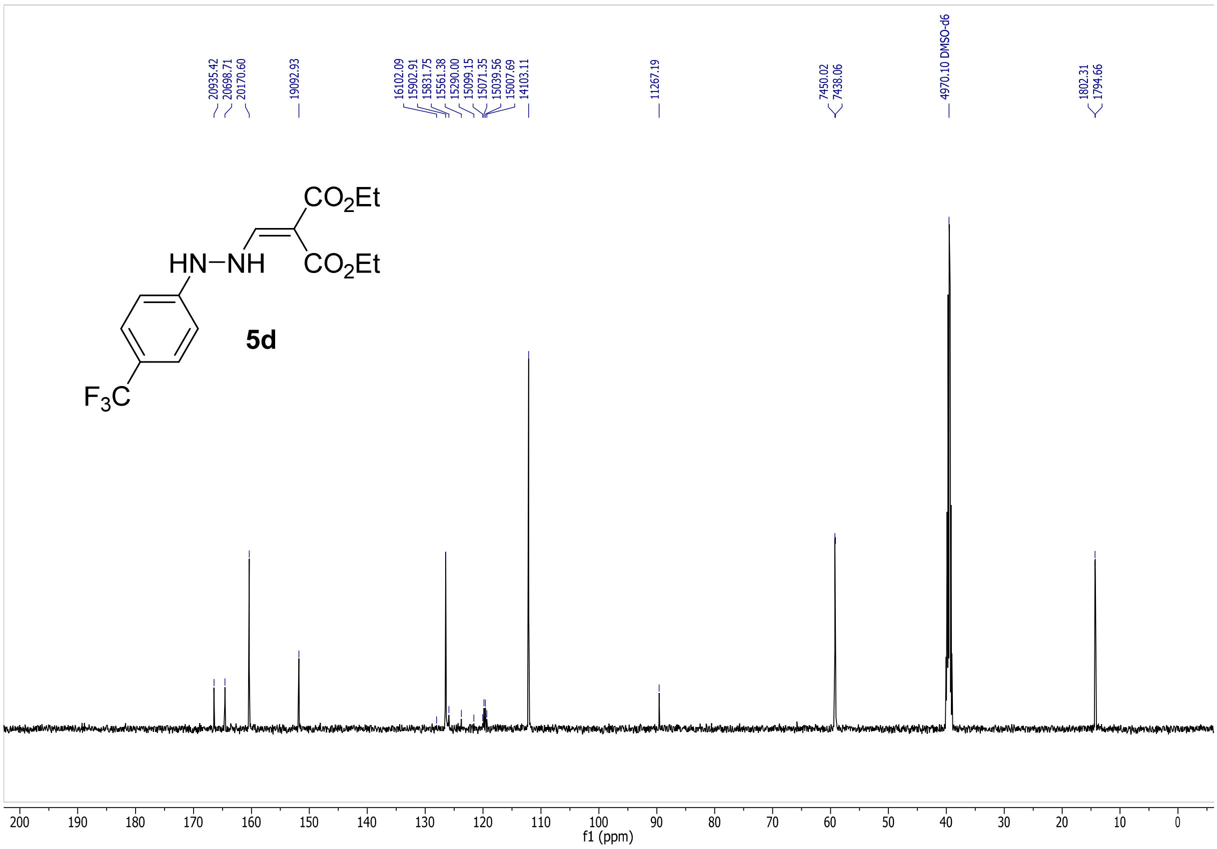
20935.42
20698.71
20170.60
19092.93
16102.09
15902.91
15831.75
15561.38
15290.00
15099.15
15071.35
15039.56
15007.69
14103.11
11267.19
7450.02
7438.06
4970.10 DMSO-d6
1802.31
1794.66
CO2Et
HN–NH
CO2Et
F3C
5d
f1 (ppm)

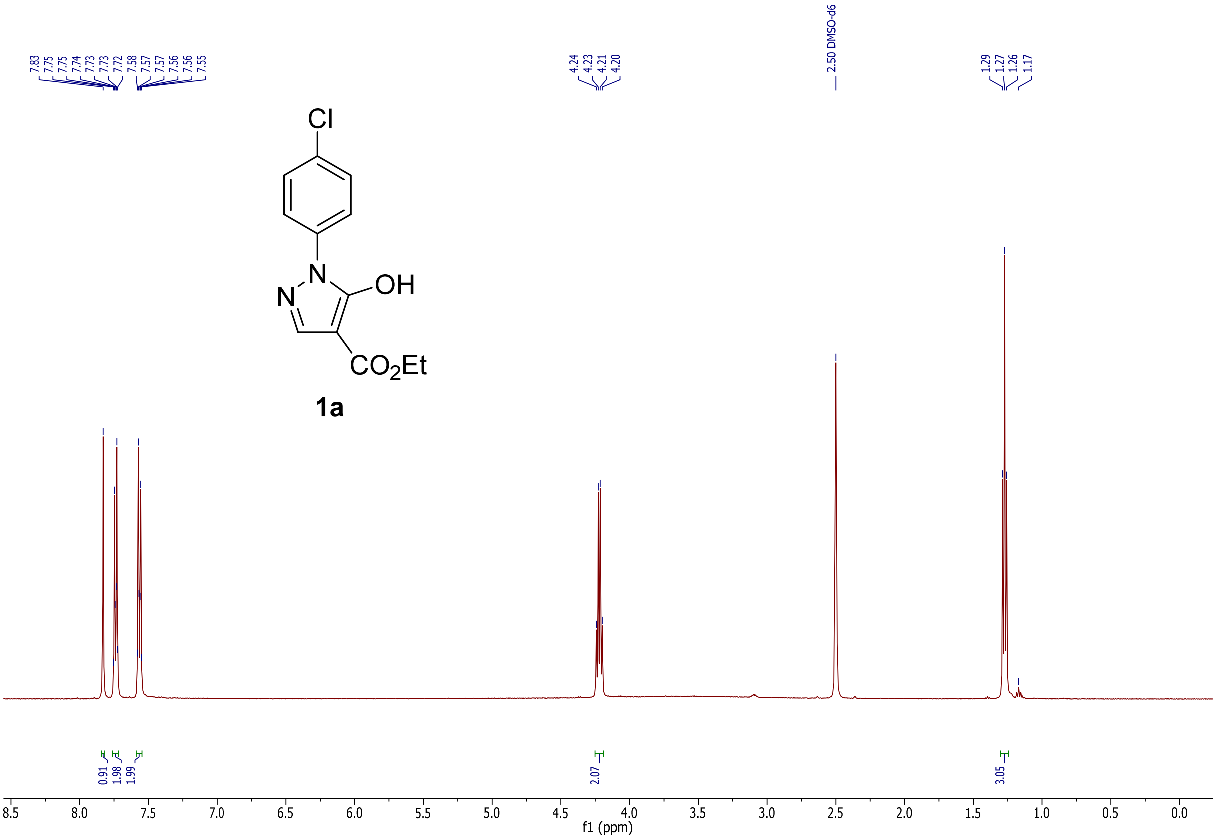
7.83
7.75
7.75
7.74
7.73
7.73
7.72
7.58
7.57
7.57
7.56
7.56
7.55
4.24
4.23
4.21
4.20
2.50 DMSO-d6
1.29
1.27
1.26
1.17
Cl
N
N
OH
CO2Et
1a
0.91
1.98
1.99
2.07
3.05
f1 (ppm)

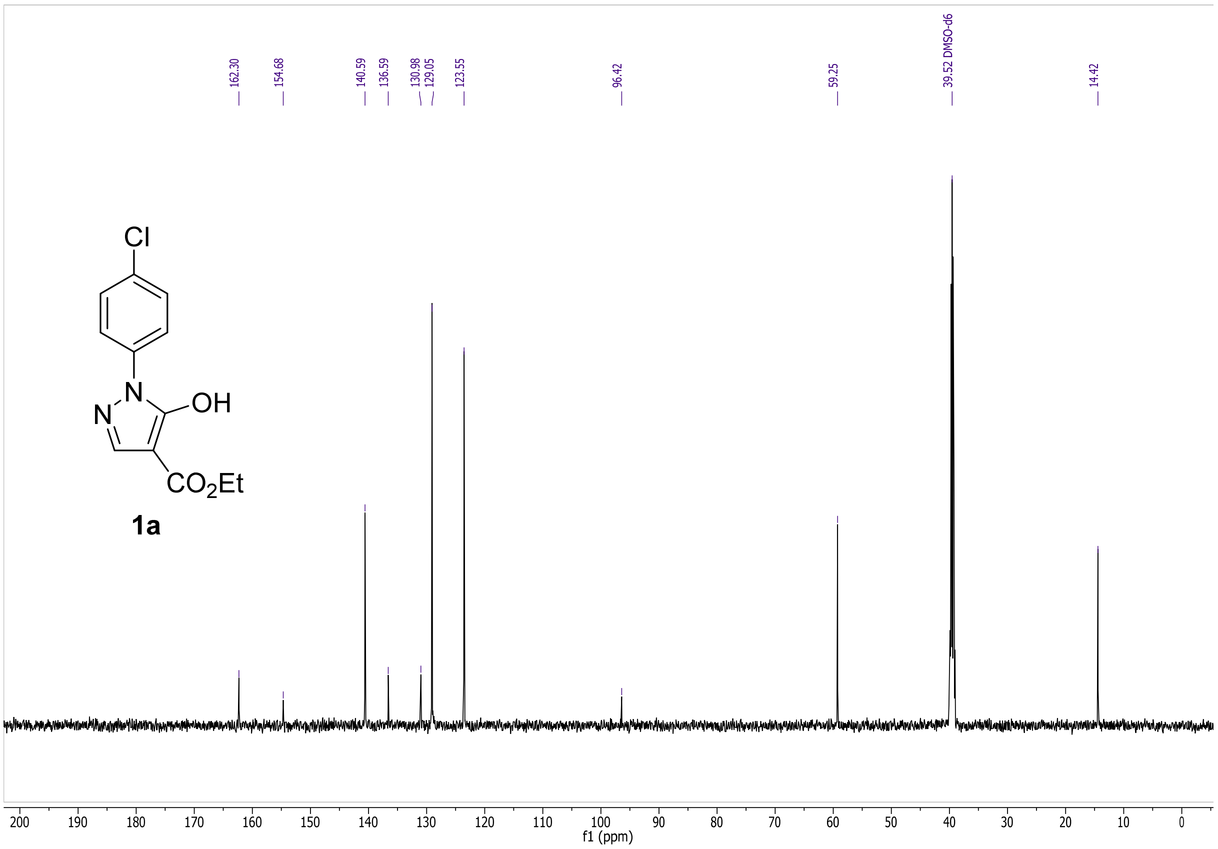
162.30
154.68
140.59
136.59
130.98
129.05
123.55
96.42
59.25
39.52 DMSO-d6
14.42
Cl
N
N
OH
CO2Et
1a
f1 (ppm)

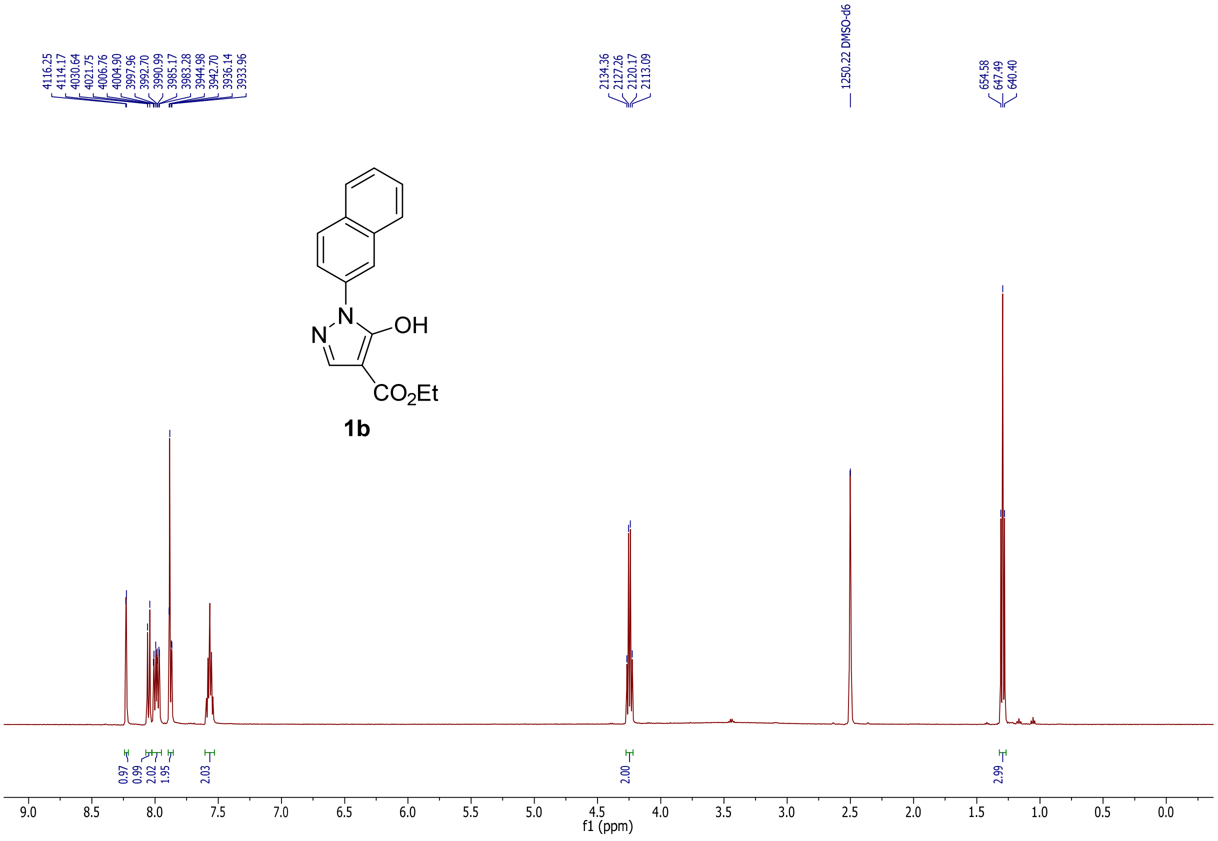
1250.22 DMSO-d6
OH
N
N
CO2Et
1b
0.97
0.99
2.02
1.95
2.03
2.00
2.99
f1 (ppm)

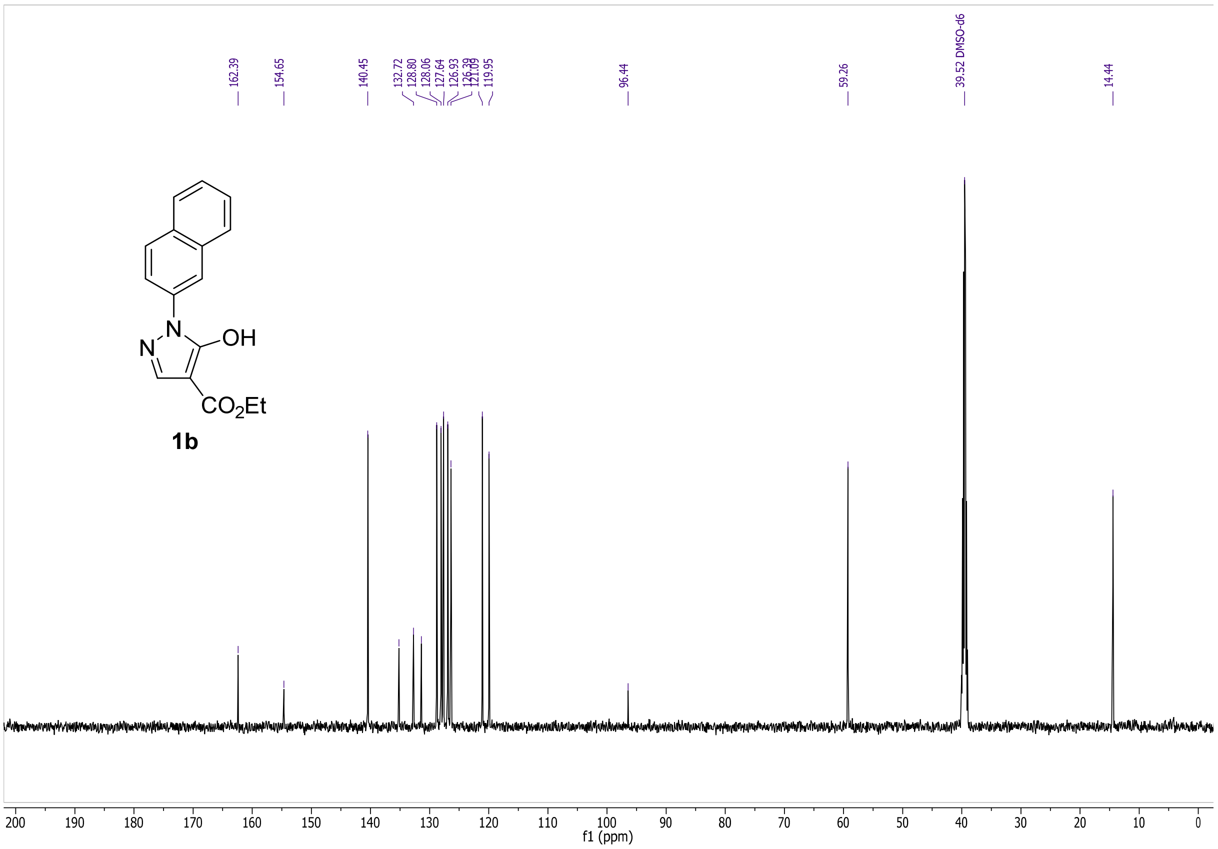
162.39
154.65
140.45
132.72
128.80
128.06
127.64
126.93
119.95
96.44
59.26
39.52 DMSO-d6
14.44
OH
N
N
CO2Et
1b
f1 (ppm)

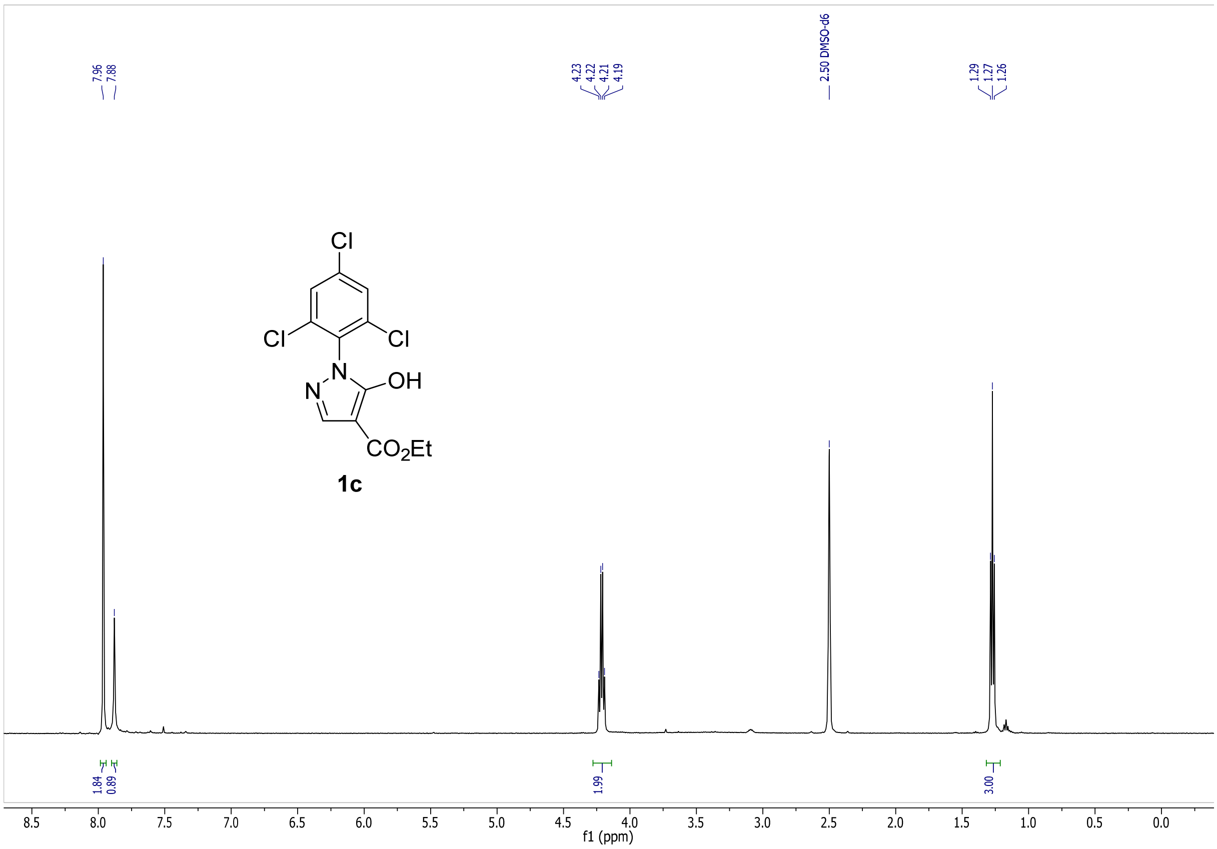
7.96
7.88
4.23
4.22
4.21
4.19
2.50 DMSO-d6
1.29
1.27
1.26
Cl
Cl
Cl
N
N
OH
CO2Et
1c
1.84
0.89
1.99
3.00
f1 (ppm)

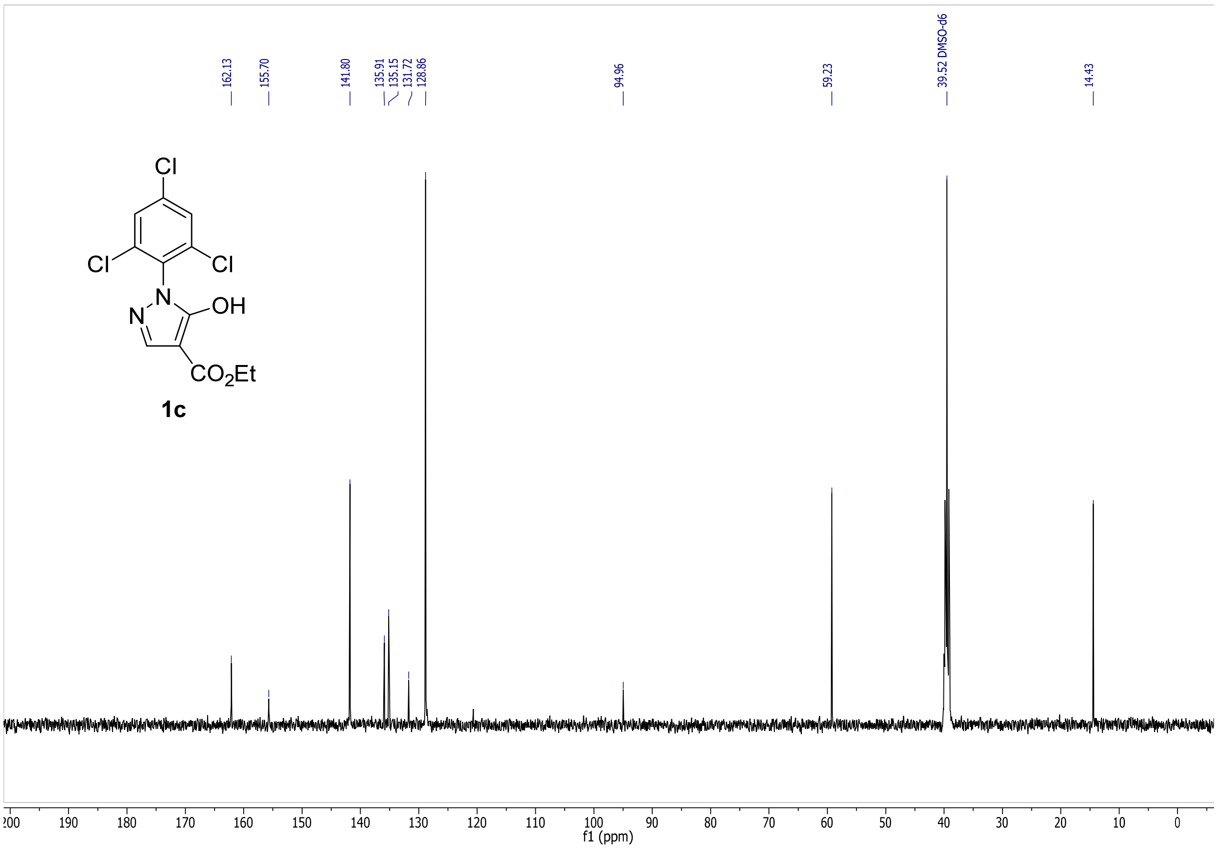
162.13
155.70
141.80
135.91
135.15
131.72
128.86
94.96
59.23
39.52 DMSO-d6
14.43
Cl
Cl
Cl
N
N
OH
CO2Et
1c
f1 (ppm)

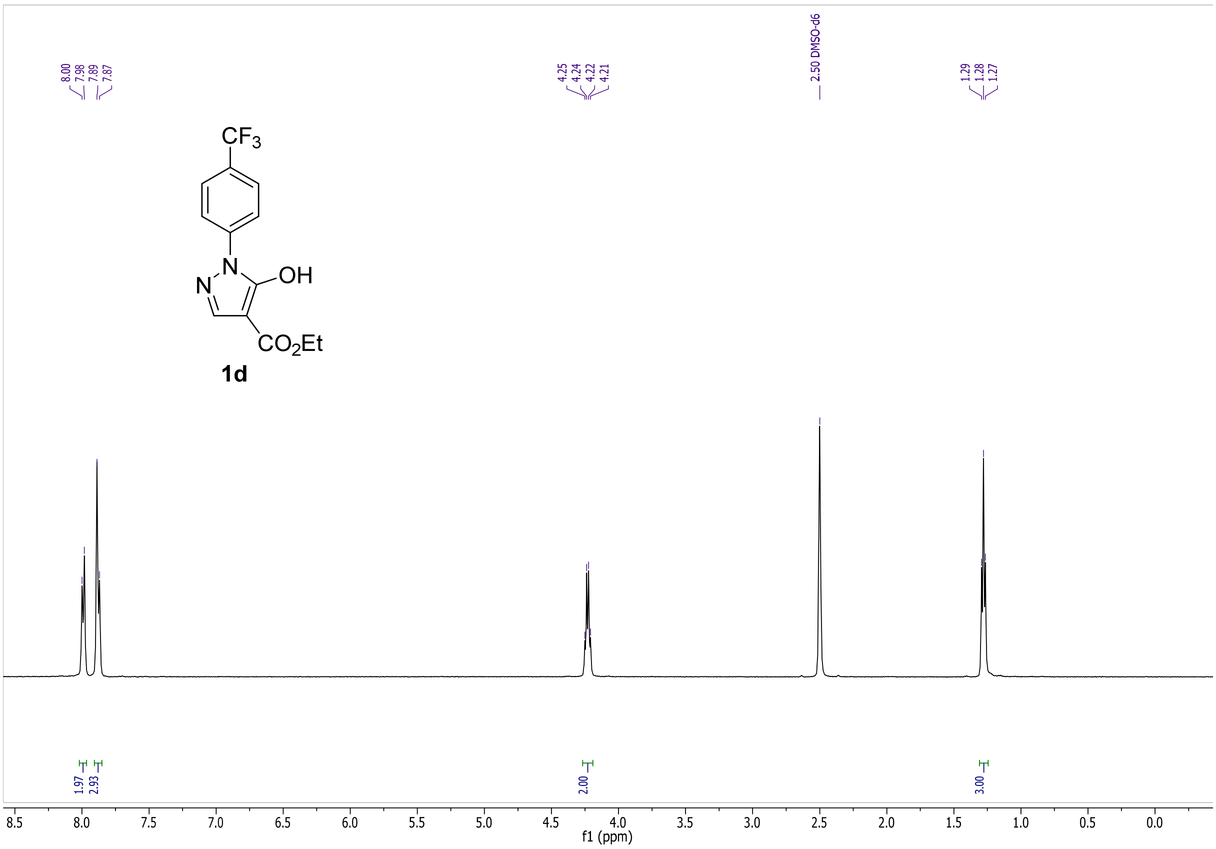
8.00
7.98
7.89
7.87
4.25
4.24
4.22
4.21
2.50 DMSO-d6
1.29
1.28
1.27
CF3
N
N
OH
CO2Et
1d
1.97
2.93
2.00
3.00
f1 (ppm)

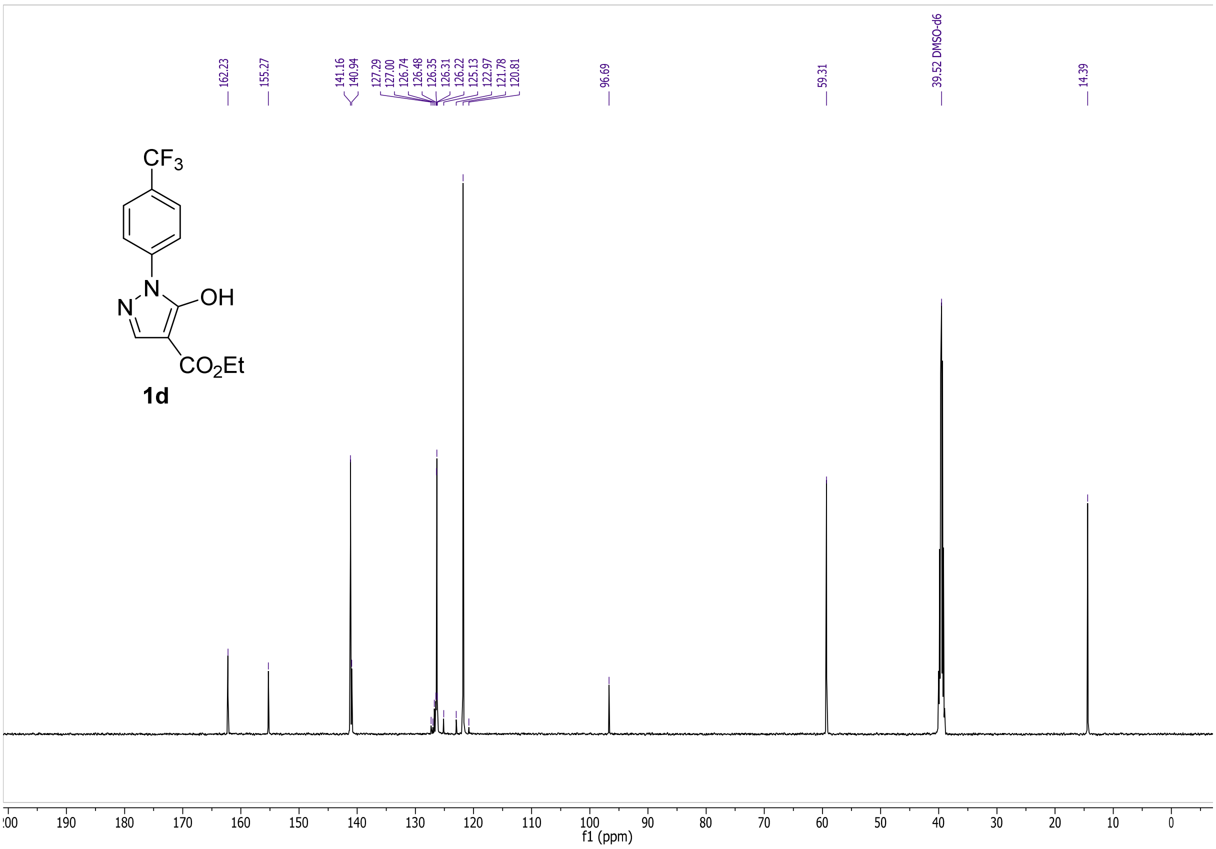
162.23
155.27
141.16
140.94
127.29
127.00
126.74
126.48
126.35
126.31
126.22
125.13
122.97
121.78
120.81
96.69
59.31
39.52 DMSO-d6
14.39
CF3
N
N
OH
CO2Et
1d
f1 (ppm)

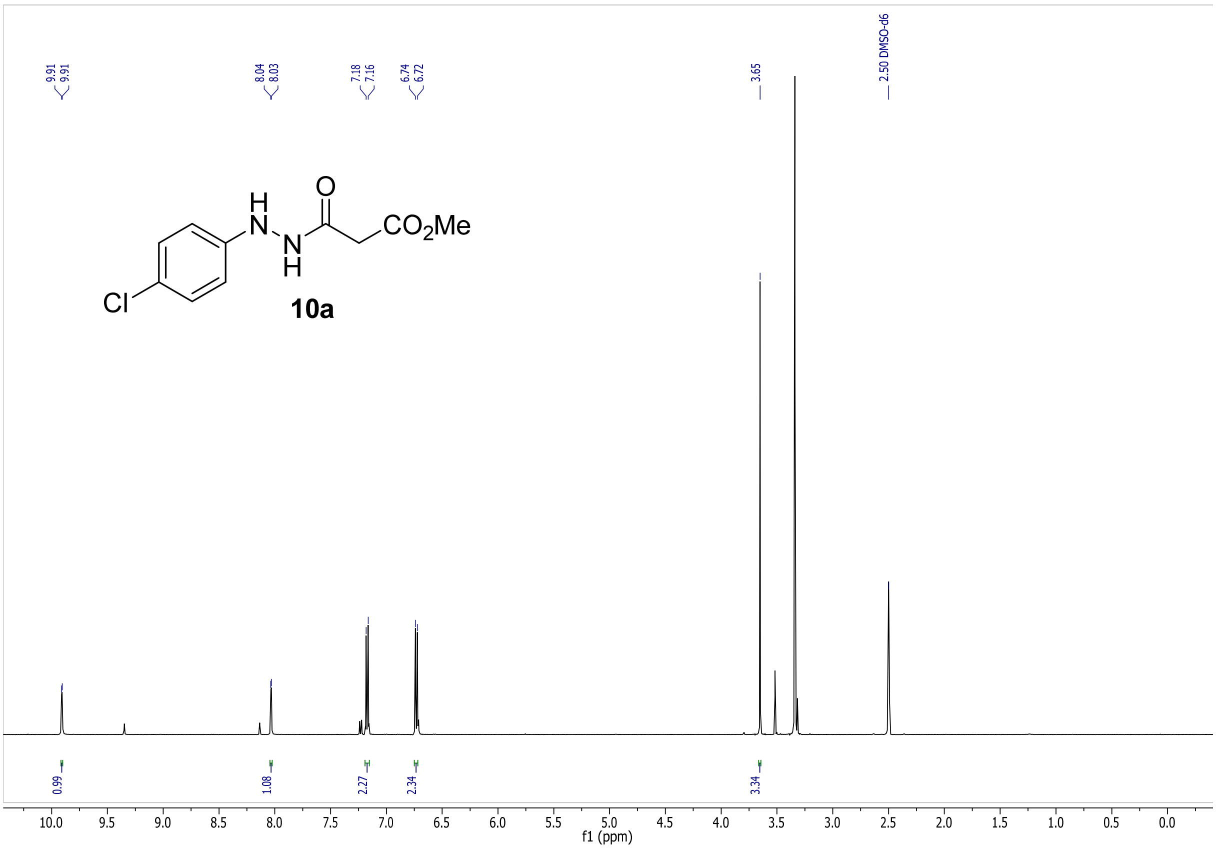
9.91
9.91
8.04
8.03
7.18
7.16
6.74
6.72
3.65
2.50 DMSO-d6
CO2Me
10a
0.99
1.08
2.27
2.34
3.34
f1 (ppm)

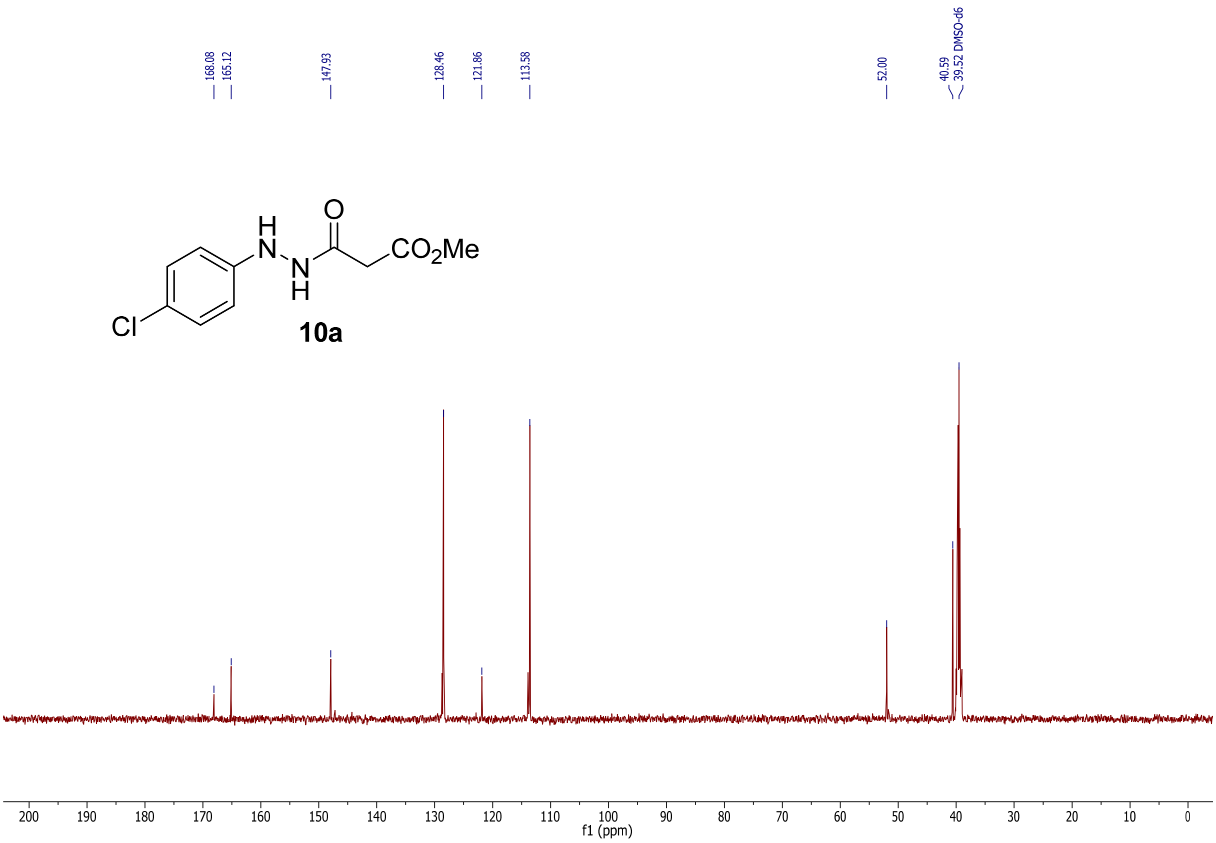
168.08
165.12
147.93
128.46
121.86
113.58
52.00
40.59
39.52 DMSO-d6
CO2Me
10a
f1 (ppm)

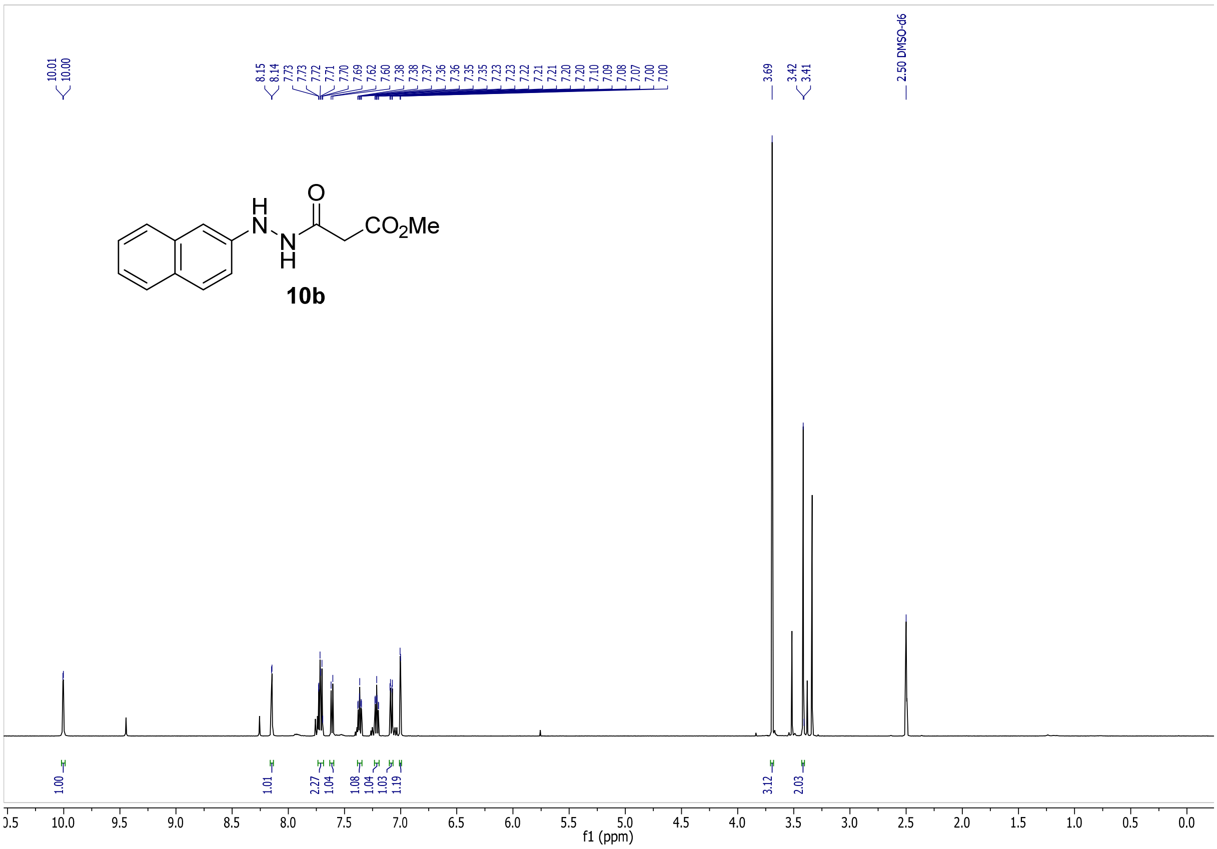
10.01
10.00
8.15
8.14
3.69
3.42
3.41
2.50 DMSO-d6
H
N
N
H
O
CO2Me
10b
1.00
1.01
2.27
1.04
1.08
1.04
1.03
1.19
3.12
2.03
f1 (ppm)

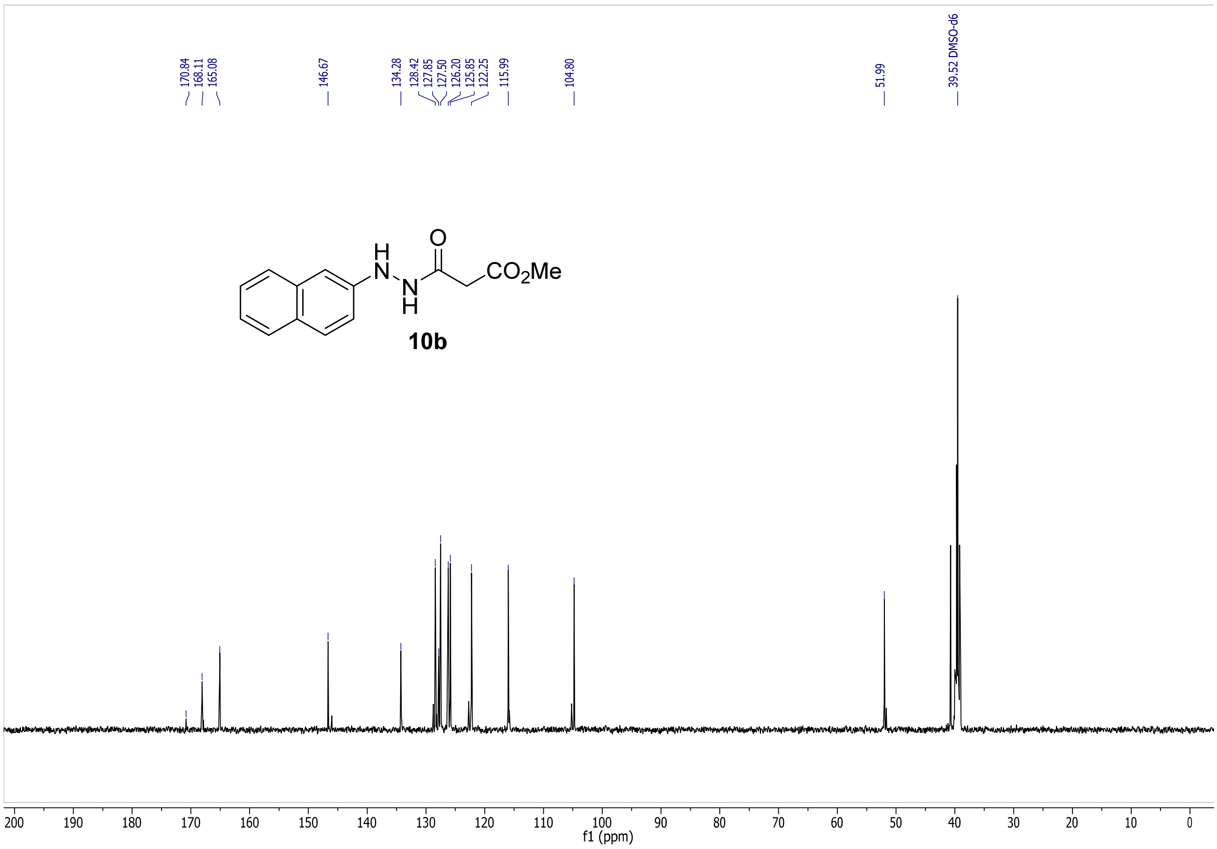
170.84
168.11
165.08
146.67
134.28
128.42
127.85
127.50
126.20
125.85
122.25
115.99
104.80
51.99
39.52 DMSO-d6
H
N
N
H
O
CO2Me
10b
f1 (ppm)

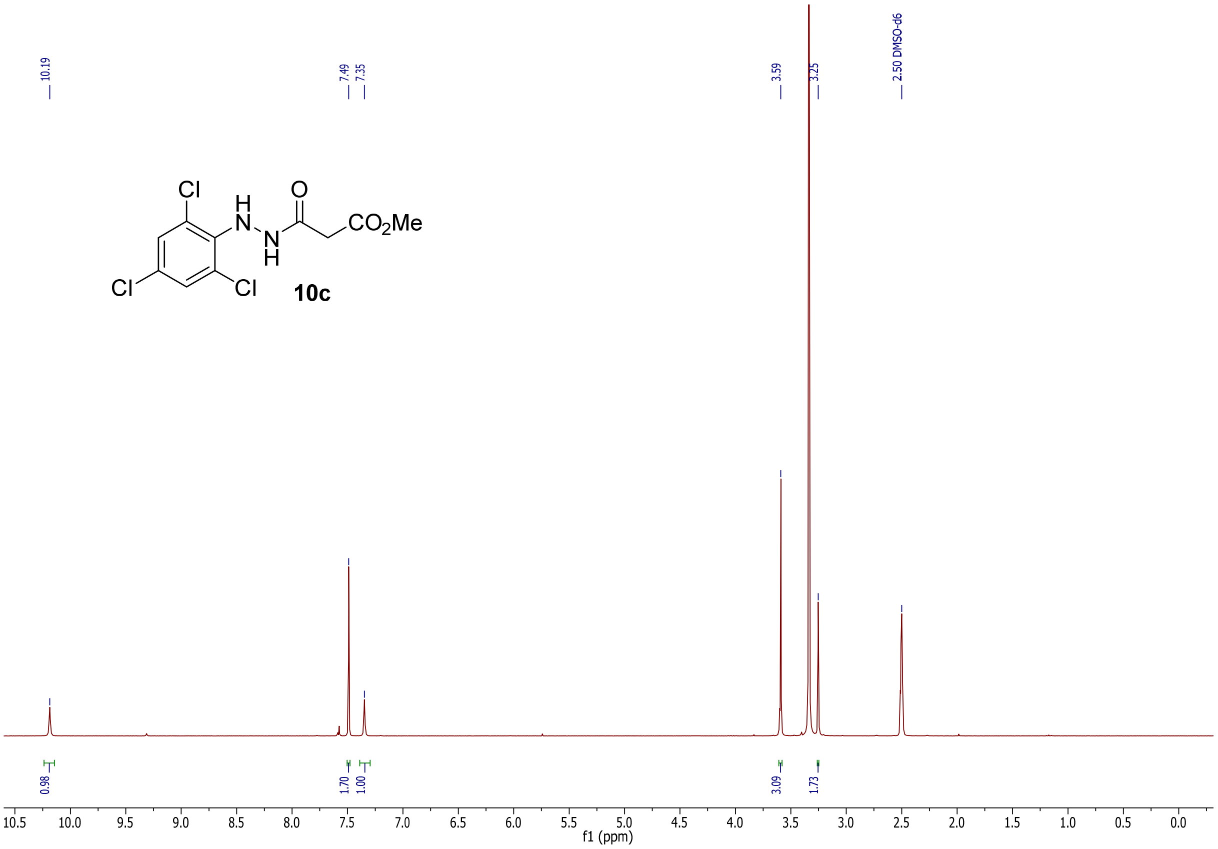
10.19
7.49
7.35
3.59
3.25
2.50 DMSO-d6
CO2Me
10c
0.98
1.70
1.00
3.09
1.73
f1 (ppm)

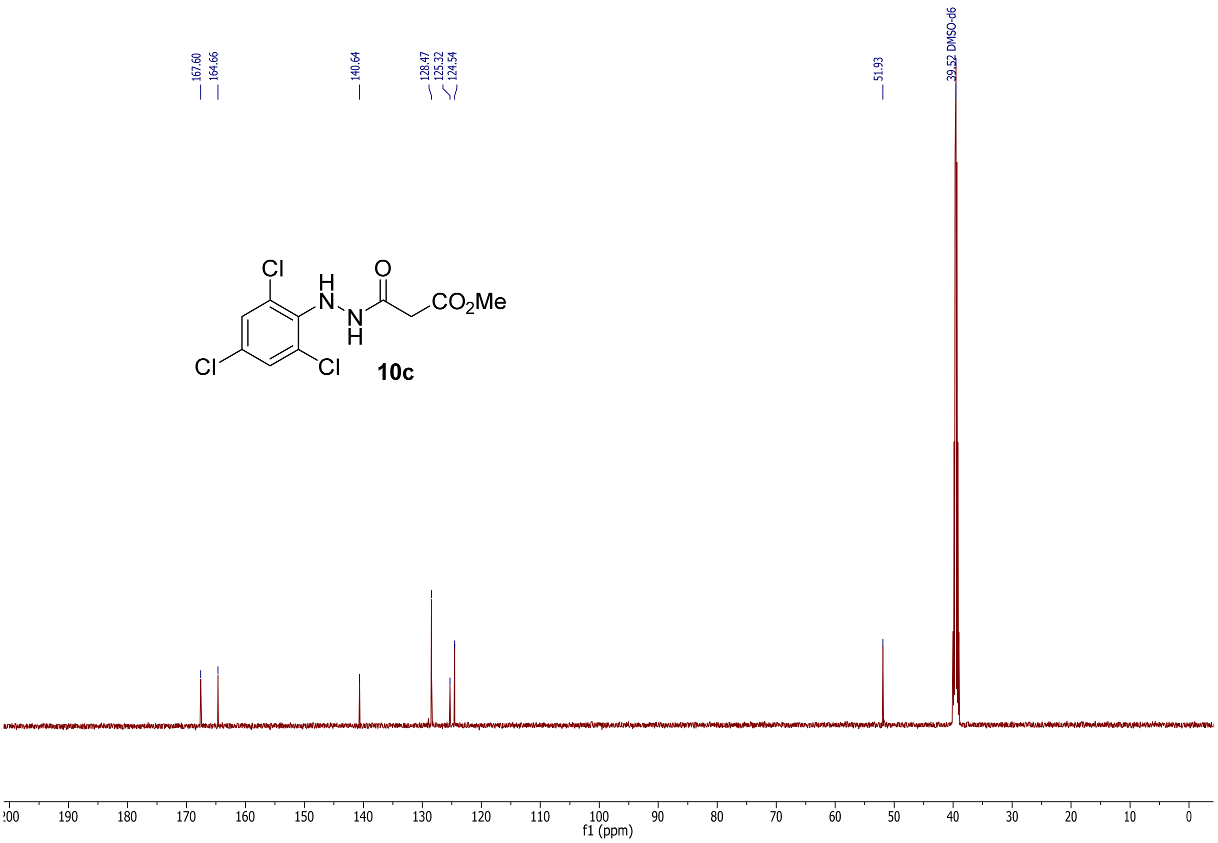
167.60
164.66
140.64
128.47
125.32
124.54
51.93
39.52 DMSO-d6
CO2Me
10c
f1 (ppm)

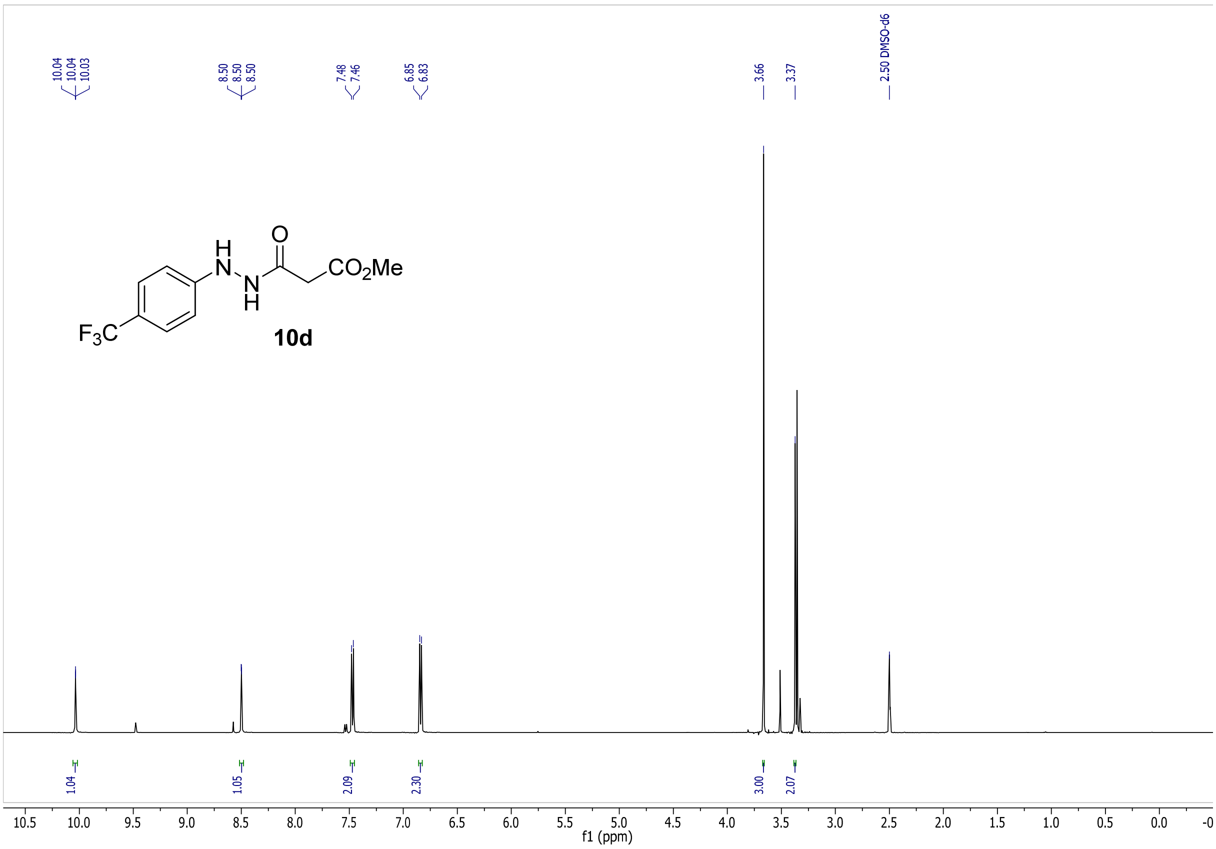

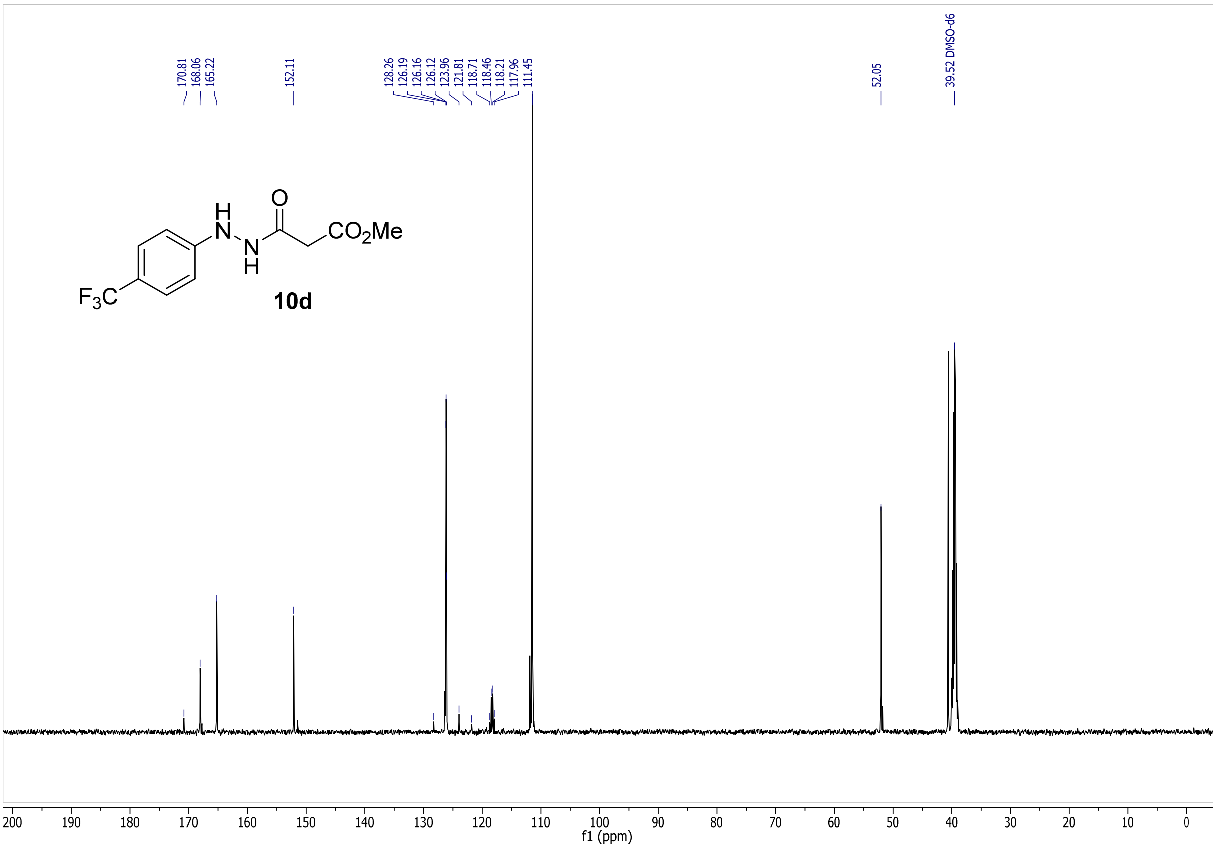

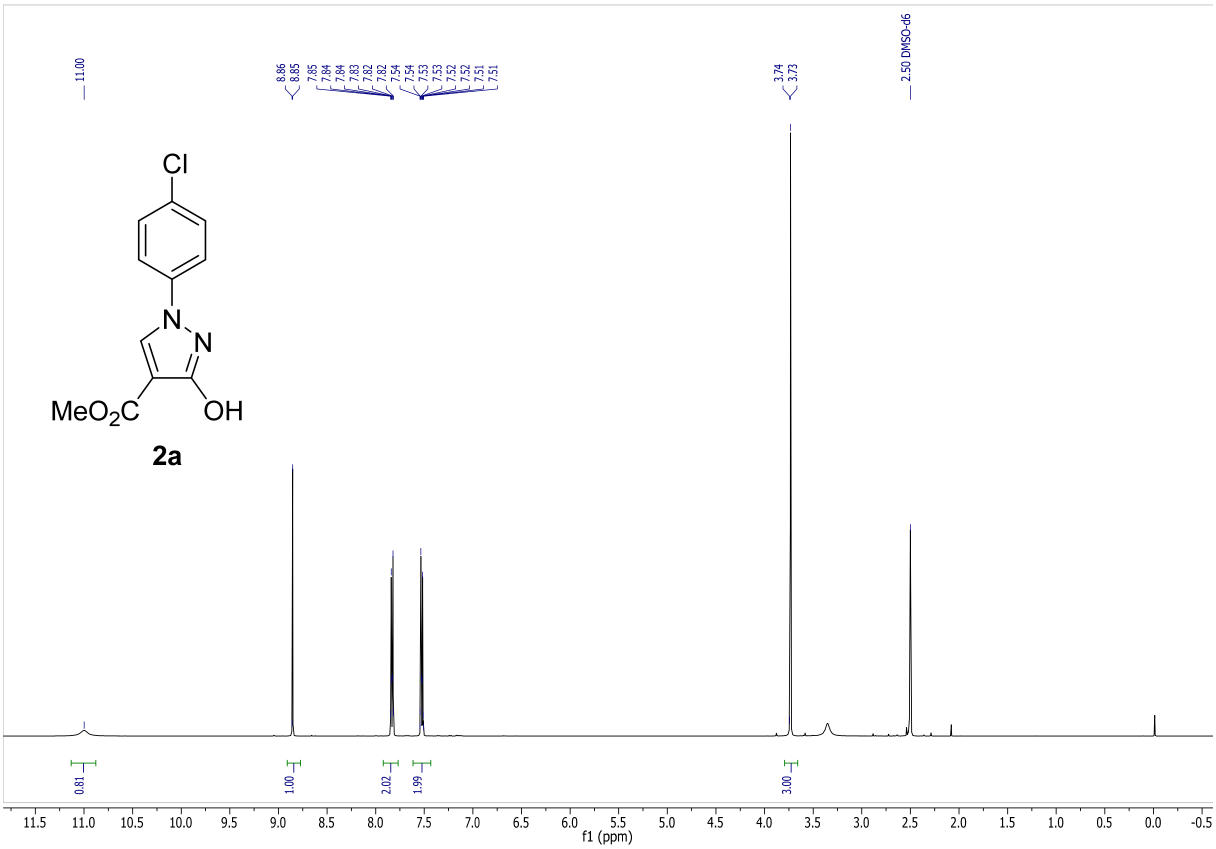
11.00
8.86
8.85
7.85
7.84
7.84
7.83
7.82
7.82
7.82
7.54
7.54
7.53
7.53
7.52
7.52
7.51
7.51
3.74
3.73
2.50 DMSO-d6
Cl
N
N
MeO2C
OH
2a
0.81
1.00
2.02
1.99
3.00
f1 (ppm)

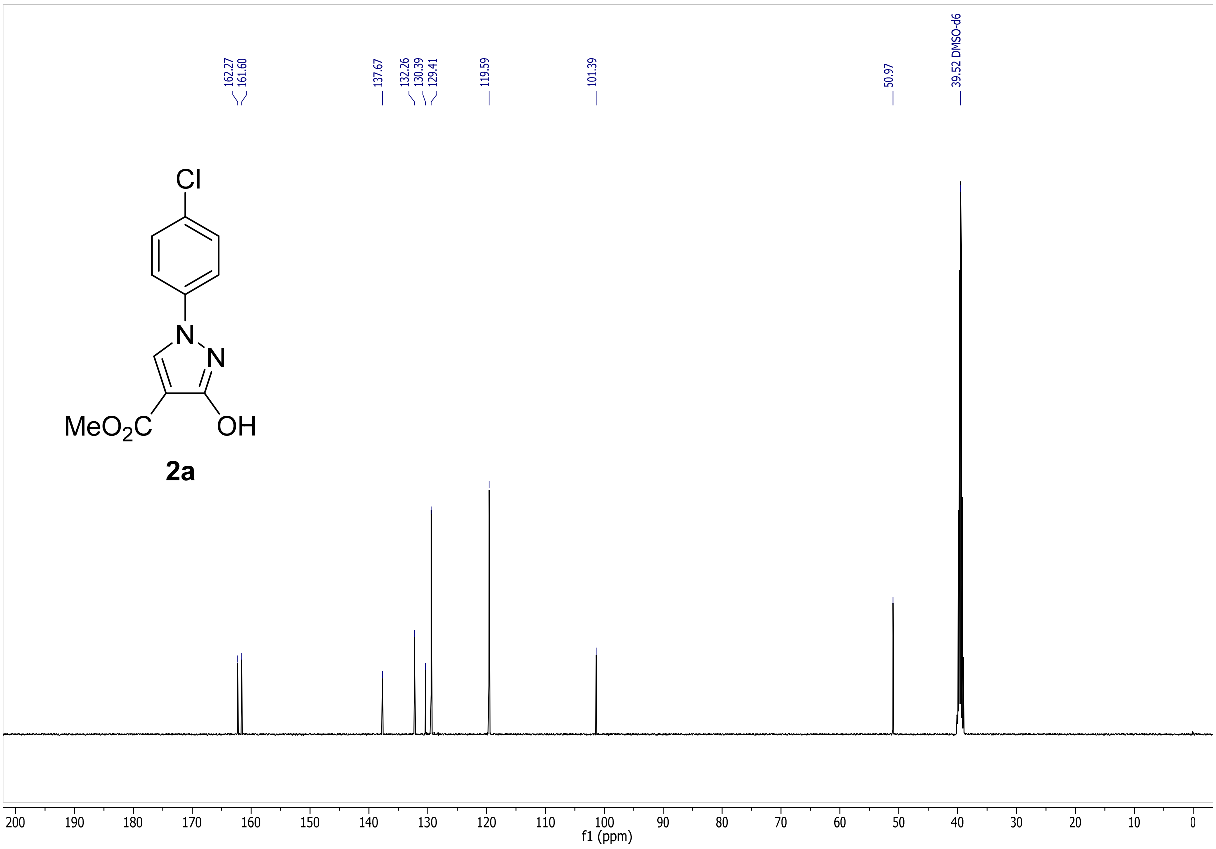
162.27
161.60
137.67
132.26
130.39
129.41
119.59
101.39
50.97
39.52 DMSO-d6
Cl
N
N
MeO2C
OH
2a
f1 (ppm)

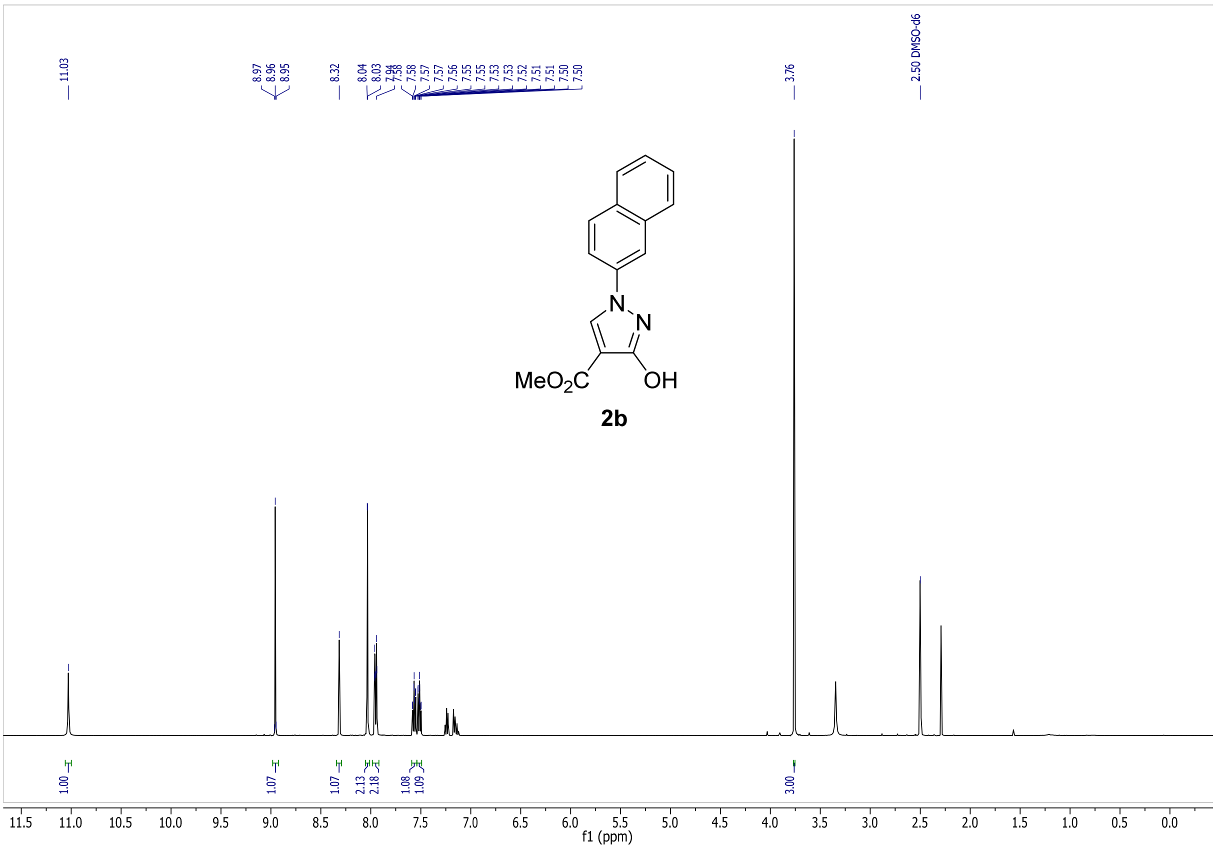

2b
MeO2C
OH
f1 (ppm)

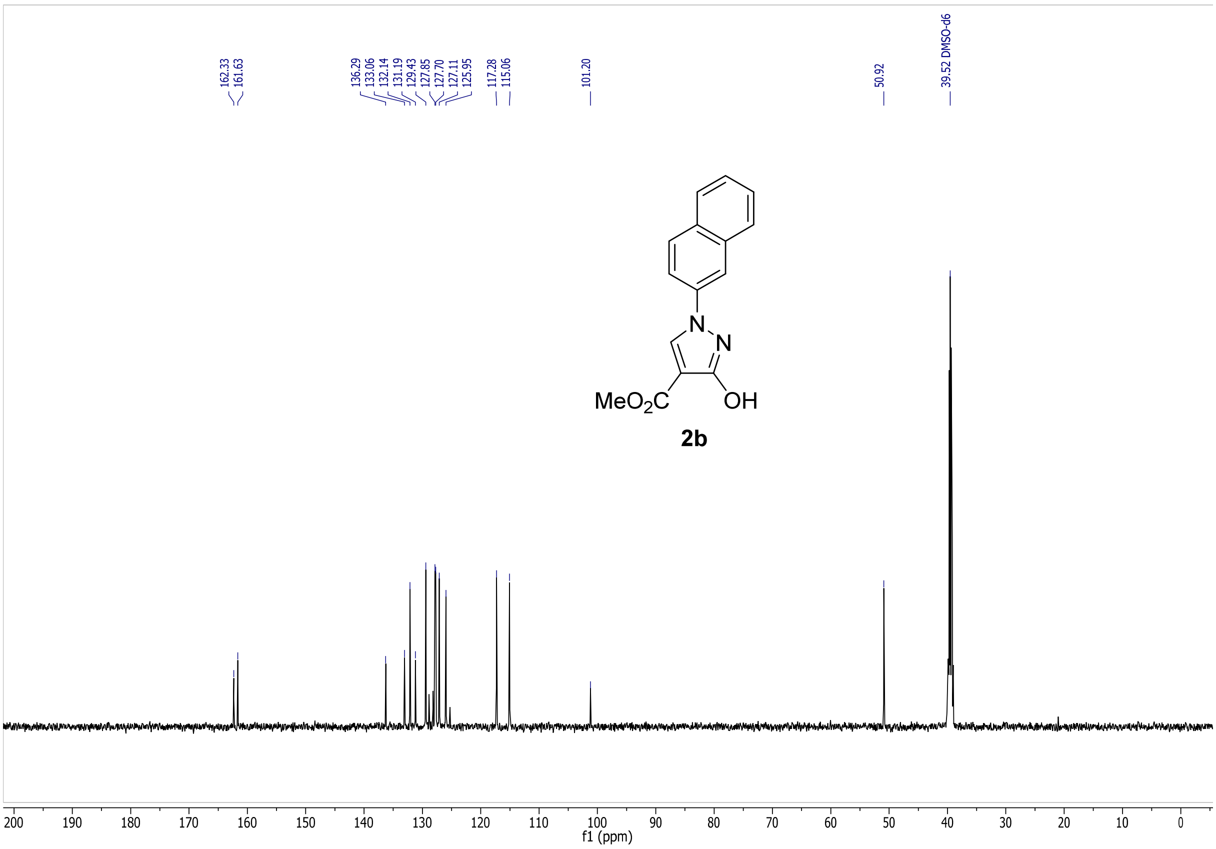

2b
MeO2C
OH
f1 (ppm)

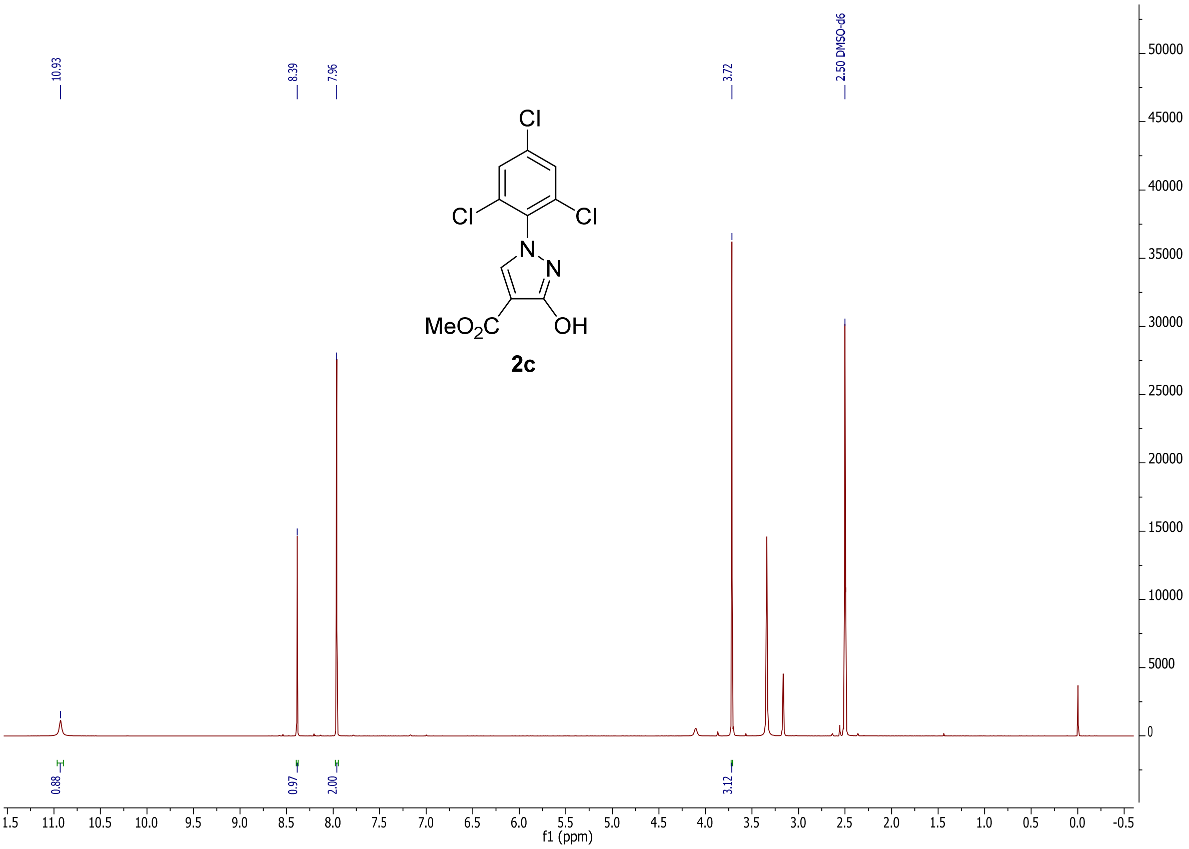
10.93
8.39
7.96
3.72
2.50 DMSO-d6
Cl
Cl
Cl
N
N
MeO2C
OH
2c
0.88
0.97
2.00
3.12
f1 (ppm)

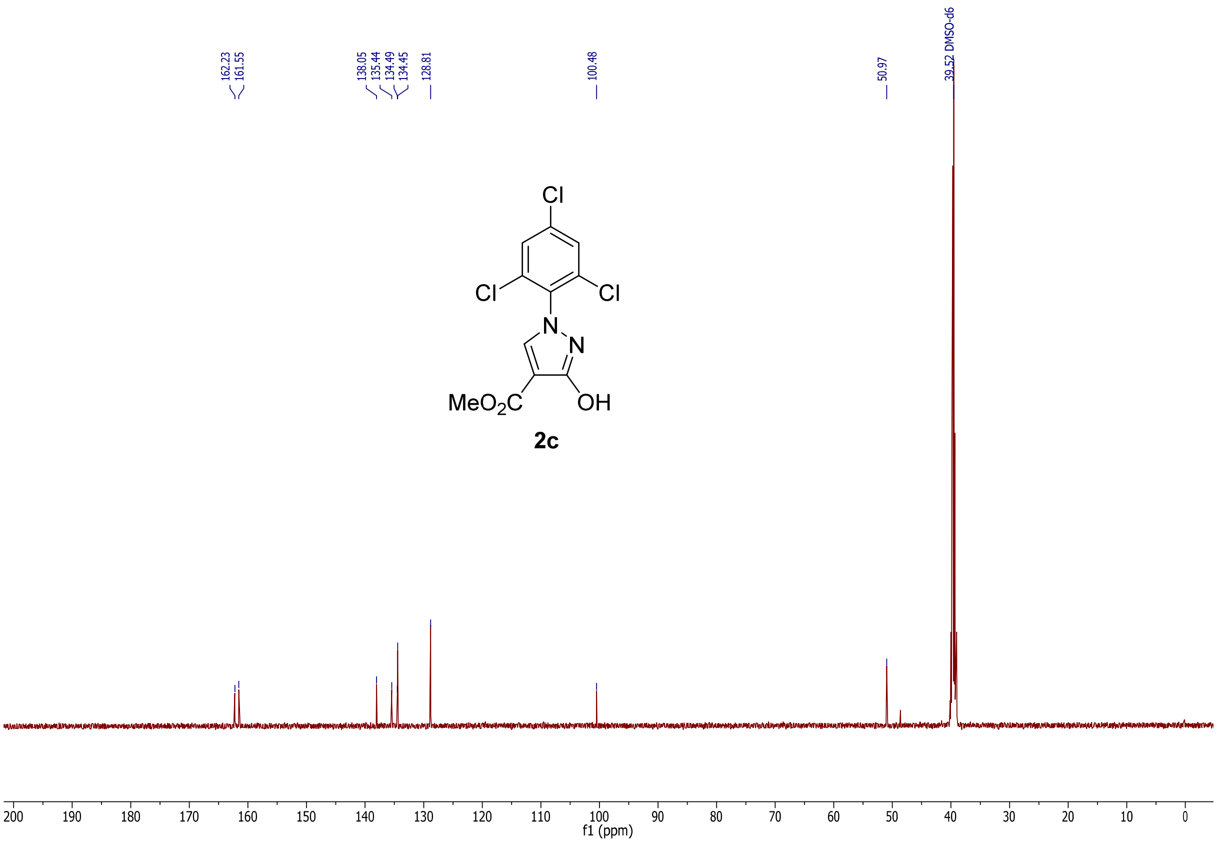
162.23
161.55
138.05
135.44
134.49
134.45
128.81
100.48
50.97
39.52 DMSO-d6
Cl
Cl
Cl
N
N
MeO2C
OH
2c
f1 (ppm)

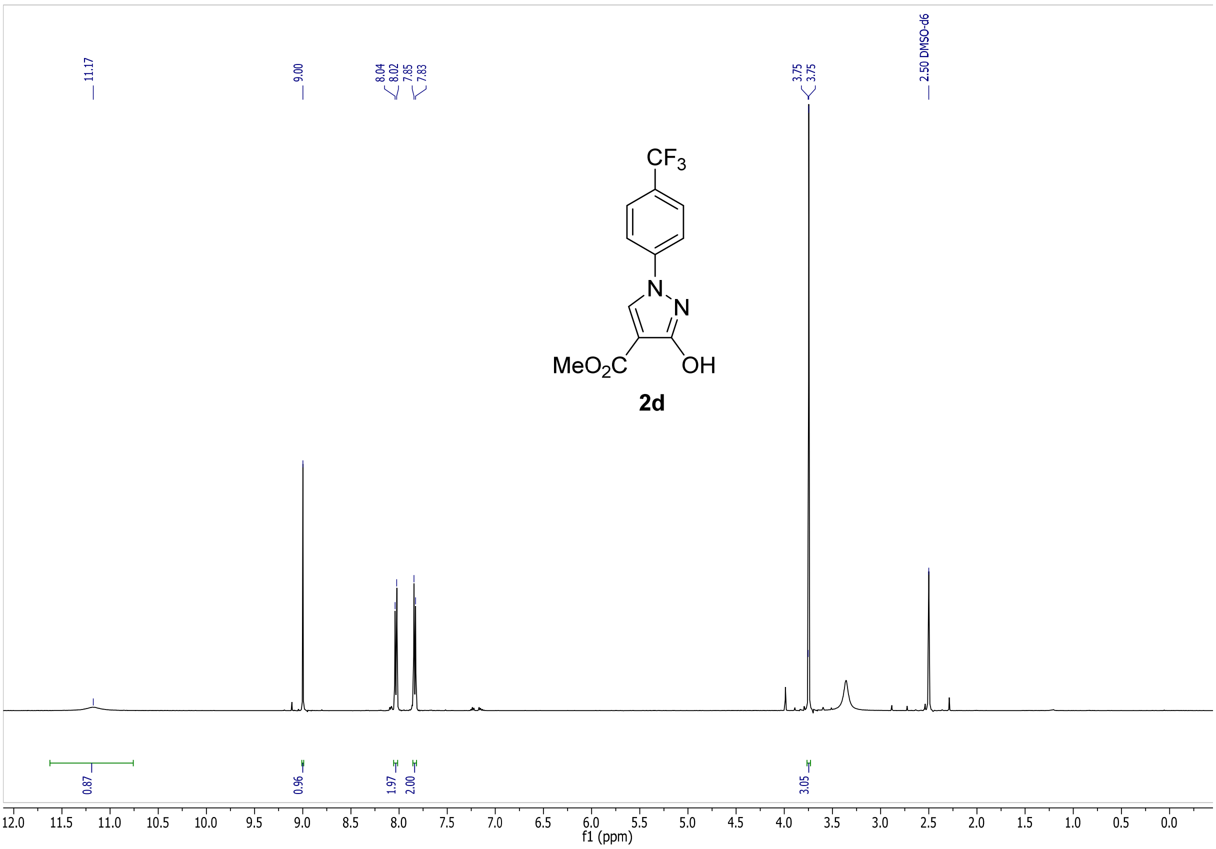
11.17
9.00
8.04
8.02
7.85
7.83
3.75
3.75
2.50 DMSO-d6
CF3
N
N
MeO2C
OH
2d
0.87
0.96
1.97
2.00
3.05
12.0 11.5 11.0 10.5 10.0 9.5 9.0 8.5 8.0 7.5 7.0 6.5 6.0 5.5 5.0 4.5 4.0 3.5 3.0 2.5 2.0 1.5 1.0 0.5 0.0
f1 (ppm)

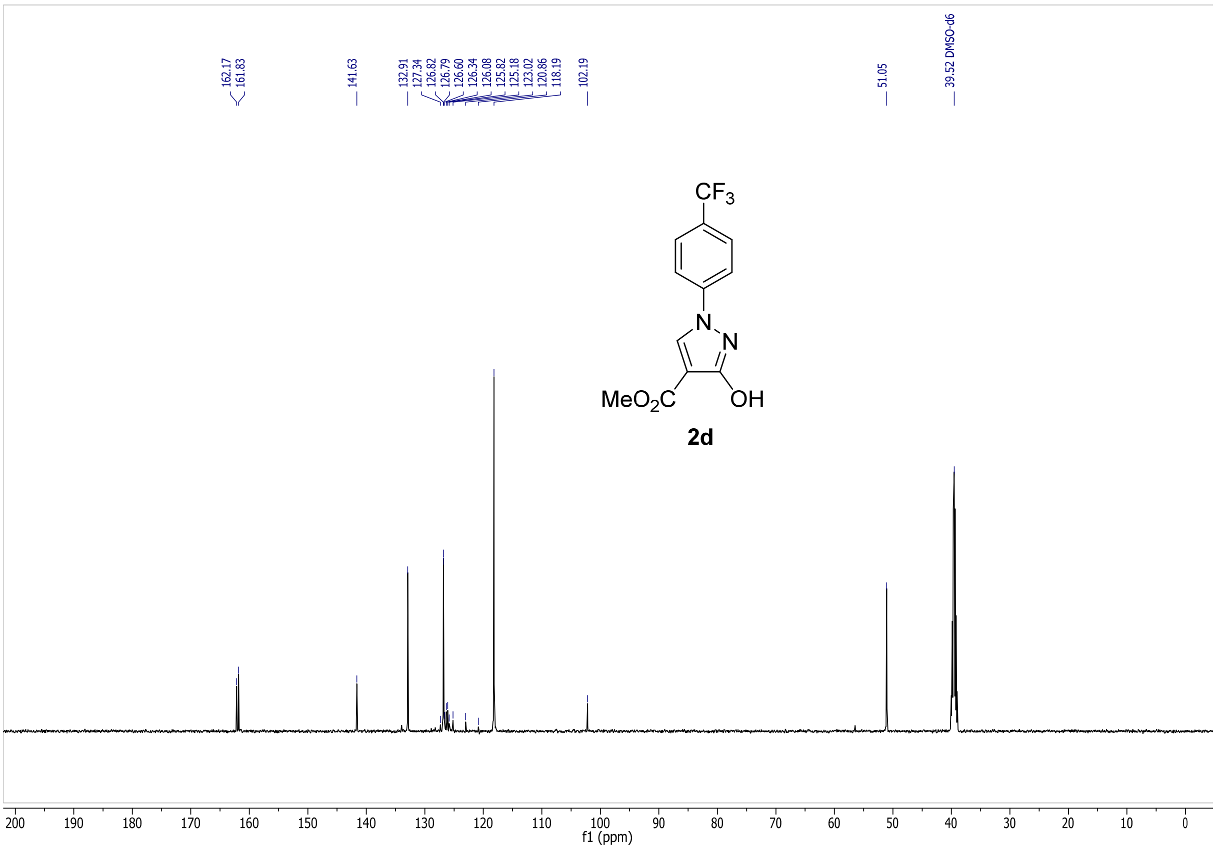
162.17
161.83
141.63
132.91
127.34
126.82
126.79
126.60
126.34
126.08
125.82
125.18
123.02
120.86
118.19
102.19
51.05
39.52 DMSO-d6
CF3
N
N
MeO2C
OH
2d
200 190 180 170 160 150 140 130 120 110 100 90 80 70 60 50 40 30 20 10 0
f1 (ppm)

1. Scores for evaluation of binding affinities compounds 3c, 1a–d, 2a–d, 5a–d, 10a–d, A, C, D, and 12 by quantum chemical methods.

Table S1. Scores for evaluation of binding affinities compounds **3c**, **1a–d**, **2a–d**, **5a–d**, **10a–d**, **A–E**, and **12** by quantum chemical methods.

Compound	*Glide SP*				*Glide XP*			
	Tot Q	Dscore[1]	GlideSP	Penality	Tot Q	DScore[1]	XP GScore	Penalty
**5a**	0	-6.57	-6.57	0.00	0	-6.68	-6.68	0.00
**5b**	0	-3.58	-3.58	0.00	0	-5.00	-5.00	0.00
**5c**	0	-6.57	-6.57	0.00	0	-5.01	-5.01	0.00
**5d**	0	-7.06	-7.06	0.00	0	-5.90	-5.90	0.00
**1a**	-1	-7.80	-7.89	0.09	–1	-6.20	-6.29	0.09
**1b**	-1	-6.87	-7.07	0.20	0	-6.01	-6.75	0.74
**1c**	0	-6.13	-7.52	1.39	0	-5.52	-6.90	1.39
**1d**	-1	-7.68	-7.93	0.24	–1	-6.99	-7.24	0.24
**10a**	0	-6.43	-6.43	0.00	0	-5.66	-5.66	0.00
**10b**	0	-6.05	-6.05	0.00	0	-5.30	-5.30	0.00
**10c**	0	-4.97	-4.97	0.00	0	-5.98	-5.98	0.00
**10d**	0	-6.49	-6.49	0.00	0	-4.71	-4.71	0.00
**2a**	-1	-8.39	-8.82	0.43	0	-6.65	-7.07	0.42
**2b**	-1	-7.68	-8.17	0.49	-1	-6.36	-6.85	0.49
**2c**	-1	-7.97	-8.40	0.44	0	-7.88	-8.28	0.40
**2d**	-1	-8.28	-8.90	0.61	0	-6.90	-7.27	0.38
**3c**	0	-6.44	-6.44	0.00	0	-6.40	-6.40	0.00
**A**	-1	-7.68	-8.17	0.49	-1	-6.36	-6.85	0.49
**C**	-1	-8.79	-8.84	0.06	-1	-8.54	-8.59	0.06
**D**	-1	-6.14	-7.31	1.17	0	-7.31	-7.40	0.09
**12**	-1	-9.96	-9.97	0.01	-1	-11.38	-11.38	0.00

[1] DScore = Glide + Penality.

3. Copies of IR spectra of compounds 1a–d, 2a–d, 5a–d, and 10a–d.

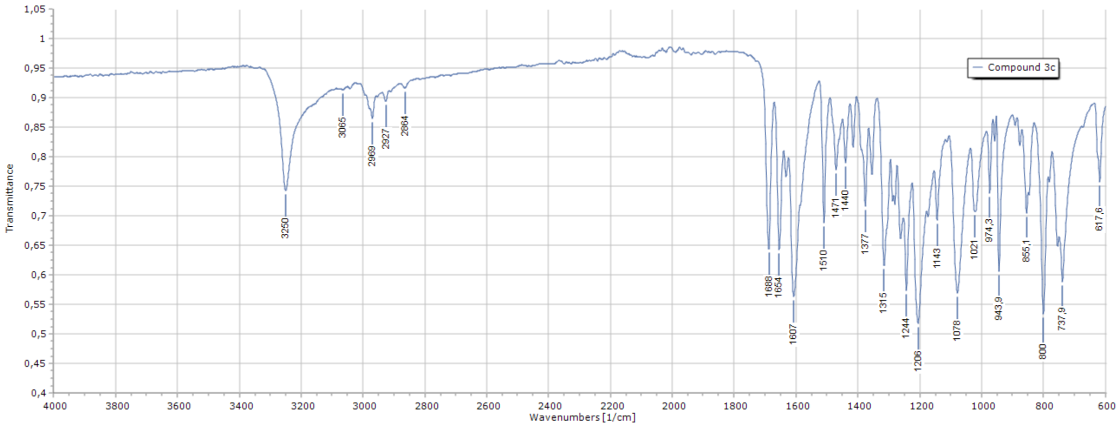

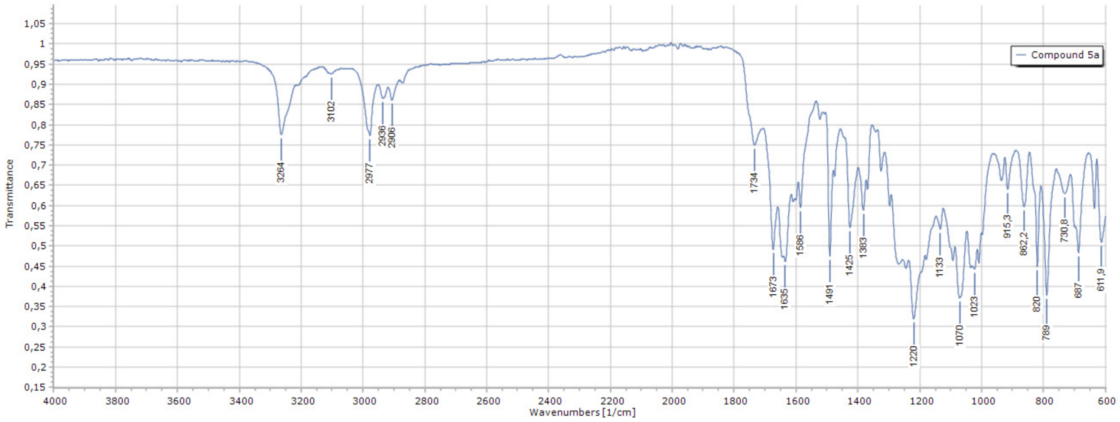

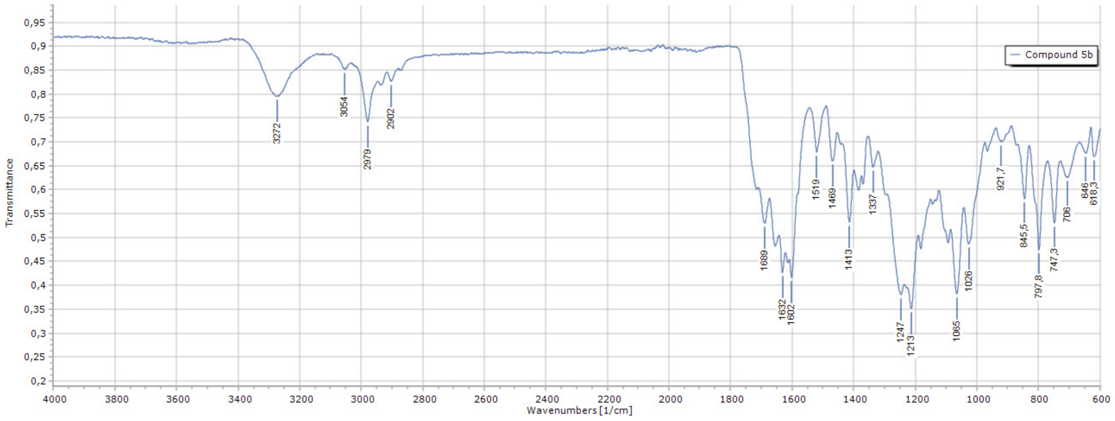

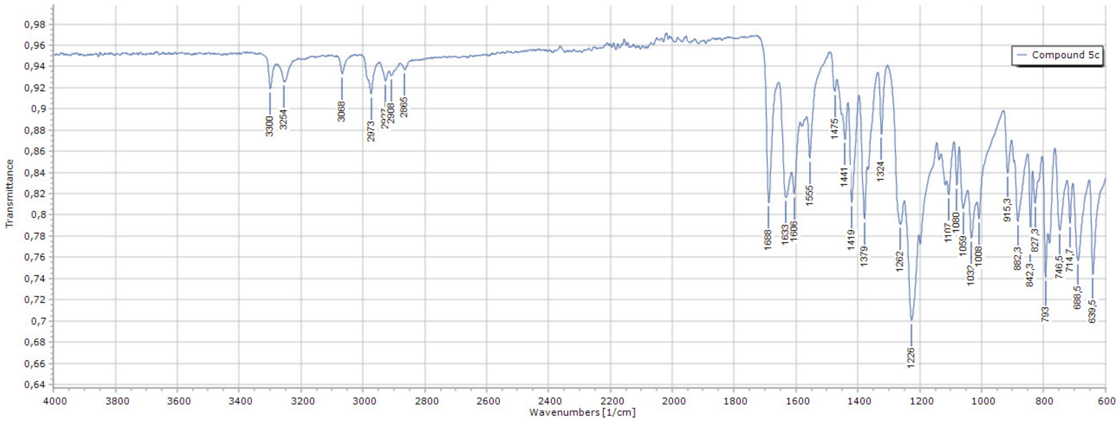

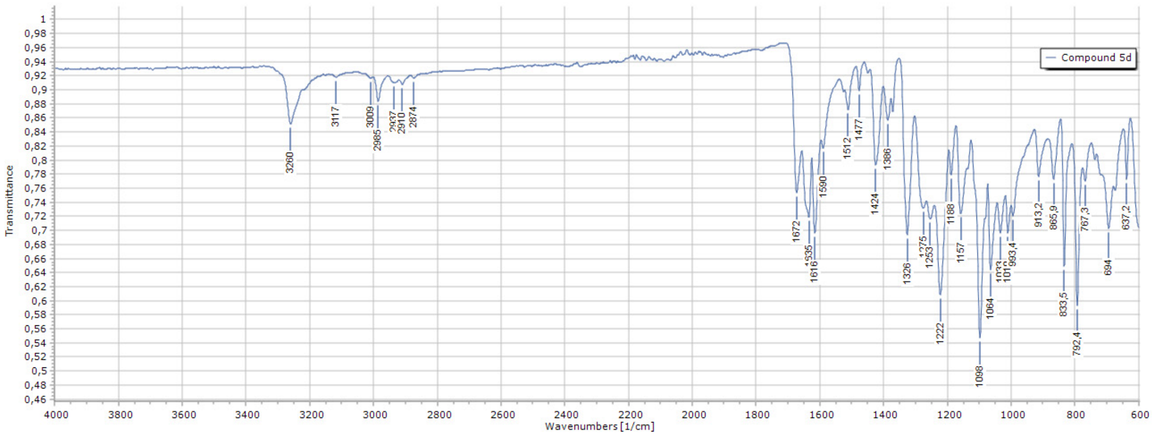
Compound 5d
Transmittance
Wavenumbers [1/cm]

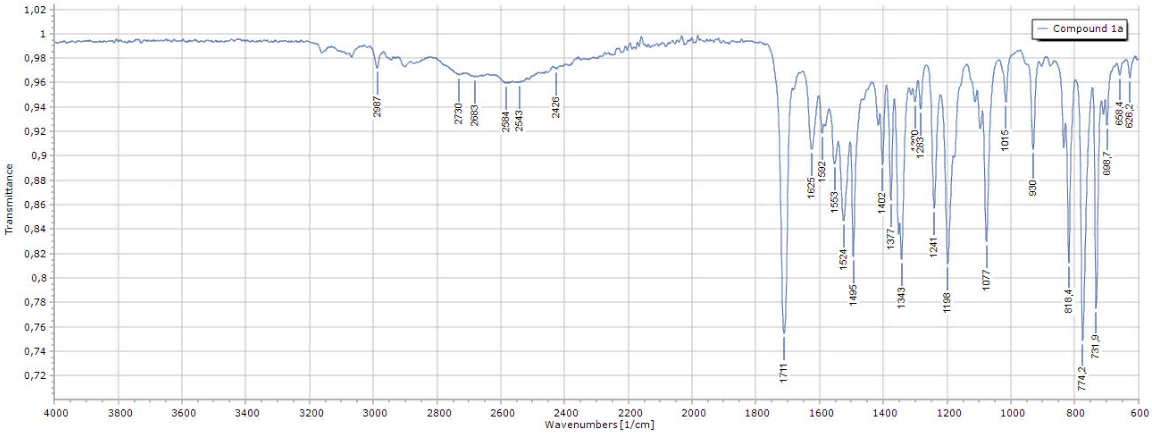
Compound 1a
Transmittance
Wavenumbers [1/cm]

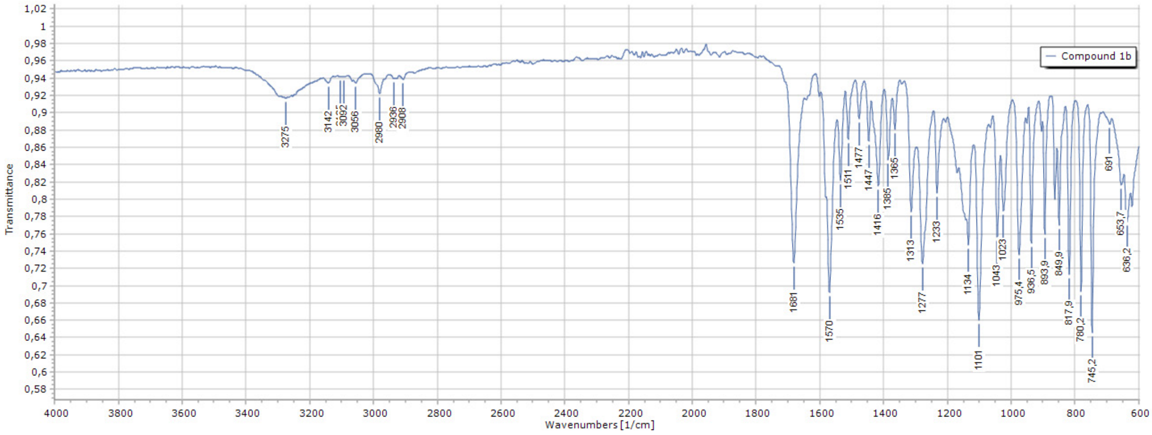
Compound 1b
Transmittance
Wavenumbers [1/cm]

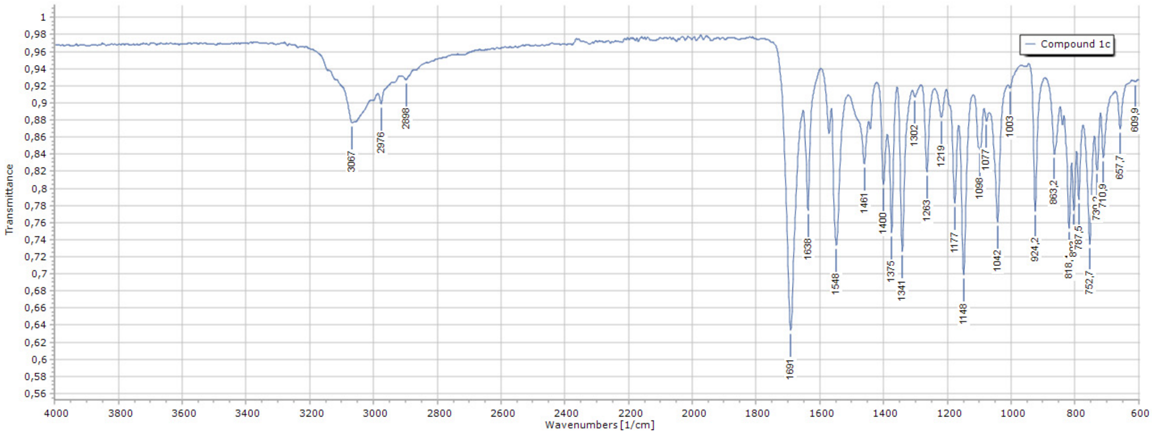
Compound 1c
Transmittance
Wavenumbers [1/cm]

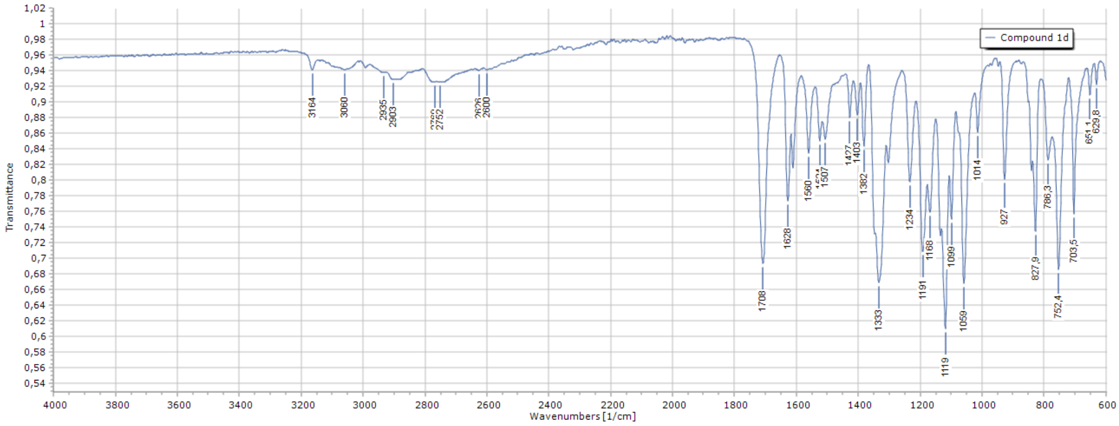
Compound 1d
Transmittance
Wavenumbers [1/cm]

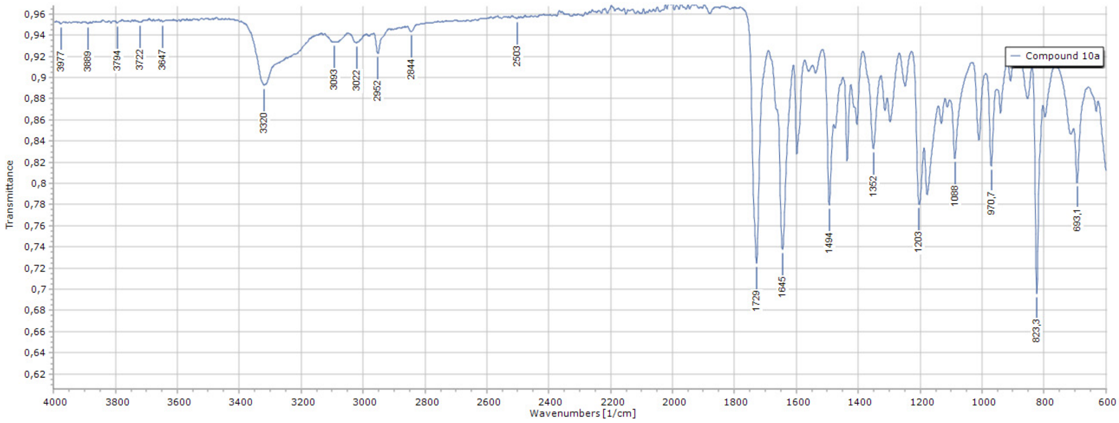
Compound 10a
Transmittance
Wavenumbers [1/cm]

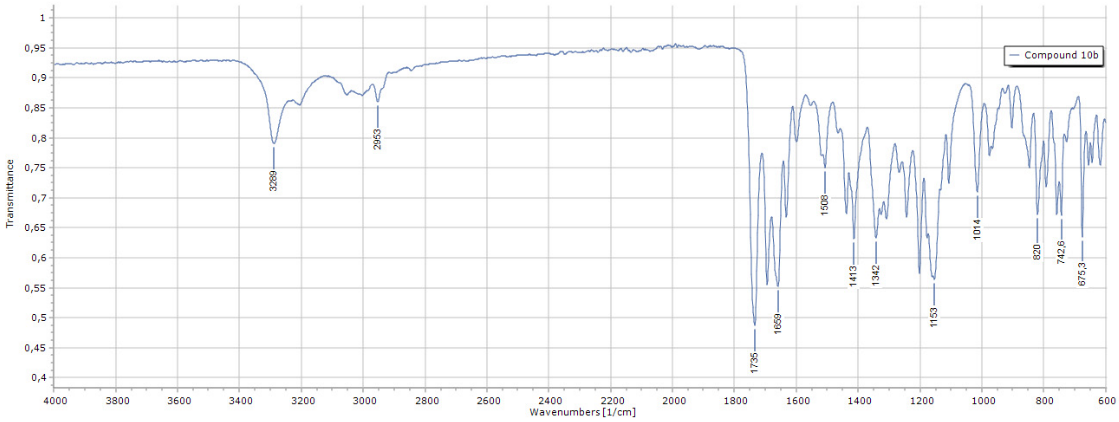
Compound 10b
Transmittance
Wavenumbers [1/cm]

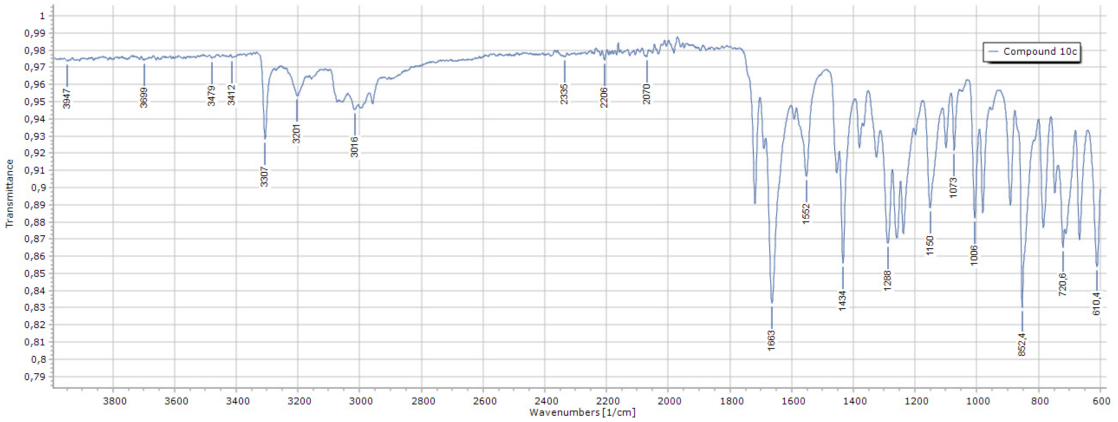
Compound 10c
Transmittance
Wavenumbers [1/cm]

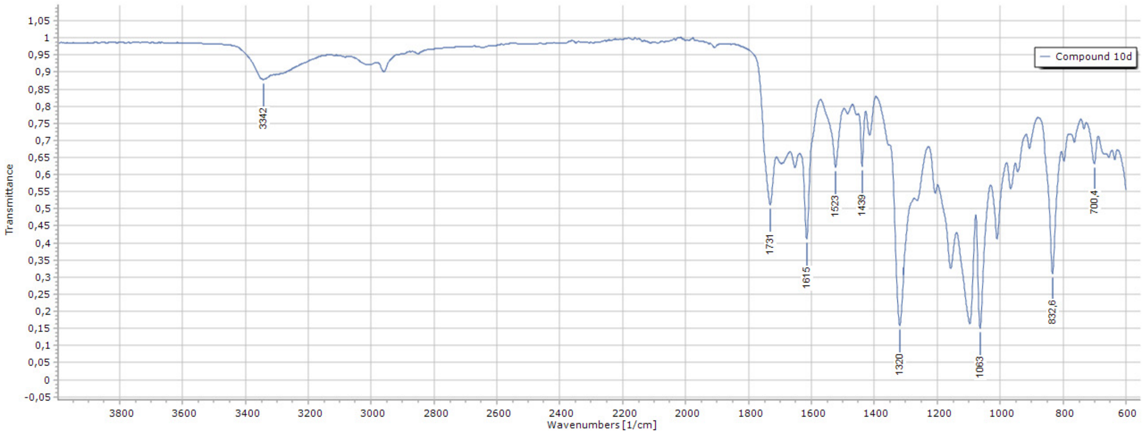
Compound 10d
Transmittance
Wavenumbers [1/cm]
3342
1731
1615
1523
1439
1320
1063
832,6
700,4

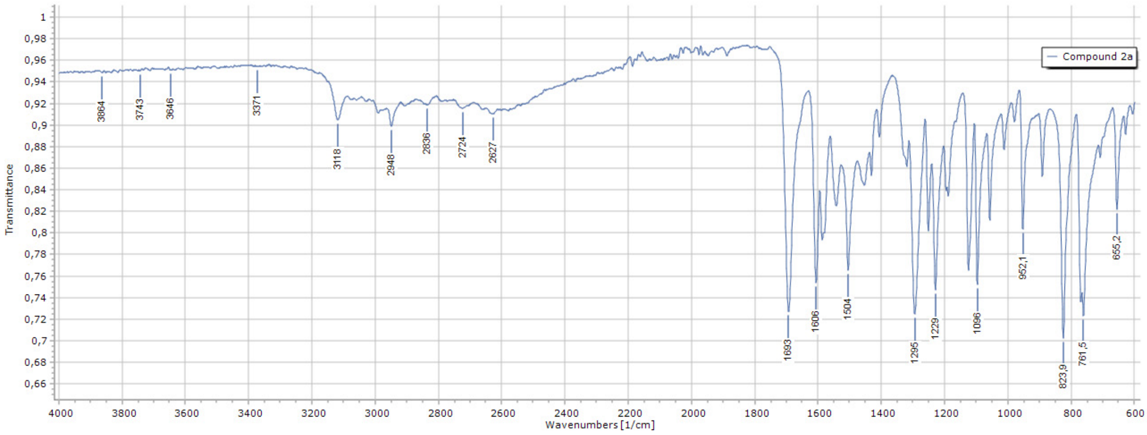
Compound 2a
Transmittance
Wavenumbers [1/cm]
3864
3743
3646
3371
3118
2948
2836
2724
2627
1693
1606
1504
1295
1229
1096
952,1
823,9
761,5
655,2

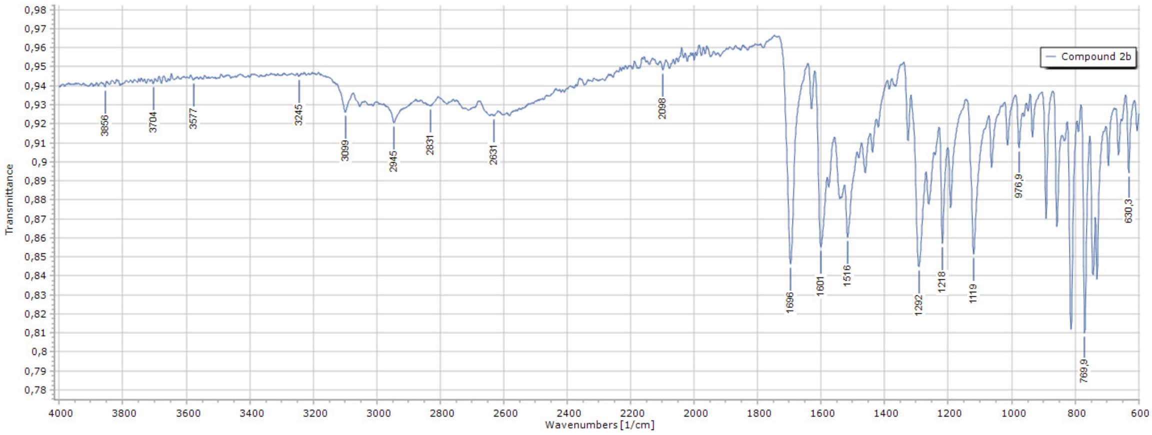
Compound 2b
Transmittance
Wavenumbers [1/cm]
3856
3704
3577
3245
3099
2945
2831
2631
2098
1696
1601
1516
1292
1218
1119
976,9
769,9
630,3

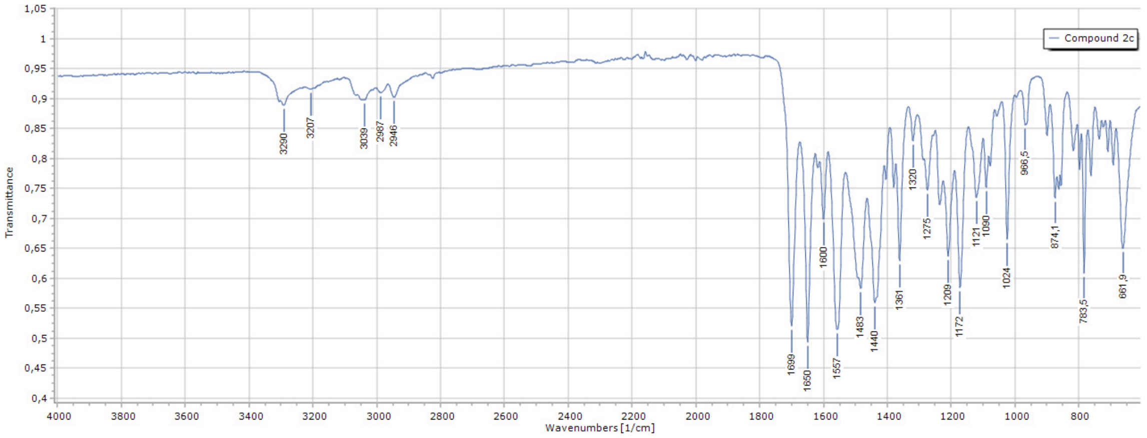
Compound 2c
Transmittance
Wavenumbers [1/cm]
3290
3207
3039
2987
2946
1699
1650
1600
1557
1483
1440
1361
1320
1275
1209
1172
1121
1090
1024
966,5
874,1
783,5
661,9

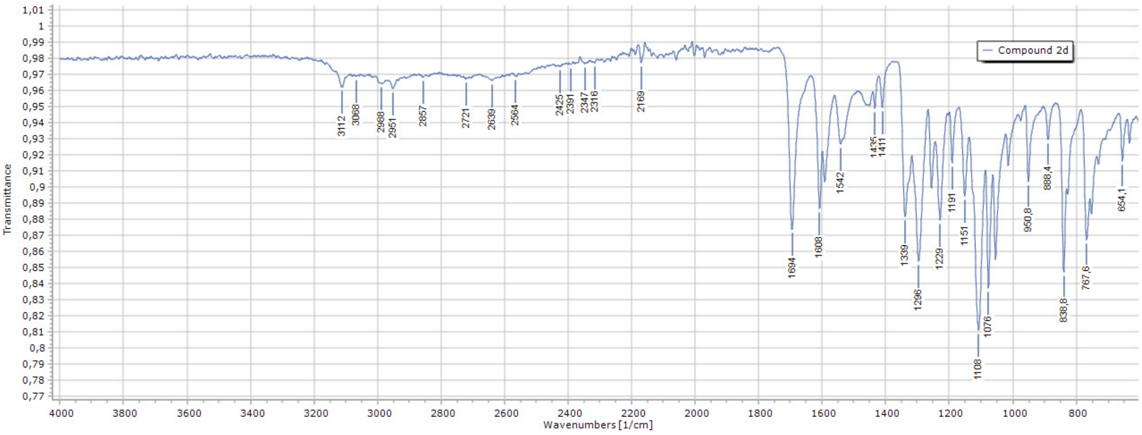
Compound 2d
Transmittance
Wavenumbers [1/cm]
3112
3068
2988
2951
2857
2721
2639
2564
2425
2391
2347
2316
2169
1694
1608
1542
1435
1411
1339
1296
1229
1191
1151
1108
1076
950,8
888,4
838,8
767,6
654,1
